# Effect of hyperhomocysteinemia on a murine model of smoke-induced pulmonary emphysema

**DOI:** 10.1038/s41598-022-16767-2

**Published:** 2022-07-28

**Authors:** Hiroshi Nakano, Sumito Inoue, Yukihiro Minegishi, Akira Igarashi, Yoshikane Tokairin, Keiko Yamauchi, Tomomi Kimura, Michiko Nishiwaki, Takako Nemoto, Yoichiro Otaki, Masamichi Sato, Kento Sato, Hiroyoshi Machida, Sujeong Yang, Hiroaki Murano, Masafumi Watanabe, Yoko Shibata

**Affiliations:** 1 Department of Cardiology, Pulmonology, and Nephrology, Yamagata University Faculty of Medicine, 2-2-2 Iida-Nishi, Yamagata, 990-9585 Japan; 2 Department of Pulmonary Medicine, Fukushima Medical University, Fukushima, Japan

**Keywords:** Experimental models of disease, Inflammation, Respiratory tract diseases

## Abstract

Hyperhomocysteinemia was reported to enhance endoplasmic reticulum (ER) stress and subsequent apoptosis in several cells. However, the precise mechanisms of smoking susceptibility associated with hyperhomocysteinemia has not been fully elucidated. This study included 7- to 9-week-old C57BL6 male mice induced with hyperhomocysteinemia and were exposed to cigarette smoke (CS). A549 cells (human alveolar epithelial cell line) were cultured with homocysteine and were exposed to cigarette smoke extract (CSE) to observe cell viability and expression of proteins related to the ER stress. After 6 months of CS exposure, pulmonary emphysema was more severely induced in the group under the condition of hyperhomocysteinemia compared to that in the control group. The apoptotic A549 cells increased as homocysteine concentration increased and that was enhanced by CSE. Protein expression levels of ER stress markers were significantly increased after simultaneous stimulation. Notably, vitamin B12 and folate supplementation improved ER stress after simultaneous stimulation of A549 cells. In this study, we showed that hyperhomocysteinemia exacerbates CS exposure-induced emphysema in mice, suggesting that hyperhomocysteinemia and CS stimulation enhance ER stress and subsequent induced apoptosis in alveolar epithelial cells. It was suggested that there is a synergistic effect between homocysteine and CS.

## Introduction

According to the Global Initiative for Chronic Obstructive Lung Disease 2020 Report, chronic obstructive pulmonary disease (COPD) is defined as a common, preventable, and treatable disease characterized by persistent respiratory symptoms and airflow limitation due to airway and/or alveolar abnormalities^1^. The World Health Organization reported that COPD is the third leading cause of death worldwide and is likely to increase in the coming years due to higher smoking prevalence and aging populations in many countries^2^. The main risk factor for COPD is cigarette smoking^3^. Only a fraction of smokers was often acknowledged to develop airflow obstruction^4,5^, and they are called susceptible smokers. Although genetic predisposition was reported to be associated with smoking susceptibility^6^, the risk factors predisposing to smoking susceptibility are not yet fully elucidated.

Histopathologic features of COPD include emphysema^7^, which results from alveolar cell apoptosis^8^. Emphysematous lung destruction reduces maximum expiratory flow by decreasing the elastic recoil force available to drive air out of the lungs^9^. An inverse correlation between emphysema lesions and forced expiratory volume in one second (FEV_1_) was reported^10^.

In our previous study, higher homocysteine level in the blood was reported to be associated with a rapid decline in FEV_1_ among male smokers in a general Japanese population^11^. In the patients with COPD, plasma homocysteine levels were significantly higher than in those of non-COPD patients, and plasma homocysteine levels were correlated with serum CRP levels and COPD severity^12^.

Homocysteine is a sulfur-containing amino acid formed during methionine metabolism, an essential amino acid^13^. Recently, homocysteine was reported to be involved in lung diseases^14^. A meta-analysis demonstrated that serum homocysteine could be a useful predictor for COPD development^15^. High-concentration homocysteine stimulation induces endoplasmic reticulum (ER) stress in cells such as the human umbilical vein endothelial cell and neural cell^16,17^, leading to apoptosis^18^.

The ER plays several roles in the biosynthesis, folding, assembly, and modification of numerous soluble proteins and membrane proteins^19^. Physiological states that increase the demand for protein folding, or stimuli that disrupt the reactions by which proteins fold, resulted in an imbalance between the protein folding load and the ER capacity, causing misfolded or unfolded proteins to accumulate in the ER lumen, so-called ER stress^20^. The ER responds to the burden of unfolded proteins by activating intracellular signal transduction, or unfolded protein response^21^, to restore cellular homeostasis or induce apoptosis if ER stress remains unmitigated^22^.

Cell injury secondary to chronic ER stress was reported to contribute to the pathophysiology of a wide range of human diseases including diabetes mellitus, stroke, heart disease, and cancer. Recently, ER stress is focused in the field of lung disease, particularly COPD^23,24^. Since CS contains a complex mixture of over 7,000 potentially harmful components^25^, cigarette smoking was suggested to induce ER stress^26,27^. These findings suggested the involvement of homocysteine in smoking susceptibility and in COPD pathogenesis.

According to these findings, this study was conducted to examine whether hyperhomocysteinemia exacerbate lung emphysematous changes by cigarette smoke (CS) exposure via ER stress and subsequent alveolar cell apoptosis.

## Result

### Plasma homocysteine concentrations in mice

**Figure 1:**
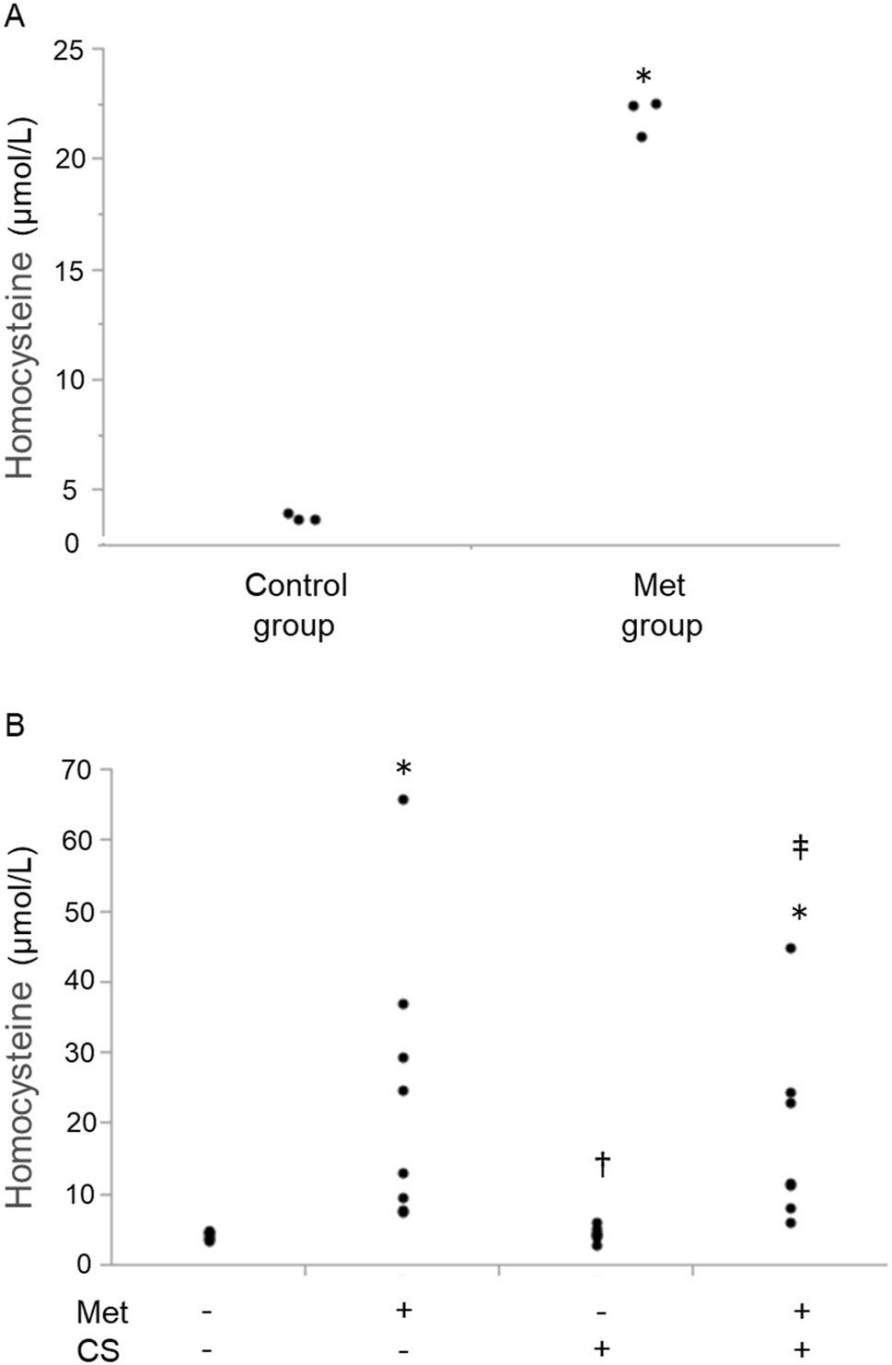
(**A**) Plasma homocysteine level in mice. After 5 days of administration of water or 1% of methionine solutions, plasma homocysteine levels in mice treated with methionine were significantly higher than those in mice treated with water. n = 3 in each group. Differences between groups were examined using t-test. *p < 0.05 vs control group. Met = l-methionine at 1% concentrations in water. (**B**) Difference in plasma homocysteine level between water and 1% methionine water-fed mice after 6 months of cigarette smoke exposure. Met− group, normal water administration; Met+ group, 1% methionine water administration; CS− group, air exposure; and CS+ group, cigarette smoke exposure. Plasma homocysteine concentrations were compared after 6 months of treatment. Significant increases in plasma homocysteine level were observed in Met+/CS− group and Met+/CS+ group compared to Met−/CS− group. In contrast, no significant difference was found in plasma homocysteine level between Met−/CS− group and Met−/CS+ group. n = 8 in each group. Differences between groups were examined using Bonferroni/Dunn test. *P < 0.05 vs Met−/CS−, ^†^P < 0.05 vs Met+/CS−, ^‡^P < 0.05 vs Met−/CS+.

Plasma homocysteine level was significantly elevated 5 days after 1% methionine (Met) water feeding (Fig. 1A). After 5 days of administration of water or 1% of methionine solutions, plasma homocysteine levels in mice treated with Met were significantly higher than those in mice treated with water (Met; 22.1 ± 0.5 µmol/L, water; 3.8 ± 0.1 µmol/L). To clarify the impact of cigarette smoke on the plasma homocysteine level, plasma levels of homocysteine in mice exposed to CS/room air for 6 months in the condition with/without 1% Met water feeding were measured. As shown in Fig. 1B, the significant increases in plasma homocysteine level were observed in Met+/CS− group (19.1 (8.3–35.1) µmol/L, (median (interquartile range))) and Met+/CS+ group (11.5 (8.9–24.1) µmol/L) compared to that in the control groups (Met−/CS− (4.4 (4.0–4.8) µmol/L) and Met−/CS+ (4.7 (4.3–5.2) µmol/L)). Conversely, no significant difference was found in plasma homocysteine level between Met−/CS− group and Met−/CS+ group, indicating that 6 months of cigarette smoke exposure alone did not elevate plasma homocysteine level.

### Cigarette smoke exposure with hyperhomocysteinemia enhanced lung emphysematous changes

**Figure 2:**
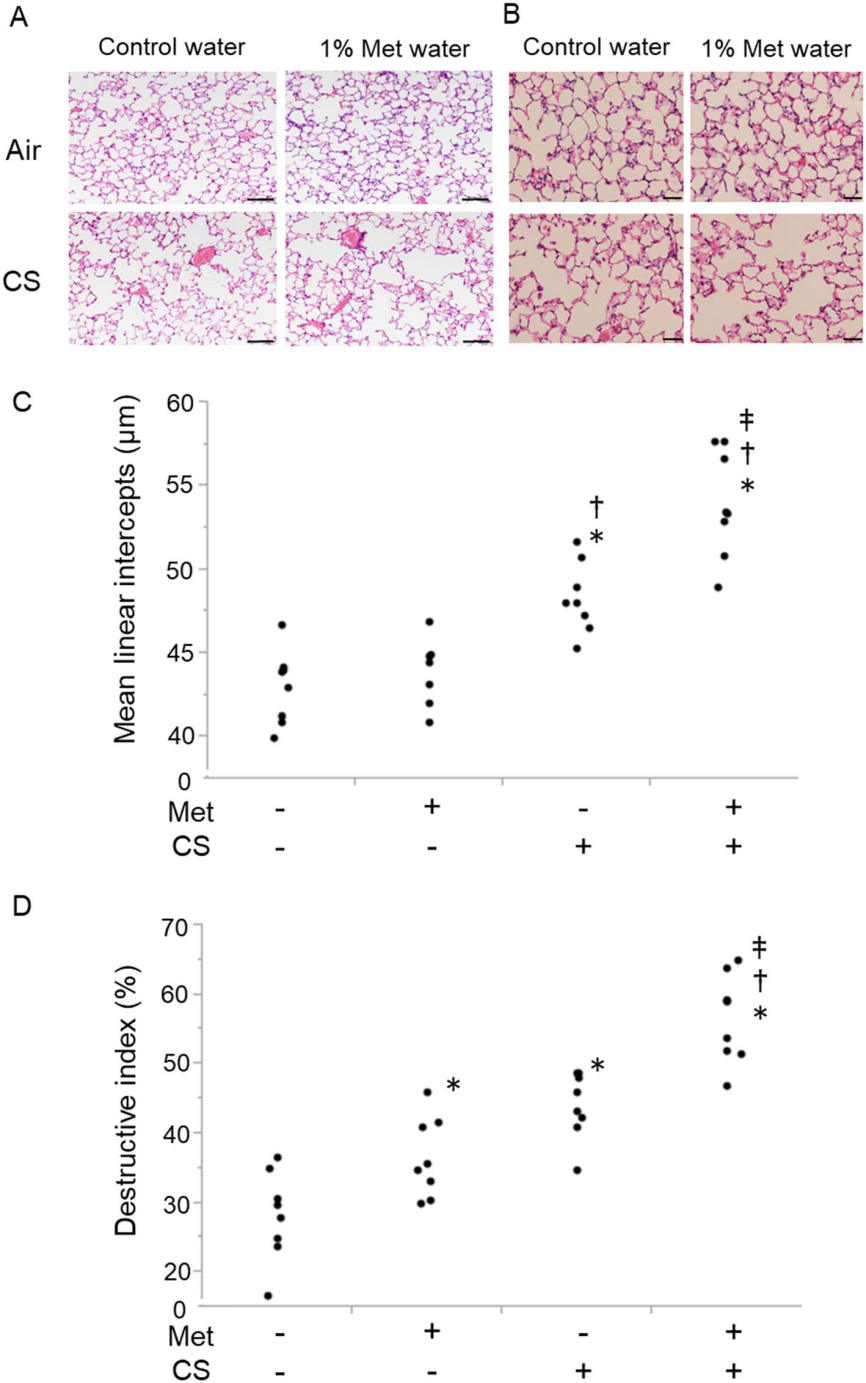
Pathological examination of lung emphysematous changes in mice by hematoxylin and eosin (H&E) staining. (**A**) At 200 × magnification. Scale bar means 100 µm. (**B**) At 400× magnification. Scale bar means 40 µm. Lung sections stained with H&E were shown. Dense alveolar structure is observed in the lung of mice given with normal water and exposed to air. Histological images of mice given with 1% methionine water and exposed to air is not different from histological images of the lung in mice given with normal water and exposed to air. Emphysematous changes and inflammatory cells are observed in the lung of mice given with normal water and exposed to cigarette smoke (CS). In mice given with 1% methionine water and exposed to CS, enhanced emphysematous changes compared with histological images of the lung in mice given with normal water are observed. Also, inflammatory cells and alveolar-wall destruction are observed. (**C**) Difference in mean linear intercept (MLI) in the lung section between water and 1% methionine water-fed mice 6 months after cigarette smoke exposure. Met− group, normal water administration; Met+ group, 1% methionine water administration; CS− group, air exposure; and CS+ group, cigarette smoke exposure. The MLI was significantly increased in the Met−/CS+group (48.30 ± 0.75 μm), compared with that in the Met−/CS− group (42.96 ± 0.77 μm), (P = 0.0007). Conversely, MLIs in the Met+/CS− group (43.61 ± 0.70 μm) were not increased compared to those in the control (Met−/CS−) group (P = 0.9487). Further increase was observed in the Met+/CS+ group (53.93 ± 1.13 μm), (P < 0.0001 vs Met−/CS− group, P < 0.0001 vs Met+/CS− group, P = 0.0004 vs Met−/CS+group). n = 8 in each group. Differences between groups were examined using Tukey–Kramer test. *P < 0.05 vs Met−/CS−, ^†^P < 0.05 vs Met+/CS−, ^‡^P < 0.05 vs Met−/CS+. CS: cigarette smoke. Met: methionine. (**D**) Difference in destructive index (DI) in the lung section between water and 1% methionine water-fed mice 6 months after cigarette smoke exposure. Met− group, normal water administration; Met+ group, 1% methionine water administration; CS− group, air exposure; and CS+ group, cigarette smoke exposure. After 6 months of CS exposure, DI was significantly increased in the Met+/CS− group (36.50 ± 2.04%), (P = 0.0381), Met−/CS+ group (44.08 ± 1.70%), (P < 0.0001), and Met+/CS+ group (56.38 ± 2.26%), (P < 0.0001) compared to control (Met−/CS−) group (28.10 ± 2.28%). DI in Met+/CS+ group was significantly more than in other groups. n = 8 in each group. Differences between groups were examined using Tukey–Kramer test. *P < 0.05 vs Met−/CS−, ^†^P < 0.05 vs Met+/CS−, ^‡^P < 0.05 vs Met−/CS+. *CS* cigarette smoke, *Met* methionine.

To examine the impact of hyperhomocysteinemia in the development of emphysematous change in the lungs (Fig. 2A,B), the difference of mean linear intercept (MLI) among four groups (Fig. 2C) was assessed. After 6 months of CS exposure, emphysematous changes were observed in the CS with control water group, but deteriorated in the CS with 1% Met water group. The MLI was significantly increased in the Met−/CS+ group (48.30 ± 0.75 μm), compared with that in the Met−/CS− group (42.96 ± 0.77 μm), (P = 0.0007). Conversely, MLIs in the Met+/CS− group (43.61 ± 0.70 μm) were not increased compared to those in the control (Met−/CS−) group (P = 0.9487). Further increase was observed in the Met+/CS+ group (53.93 ± 1.13 μm), (P < 0.0001 vs Met−/CS− group, P < 0.0001 vs Met+/CS− group, P = 0.0004 vs Met−/CS+ group).

Destructive index (DI) was also evaluated from tissue samples after smoking exposure (Fig. 2D). After 6 months of CS exposure, DI was significantly increased in the Met+/CS− group (36.50 ± 2.04%), (P = 0.0381), Met−/CS+ group (44.08 ± 1.70%), (P < 0.0001), and Met+/CS+ group (56.38 ± 2.26%), (P < 0.0001) compared to control (Met−/CS−) group (28.10 ± 2.28%). DI in Met+/CS+ group was significantly more than in other groups.

Therefore, CS alone induced pulmonary emphysema, but it enhanced emphysematous changes induced by addition of Met, suggesting the synergic effect of hyperhomocysteinemia and CS exposure in the development of emphysematous changes.

### Combination of homocysteine and cigarette smoke extract augmented A549 cell apoptosis

**Figure 3:**
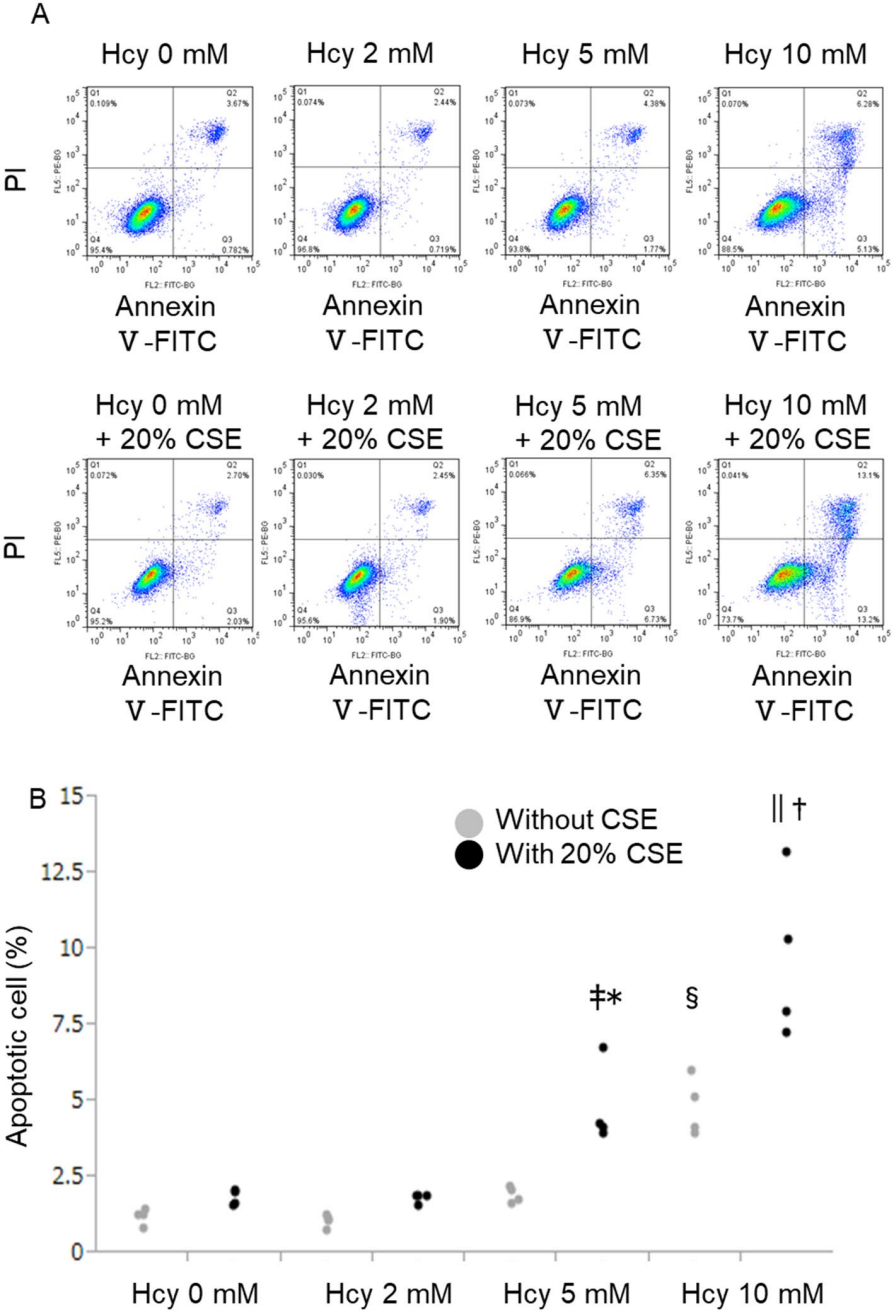
Effects of homocysteine and cigarette smoke extract (CSE) on the induction of A549 cells apoptosis. To investigate the synergic effect of homocysteine and cigarette smoke exposure on alveolar cell apoptosis in vitro, A549 cells were treated with 2 mM, 5 mM, or 10 mM homocysteine and 20% CSE for 24 h, followed by apoptosis assays using flow cytometry. (**A**) The apoptotic rates of A549 cells were evaluated using Annexin V/propidium iodide double staining. Apoptosis was regarded as the cell percentage in the Q3 quadrant. (**B**) Quantified data of flow cytometry. Quantified data of flow cytometry showed no significant difference in the percentage of apoptotic cells after only CSE administration. On the other hand, the percentage of apoptotic cells was gradually increased as homocysteine concentration increased, and significant elevation was observed in 10 mM of homocysteine. Simultaneous stimulation with homocysteine more than 5 mM and 20% CSE dramatically increased the percentage of apoptotic cells. n = 4 in each group. Differences between groups were examined using Tukey–Kramer test. *P < 0.05 vs homocysteine 5 mM without 20% CSE, ^†^P < 0.05 vs homocysteine 10 mM without 20% CSE, ^‡^P < 0.05 vs homocysteine 2 mM with 20% CSE, ^§^P < 0.05 vs homocysteine 5 mM without CSE, ^||^P < 0.05 vs homocysteine 5 mM with 20% CSE. *CSE* cigarette smoke extract, *Hcy* homocysteine, *PI* propidium iodide.

To clarify the synergic effects of homocysteine and cigarette smoke exposure on alveolar cell apoptosis in vitro, apoptosis in the cultured alveolar cells were assessed using flow cytometry after the treatment with homocysteine, CSE, or both (Fig. 3A). Sole addition of CSE into the cultured media did not induce the increase of apoptosis in this experiment. However, the percentage of apoptotic cells gradually increased as homocysteine concentration increased (Fig. 3B), and significant elevation was observed in 10 mM of homocysteine. Notably, simultaneous stimulation with homocysteine more than 5 mM and 20% CSE significantly increased the percentage of apoptotic cells. These results showed that combination of homocysteine and CSE enhanced an alveolar cell apoptosis.

### Synergic induction of endoplasmic reticulum stress caused by homocysteine and cigarette smoke extract

**Figure 4:**
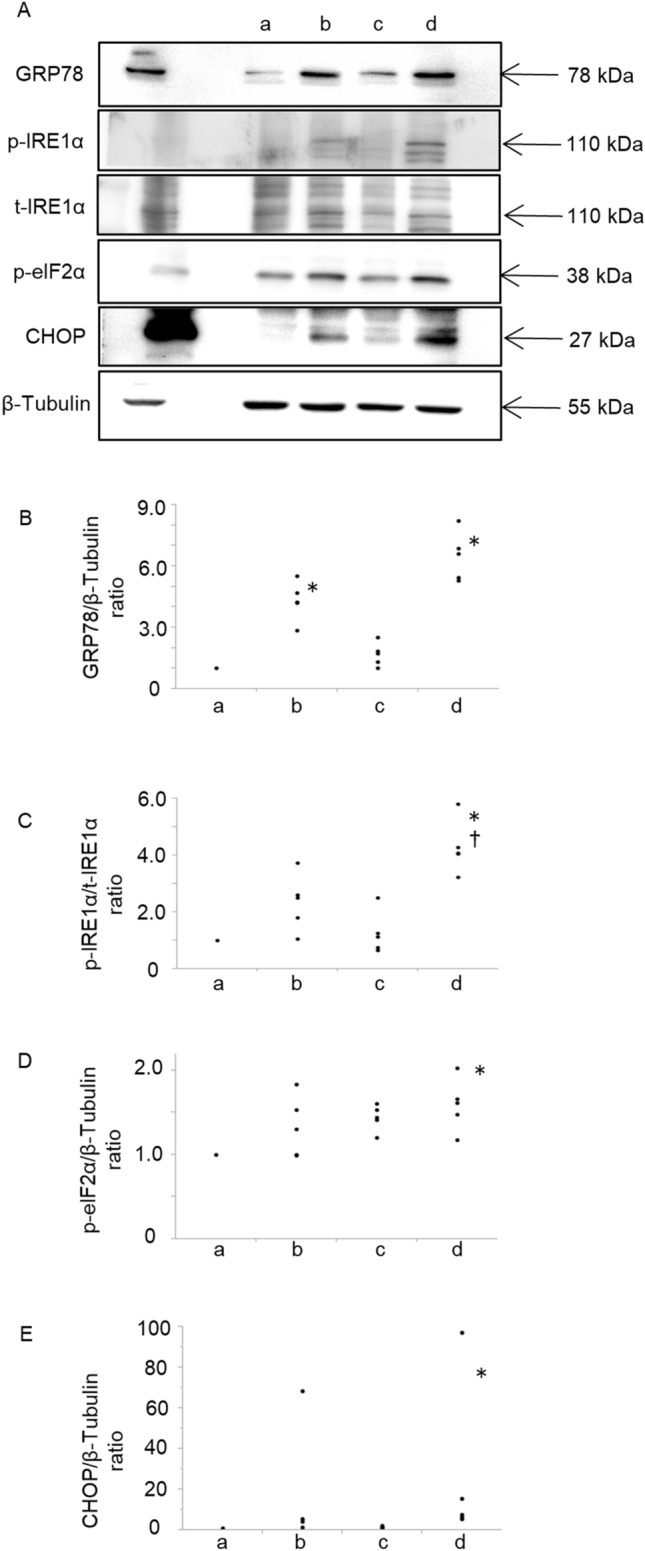
Synergic induction of endoplasmic reticulum (ER) stress by homocysteine and cigarette smoke extract (CSE) in A549 cells. To examine the impact of simultaneous stimulation of homocysteine and CSE on the ER stress, A549 cells were treated with 20% CSE and 5 mM homocysteine, and then the protein levels of GRP78, phosphorylation of IRE1α and p-eIF2α, protein level of CHOP was examined using western blotting. (**A**) Graphic depictions of western blotting. (**B**–**E**) Quantitative analysis for protein expression of GRP78, phosphorylation of IRE1α, phosphorylation of eIF2α, protein expression of CHOP by western blotting. The protein expression level of GRP78 (**B**) was significantly elevated in response to homocysteine stimulation. The protein expression levels of GRP78 (**B**), phosphorylation of IRE1α (**C**), and protein expression levels of p-eIF2α (**D**) and CHOP (**E**) were significantly elevated in response to the combined stimulation of homocysteine and CSE compared to control. a; control, b; homocysteine 5 mM, c; 20% CSE, d; homocysteine 5 mM + 20% CSE. n = 5 in each group. Differences between groups were examined using Bonferroni/Dunn test. *P < 0.05 vs homocysteine 0 mM, ^†^P < 0.05 vs 20% CSE. *CSE* cigarette smoke extract, *CHOP* CCAAT/enhancer binding protein homologous protein, *GRP78* 78-kDa glucose-regulated protein, *p-IRE1α* phosphorylation of inositol-requiring enzyme 1α, *p-eIF2α* phosphorylate eukaryotic translation initiation factor 2α.

The impact of simultaneous stimulation of homocysteine and CSE on the protein levels of ER stress-related molecules was examined (Fig. 4A). The protein expression levels of 78-kDa glucose-regulated protein (GRP78) (Fig. 4B) was significantly elevated in response to homocysteine stimulation. The protein expression levels of GRP78 (Fig. 4B), phosphorylation of inositol-requiring enzyme 1 α (IRE1α) (Fig. 4C), and protein expression levels of phosphorylate eukaryotic translation initiation factor 2α (p-eIF2α) (Fig. 4D) and CCAAT/enhancer binding protein homologous protein (CHOP) (Fig. 4E) were significantly elevated in response to the combined stimulation of homocysteine and CSE compared to control. These results indicated that CSE under the high homocysteine condition enhanced ER stress in A549 cells.

### Vitamin B12 and folate cotreatment attenuated endoplasmic reticulum stress induced by cigarette smoking exposure and homocysteine

**Figure 5:**
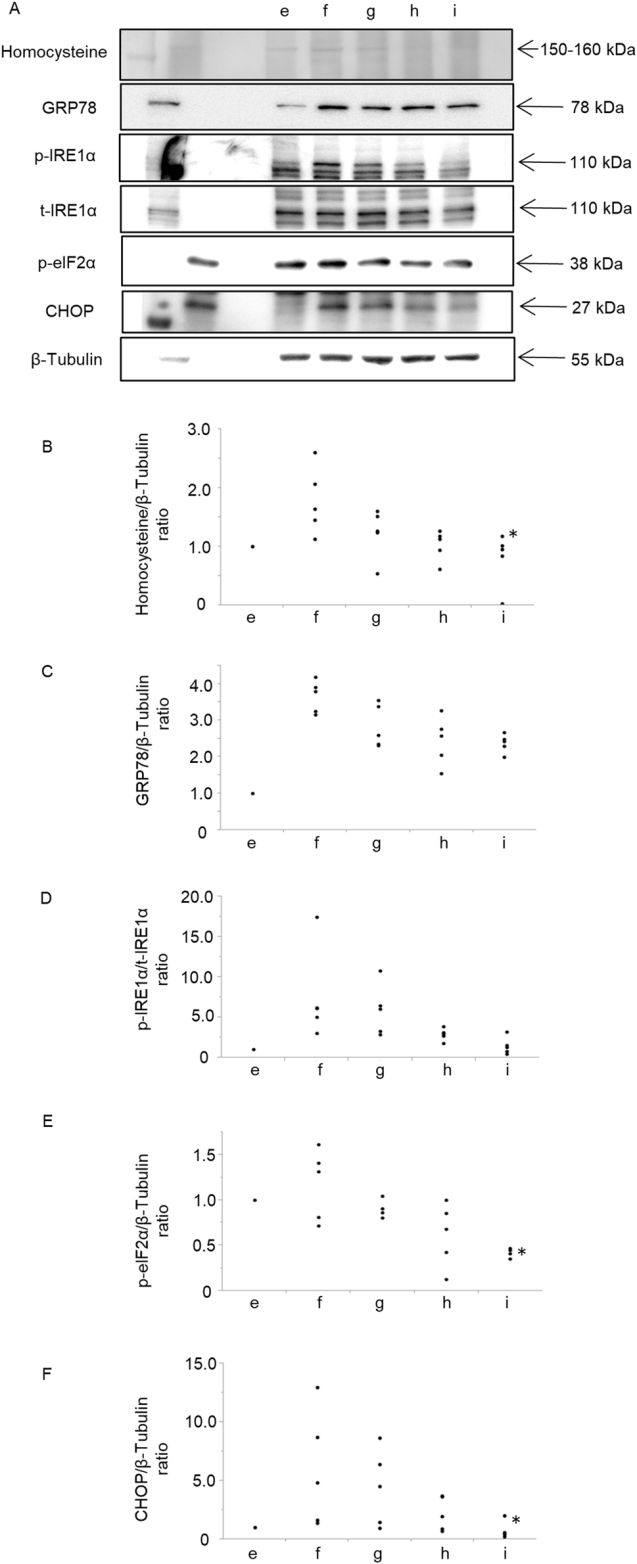
Vitamin B12 (VB) and folate (Fo) improved endoplasmic reticulum (ER) stress induced by homocysteine and cigarette smoke extract (CSE) in A549 cells. To investigate the effect of vitamin B12 and folate supplementation on the protein levels of ER stress-related molecules, A549 cells were pretreated with the same concentration of vitamin B12 and folate adjusted to 5‒50 μM for 12 h, followed by stimulation with 5 mM homocysteine plus 20% CSE. (**A**) Graphic depictions of western blotting. (**B**–**F**) Quantitative analysis for protein expression of homocysteine, GRP78, phosphorylation of IRE1α, expression of p-eIF2α, and CHOP by western blotting. The protein expression levels of homocysteine, p-eIF2α, and CHOP induced by CSE and homocysteine were significantly decreased by pretreatment of vitamin B12 and folate in a dose-dependent manner. e; 20% CSE, f; 20% CSE+ homocysteine 5 mM, g; 20% CSE+ homocysteine 5 mM+ vitamin B12 and folate 5 μM, h; 20% CSE+ homocysteine 5 mM+ vitamin B12 and folate 25 μM, i; 20% CSE+ homocysteine 5 mM+ vitamin B12 and folate 50 μM. n = 5 in each group. Differences between groups were examined using Bonferroni/Dunn test. *P < 0.05 vs 20% CSE+ homocysteine 5 mM. *CSE* cigarette smoke extract, *CHOP* CCAAT/enhancer binding protein homologous protein, *GRP78* 78-kDa glucose-regulated protein, *p-IRE1α* phosphorylation of inositol-requiring enzyme 1, *p-eIF2α* phosphorylate eukaryotic translation initiation factor 2α.

The effect of vitamin B12 and folate supplementation on the protein and phosphorylation levels of ER stress-related molecules was investigated (Fig. 5A). The protein expression levels of homocysteine, p-eIF2α, and CHOP induced by CSE and homocysteine were significantly decreased by pretreatment of vitamin B12 and folate in a dose-dependent manner (Fig. 5B–F). These findings demonstrated that vitamin B12 and folate restore ER stress caused by homocysteine/CSE stimulation.

### Cotreatment with vitamin B12 and folate reduced the proportion of apoptotic A549 cells induced by cigarette smoking exposure and homocysteine

**Figure 6:**
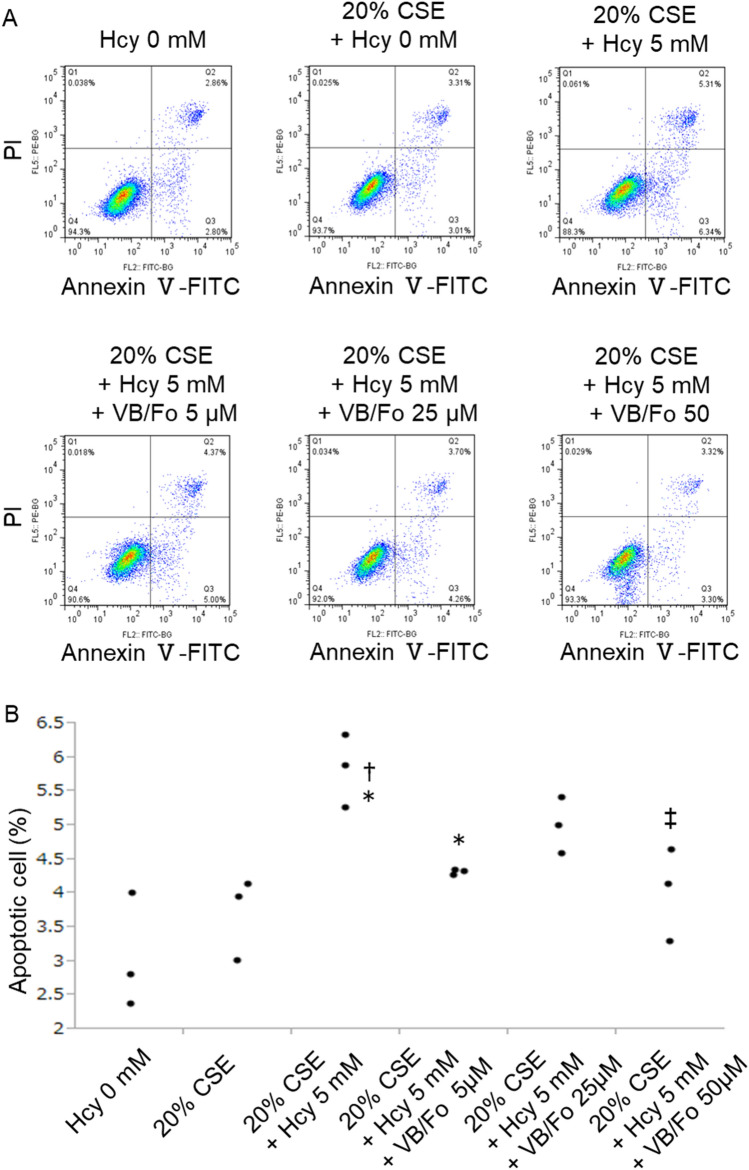
Vitamin B12 (VB) and folate (Fo) reduced the proportion of apoptotic A549 cells induced by homocysteine and cigarette smoke extract (CSE). To investigate the effect of vitamin B12 and folate supplementation on the apoptosis induced by homocysteine/CSE, A549 cells were pretreated with the same concentration of vitamin B12 and folate adjusted to 5‒50 μM for 12 h, followed by stimulation with 5 mM homocysteine plus 20% CSE. (**A**) The proportion of apoptotic A549 cells were evaluated using Annexin V/propidium iodide double staining. Apoptosis was regarded as the cell percentage in the Q3 quadrant. (**B**) Quantified data of flow cytometry. Quantified data of flow cytometry show that simultaneous stimulation with homocysteine 5 mM and 20% CSE increased the proportion of apoptotic cells. However, the proportion of apoptotic cells was decreased as the concentration of Vitamin B12 and folate increased. n = 3 in each group. Differences between groups were examined using Tukey–Kramer test. *P < 0.05 vs homocysteine 0 mM, ^†^P < 0.05 vs 20% CSE, ^‡^P < 0.05 vs 20% CSE + homocysteine 5 mM. *CSE* cigarette smoke extract, *Fo* folate, *Hcy* homocysteine, *PI* propidium iodide, *VB* vitamin B12.

Whether vitamin B12 and folate supplementation attenuated the apoptosis of A549 cells induced by homocysteine/CSE was evaluated. Flow cytometry showed the increased percentage of apoptotic cells induced by CSE/homocysteine improved by the pretreatment of vitamin B12 and folate in a dose-dependent manner (Fig. 6A,B).

## Discussion

The new and important findings from this study included the following: [1] Hyperhomocysteinemia mice exposed to CS for 6 months developed more severe emphysema compared to non-hyperhomocysteinemia mice exposed to CS. [2] The proportion of alveolar cell apoptosis was significantly increased in cells with simultaneous homocysteine and CSE stimulation than in those with sole stimulation. [3] The ER stress assessed using GRP78, phosphorylation of IRE1α, phosphorylation of eIF2α, and CHOP enhanced by simultaneous stimulation. [4] Lastly, ER stress and subsequent alveolar cell apoptosis caused by simultaneous stimulation were restored by vitamin B12 and folate supplementation. These results indicate that hyperhomocysteinemia exacerbates lung emphysematous changes by CS exposure via ER stress and subsequent alveolar cell death.

Several clinical studies have reported a positive correlation between cigarette smoking and blood homocysteine levels^28,29^, but these studies did not consider factors that increase plasma homocysteine levels such as blood vitamin B level and eating habits. On the other hand, an experimental report examined the thiol status in the blood of different mouse strains exposed to CS and demonstrated no increase in blood homocysteine levels after cigarette smoke exposure in all tested mice including C57BL/6J^30^. In accordance with this report, this study showed that plasma homocysteine concentrations in mice were not increased by CS exposure (Fig. 1B). Therefore, no causal relationship between CS exposure and plasma homocysteine levels in mice was found.

In this study, a proportion of apoptotic cells and expression of GRP78, one of the ER proteins related to apoptosis^16^, were increased in A549 cells with high homocysteine state. However, only DI showed a significant increase, but MLI did not, suggesting that it did not form sufficient emphysema in mice with hyperhomocysteinemia alone. Plasma homocysteine levels in mice induced by giving 1% l-methionine water corresponded to almost moderate hyperhomocysteinemia in human, and these levels of homocysteine alone was not assumed to lead to emphysema formation. Conversely, hyperhomocysteinemia in mice enhanced the pulmonary emphysema after 6 months of CS exposure (Fig. 2). Considering these findings, hyperhomocysteinemia may play an important role in smoking susceptibility for inducing emphysematous lesion in the lung.

In mammalian cells, three ER transmembrane transducers have been elucidated as unfolded protein response sensors: protein kinase R (PKR)-like ER kinase (PERK), IRE1α and Activating transcription factor 6 (ATF6)^21,31^. PERK pathway activates the apoptosis cascade by upregulating CHOP transcription. IRE1α also activates an apoptotic kinase, c-jun N-terminal kinase, through the signal cascade of the tumor necrosis factor receptor-associated factor 2-apoptosis signal-regulating kinase 1 pathway^32^. This study showed that expression of GRP78, p-IRE1α, p-eIF2 and CHOP were significantly increased in response to simultaneous homocysteine and CSE stimulation, suggesting the involvement of PERK and IRE1α pathway in alveolar cell apoptosis (Fig. 4). Recently, homocysteine-induced ER stress and apoptosis were rescued by blocking the PERK pathway, suggesting the importance of PERK pathway under the hyperhomocysteinemia^33^. In contrast, in this study, CSE alone did not induce statistically significant apoptosis or ER stress-related molecules such as GRP78, p-IRE1α, p-eIF2α, and CHOP expression in A549 cells. Although the concentration of CSE was considered to be enough to induce apoptosis^34^, this CSE concentration (20%) was not sufficient to cause apoptosis or ER stress alone in this study. Under the hyperhomocysteinemic condition, the effects of CS exposure on the induction of ER stress may be greatly enhanced through activating ER transmembrane transducers, finally leading to pulmonary emphysema. Homocysteine may enhance smoking susceptibility via ER stress-induced alveolar cell apoptosis.

In this study, ER stress and subsequent alveolar cell apoptosis were restored by vitamin B12 and folate supplementation (Figs. 5, 6). Interestingly, a report showed that vitamin intake inhibits blood homocysteine elevation even if the cause was genetic^35^. Our epidemiological study showed that respiratory function of male smokers with higher homocysteine declined more rapidly^11^. Additionally, a meta-analysis showed that higher homocysteine was thought to be a predictive marker for COPD development^15^. These results indicate that CS exposure in the hyperhomocysteinemia state more strongly enhanced ER stress-induced apoptosis than CS exposure only in alveolar epithelial cells and exacerbated emphysematous change in the lungs. Future studies are needed to reveal that vitamin intake can improve smoking susceptibility.

This study has several limitations. First, although the importance of ER stress in the alveolar cell apoptosis in vitro was shown in this study, we could not evaluate ER stress in vivo study. We performed animal experiments to examine whether smoking exposure in the context of methionine-induced hyperhomocysteinemia resulted in increased endoplasmic reticulum stress. However, there was no significant difference in the expression of endoplasmic reticulum stress protein in the whole lung tissue after 2 weeks of smoking exposure under hyperhomocysteinemia compared to the other groups (Supplemental Fig. 1). This result may be due to the fact that it was difficult to determine which cells were affected by smoking exposure in a whole lung tissue study. However, in our study, we observed that emphysema was exacerbated by smoking exposure under hyperhomocysteinemia in animal experiments, and investigated the detailed mechanism in cell experiments. In this way, we observed the phenomenon in animal experiments and investigated the detailed mechanism in cellular experiments. We believe this is important in order to clear the ethical issues of animal experiments. Second, oxidative stress and inflammation were not investigated, which are inducible factor for ER stress in this study. Finally, the regulation of thiol metabolism including homocysteine was suggested to differ between mouse and human. As regards basal status, mice have lower blood homocysteine levels than humans^36^. And in the response of oral methionine loading, glutathione increases in mice but decreases in humans^37^. Although the mechanisms underlying such differences between species remain unknown, since the antioxidant glutathione is low in humans, hyperhomocysteinemia may be more harmful in humans than in mice.

In conclusion, this study demonstrated that hyperhomocysteinemia increased alveolar epithelial cell death caused by CS exposure through ER stress-induced apoptosis and enhanced lung emphysematous changes for the first time. It was suggested that there is a synergistic effect between homocysteine and CS. This study indicates that hyperhomocysteinemia is a risk factor influencing smoking susceptibility. Hyperhomocysteinemia could be the new target for the prevention and treatment of COPD.

## Materials and methods

### Animal welfare

The study protocol was approved by the Animal Subjects Committee of Yamagata University Faculty of Medicine (Approval number 29050, Date of approval March 9th, 2017, Approved by Hiroshi Iizuka (Chairman of Animal Research Committee Director (Research) Yamagata University)). All experimental procedures were performed according to the animal welfare regulations of Yamagata University Faculty of Medicine and The ARRIVE guidelines (Animal Research: Reporting of In Vivo Experiments) 2.0.

### Materials and reagents

l-Methionine, d,l-homocysteine, vitamin B12, and folate were purchased from Sigma-Aldrich (St. Louis, MO, USA). Rabbit monoclonal anti-p-eIF2α and rabbit polyclonal anti-β-Tubulin were purchased from Cell Signaling Technologies (Danvers, MA, USA). Rabbit polyclonal anti-phospho- IRE1α was purchased from Novus Biologicals (Littleton, CO, USA). Rabbit polyclonal anti-t-IRE1α, rabbit polyclonal anti-GRP78, mouse monoclonal anti-CHOP, and rabbit polyclonal anti-homocysteine were purchased from Abcam (Cambridge, MA, USA). Annexin V-FITC Kit was purchased from MBL (Nagoya, Japan).

### Animal maintenance and diet-induced hyperhomocysteinemia mice model

Seven- to nine-week-old male C57BL/6 mice were housed in standard cages (2–5 per cage) in a temperature-controlled room with a 12/12-h light/dark cycle and were fed a normal diet (Oriental Yeast Co., Ltd, Tokyo, Japan). A report showed that methionine supplementation at concentrations of 1% in water induced hyperhomocysteinemia in male C57BL/6 mice^38^. The mice were administered to normal water or l-methionine (Met) at 1% concentrations in drinking water and were exposed to the air or CS of five nonfiltered cigarettes (Peace; Japan Tobacco Inc., Japan) for 30 min, twice per day, 5 days per week, using a whole-body smoking exposure apparatus (INH03-CIGR01A; MIPS, Osaka, Japan). For sample size determination, we determine a reasonable number of animals that can be statistically tested, do not involve excessive animal sacrifice, and refer to similar experimental reports in the previous study^39^. All mice that had completed drug administration or smoking exposure were included in the analysis.

### Measurement of plasma homocysteine

Blood was collected from the inferior vena cava to the tubes containing K2 EDTA. Plasma was separated by centrifugation at 4 °C, 1300 × *g* for 15 min. Plasma homocysteine level was measured using high-performance liquid chromatography^40^, with the cooperation of the Japan Institute for the Control of Aging, NIKKEN SEIL Co., Ltd.

### Histological analysis

To examine the development of pulmonary emphysema, the lungs were fixed with intratracheal instillation with 4% buffered formalin at a constant pressure of 25 cmH_2_O, and paraffin-embedded lung blocks were prepared. Three-µm-thick lung sections were stained with hematoxylin and eosin. The mean linear intercept (MLI), as a measure of the interalveolar septal wall distance^41^, was measured using light microscopy at 400× magnification. The MLI was obtained by dividing the length of a line drawn across the lung section by the total number of intercepts encountered in 50 lines per mouse lung, as described previously^39^.

DI was analyzed with 50 equally spaced points per field on a transparent sheet. Referring to previous reports, destruction was assessed when the tissue on the points met one or more of the following criteria: (a) at least two defects in the alveolar wall, (b) at least two ruptures in the luminal parenchyma of the alveolar ducts, (c) obvious morphological abnormalities, and (d) classical emphysematous lesions^42^. DI was calculated by dividing the number of destroyed alveoli by the total number of alveoli underlying the points. MLI and DI was determined by evaluating 10 fields of view per an individual lung tissue.

### Cell culture

Human lung alveolar epithelial A549 cells were purchased from the Cell Resource Center for Biomedical Research, Institute of Development, Aging and Cancer, Tohoku University, Japan, and were cultured in DMEM supplemented with 10% fetal calf serum, L-glutamine, and an antibiotic–antimycotic solution (100 units/ml of penicillin, 100 μg/ml of streptomycin, and 0.025 μg/ml of amphotericin B; Gibco) in a humidified incubator at 37 °C with 5% CO_2_. To examine the cytotoxic effects of homocysteine and cigarette smoke extract (CSE), A549 cells were stimulated using varying concentrations of homocysteine (0‒10 mM) and/or 20% of CSE. The concentration of homocysteine used in our study was adjusted based on the concentration used in previous studies^16,17^.

### Preparation of cigarette smoke extract

CSE was produced as described previously^34,43^. Briefly, smoke from one stick of cigarette was bubbled through 25 ml of Hanks’ Balanced Salt Solution. The resultant product was defined as 100% CSE.

### Vitamin treatment

In the previous study, vitamin B12 and folate supplementations have been reported to reduce ER stress caused by homocysteine^17^. Vitamin B12 were dissolved in water. Folate was dissolved in 0.1 M NaOH. Compounds with the same vitamin B12 and folate concentration ranging 5‒50 μM were diluted in a cultured medium to examine whether these compounds can restore ER stress in a dose-dependent manner.

### Flow cytometry

A549 cell apoptosis was investigated following the addition of fluorescein isothiocyanate (FITC)-conjugated Annexin V and propidium iodide according to the manufacturer’s protocol. Apoptosis was regarded as the cell percentage in the Q3 quadrant. The results were analyzed using flow cytometry (EC800 Flow Cytometry Analyzer; Sony Biotechnology Inc. Tokyo, Japan).

### Western blotting analysis

A549 cells were lysed in ice-cold radioimmunoprecipitation assay lysis buffer containing 50 mM Tris–HCl, pH 7.5, 150 mM NaCl, 1 mM EDTA, 0.1% NP-40, 1 mM DTT, 0.1% SDS, 100 mM PMSF, 100 mM NEM, 100 mM iodoacetamide, and 1% phosphatase inhibitor. The protein concentration of each sample was determined using the BCA protein assay (Bio-Rad Laboratories, Inc., Hercules, CA, USA). Equal amounts of protein were electrophoresed on 8–10% sodium dodecyl sulfate–polyacrylamide gels and electrotransferred onto polyvinylidene difluoride membranes (GE Healthcare UK Ltd, Little Chalfont, UK). Membranes were blocked with 20 mM Tris–HCl, pH 7.4, containing 150 mM NaCl, 0.1% Tween (TBS-T), and 5% milk or 5% BSA at room temperature for 1 h. Then the membranes were probed with primary antibodies diluted in TBS-T. After incubation with horseradish peroxidase-conjugated secondary antibodies diluted in TBS-T containing 5% milk or 5% BSA, immunoreactive bands were detected using an ECL kit (Amersham Biosciences, Piscataway, NJ, USA)^44^. The expression levels of GRP78, IRE1α, p-eIF2α and CHOP were examined. GRP78 (also known as BiP and HSPA5) is sequestered by binding to unfolded or misfolded polypeptide chains and/or unassembled multisubunit proteins, thereby leading to the release and, consequently, the activation of the ER-stress sensors such as IRE1α and PERK^45^. Each sensor introduces its own unfolded protein response signaling pathways, activated PERK p-eIF2α, causing apoptosis cascade by upregulating CHOP transcription in chronic ER stress^46^. The expression of CHOP, a pro-apoptotic protein, is currently used as a marker of chronic ER stress and subsequent apoptosis^47^.

### Statistical analysis

Data are expressed as the mean ± standard error or median and interquartile range. Differences between two groups were assessed using t-test. For analyses between groups of three or more, Tukey–Kramer test or Bonferroni/Dunn test was used. Significance was inferred for P values < 0.05. Statistical analyses were performed using JMP version 12.2 software (SAS Institute, Cary, NC, USA).

## Supplementary Information

Supplementary Figures.
