# Supplementary material for: Effect of hyperhomocysteinemia on a murine model of smoke-induced pulmonary emphysema
Source: Sci Rep. 2022 Jul 28;12:12968. doi: 10.1038/s41598-022-16767-2 (PMC9334265; doi:10.1038/s41598-022-16767-2)
Supplement: Supplementary file 1 — Supplementary Figures. [file 41598_2022_16767_MOESM1_ESM.docx]

Effect of hyperhomocysteinemia on a murine model of smoke-induced pulmonary emphysema.

Hiroshi Nakano, Sumito Inoue, Yukihiro Minegishi, Akira Igarashi, Yoshikane Tokairin, Keiko Yamauchi, Tomomi Kimura, Michiko Nishiwaki, Takako Nemoto, Yoichiro Otaki, Masamichi Sato, Kento Sato, Hiroyoshi Machida, Sujeong Yang, Hiroaki Murano, Masafumi Watanabe, and Yoko Shibata.

Supplemental file

Supplemental figure 1.

A. Graphic depictions of western blotting of CHOP.


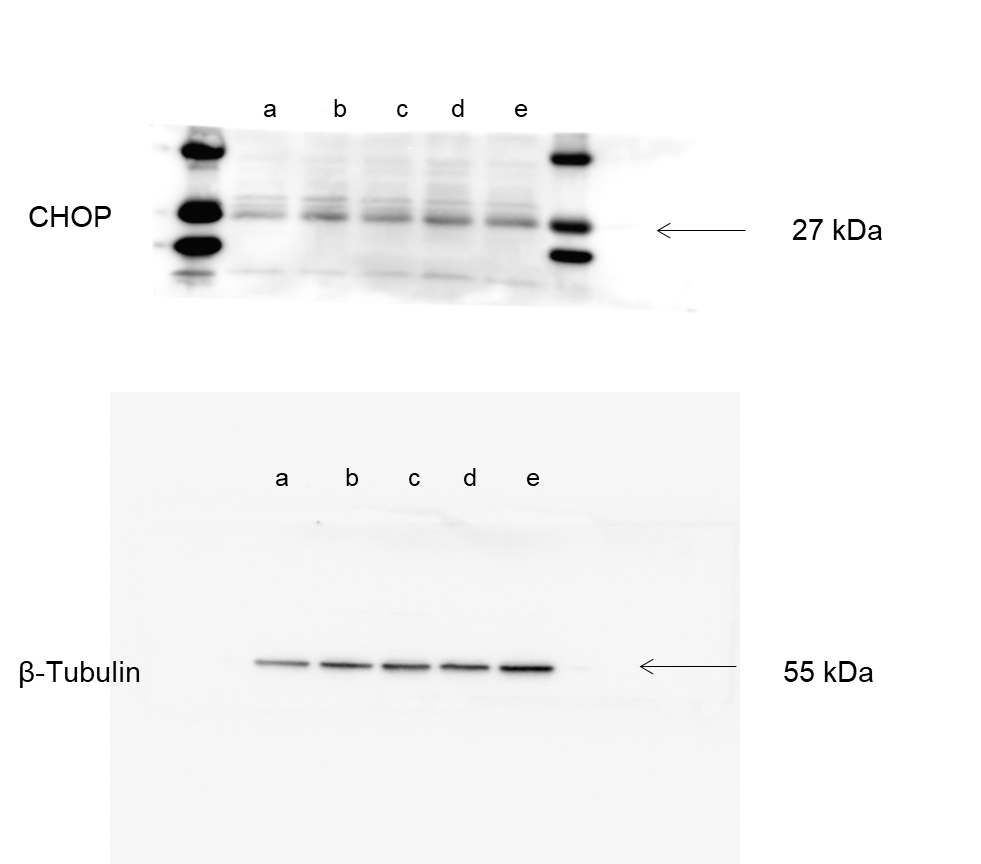


a Met -, CS -

b Met -, CS -

c Met +, CS-

d Met-, CS+

e Met+, CS+

Met; methionine, CS; cigarette smoking.

B. Protein expression levels of CHOP in the whole lung of mice.


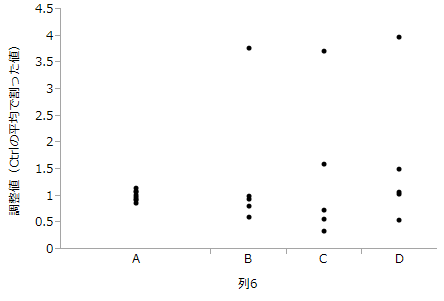


CHOP/β-Tubulin ratio

　Met / CS - / - + / - - / + + / +

Mice were treated with methionine and exposed to smoking for 2 weeks, and proteins were extracted from whole lungs and examined for CHOP expression by Western blotting. The CHOP protein expression level in each group was examined as a ratio of the Met-/CS- protein expression level to 1. In the Met-/CS- group, two samples each were measured to determine the average protein expression level. Met; methionine, CS; cigarette smoking (n=5 in each groups).

Supplemental figure 2.

Photographs of the Western blot for Figure 4.

A, GRP78


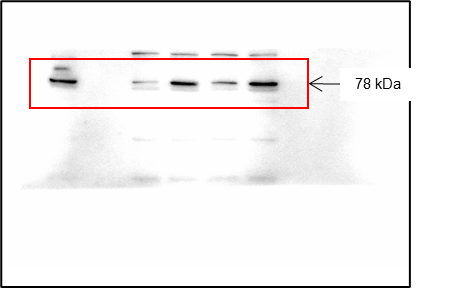


B, p-IRE1α


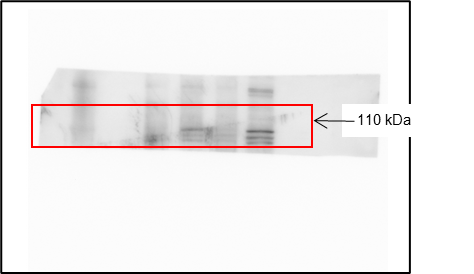


C, t-IRE1α


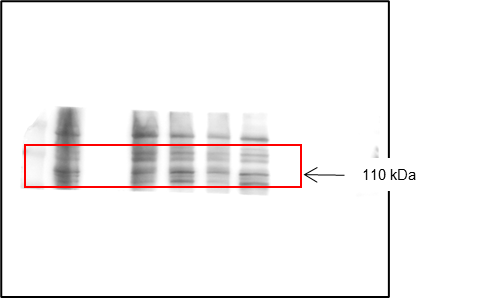


D, p-eIF2α


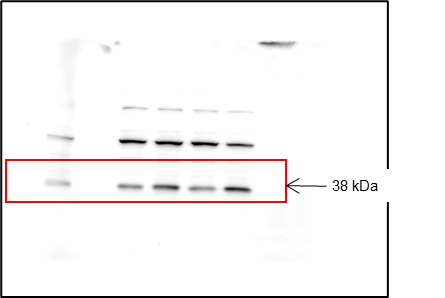


E, CHOP


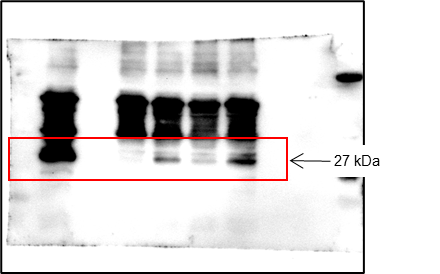


F, Tubulin


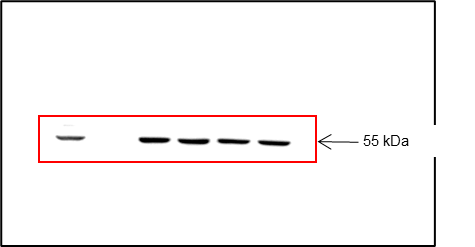


Supplemental figure 3.

Photographs of the Western blot for Figure 5.

A, Homocysteine


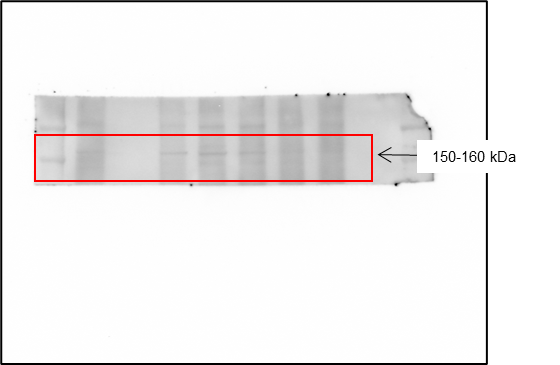


B, GRP78


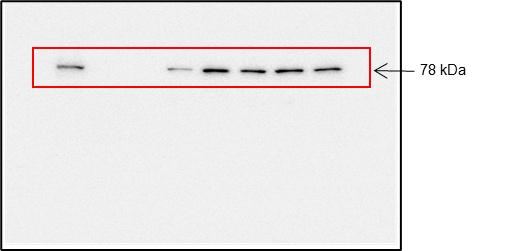


C, p-IRE1α


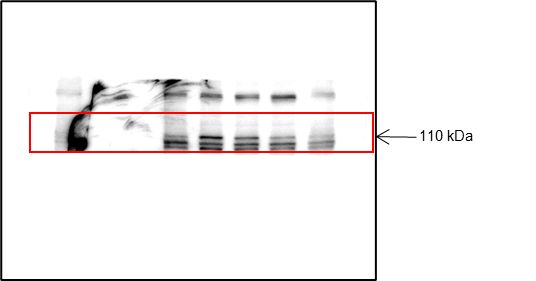


D, t-IRE1α


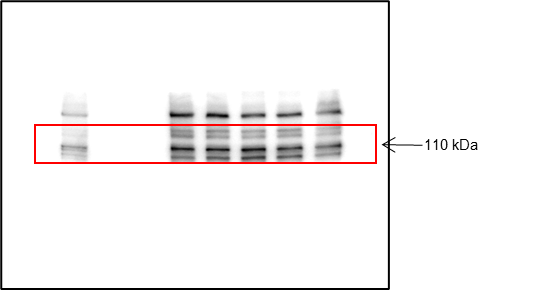


E, p-eIF2α


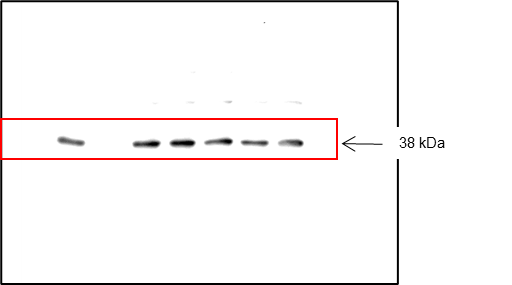


G, CHOP


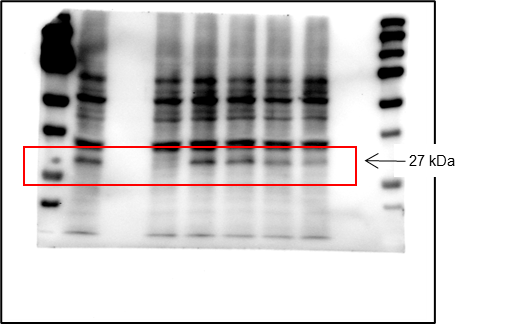


H, Tubulin


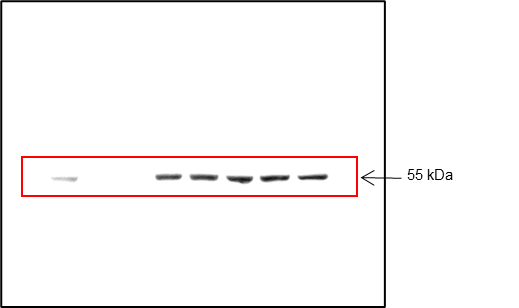


Supplemental figure 4.

All membranes for western blotting for Figure 4.

a; control, b; homocysteine 5 mM, c; 20% CSE, d; homocysteine 5 mM + 20% CSE

GRP78


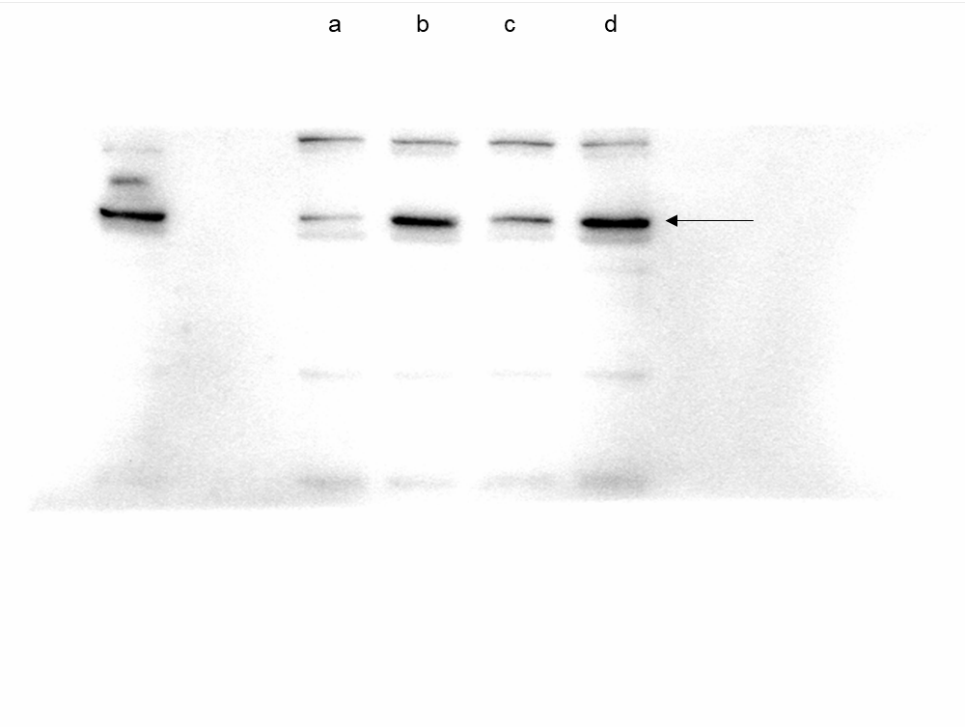


Tubulin


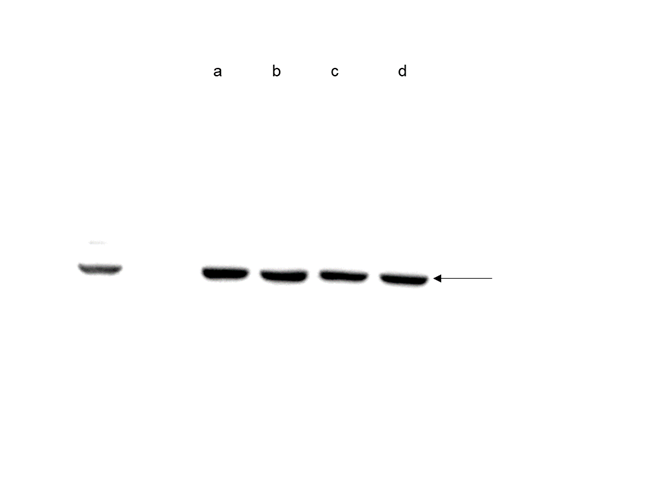


GRP78


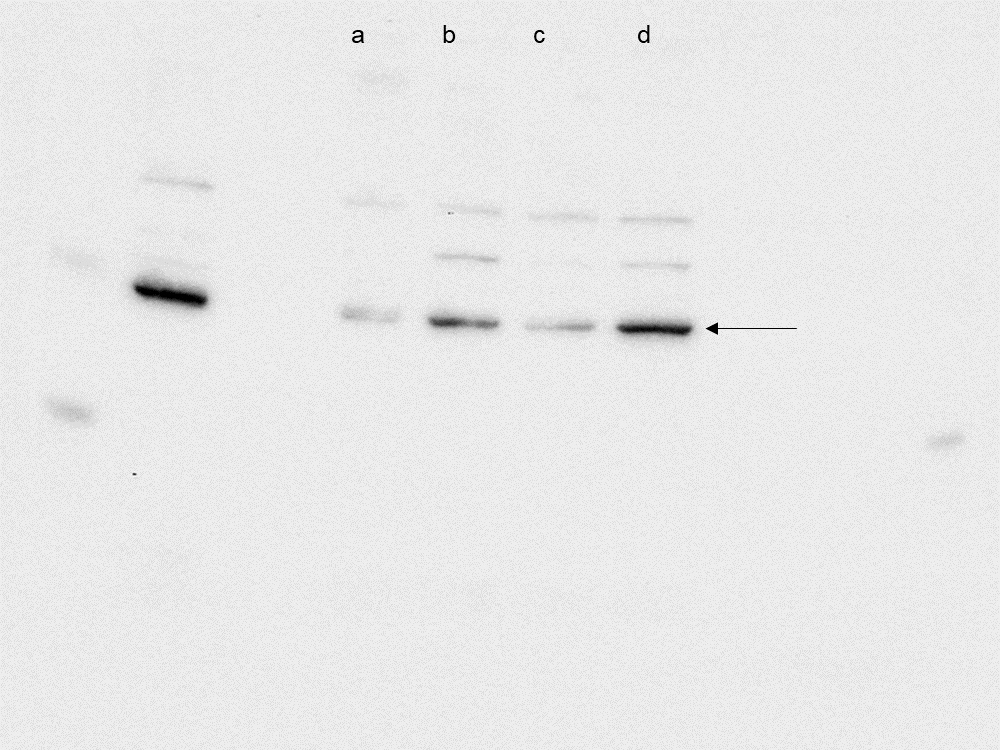


Tubulin


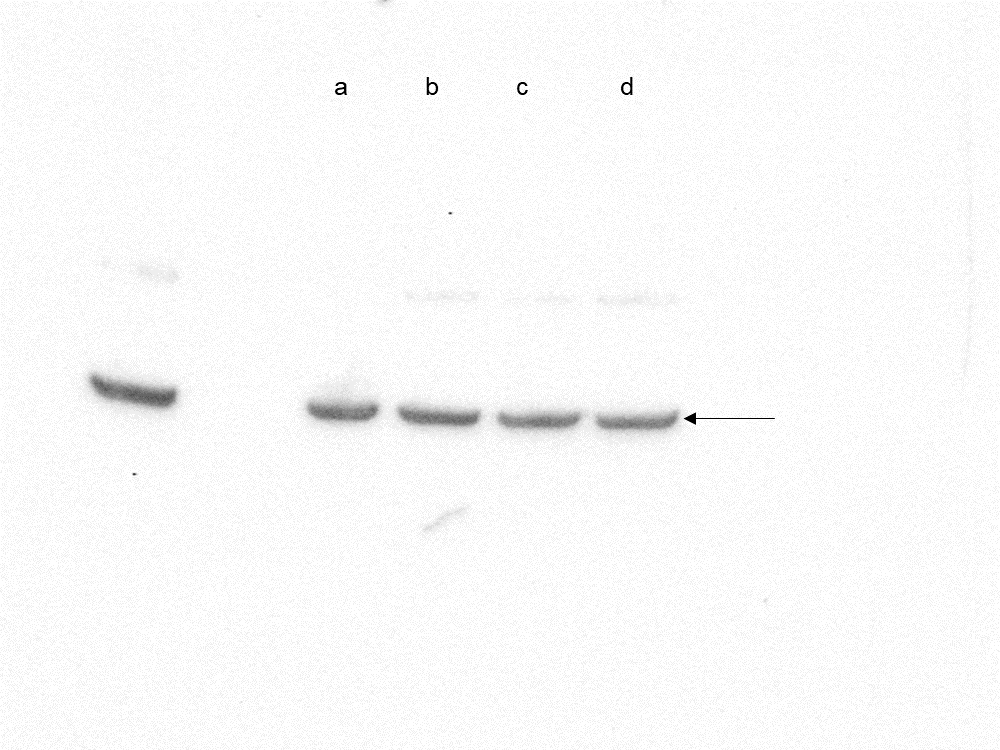


GRP78


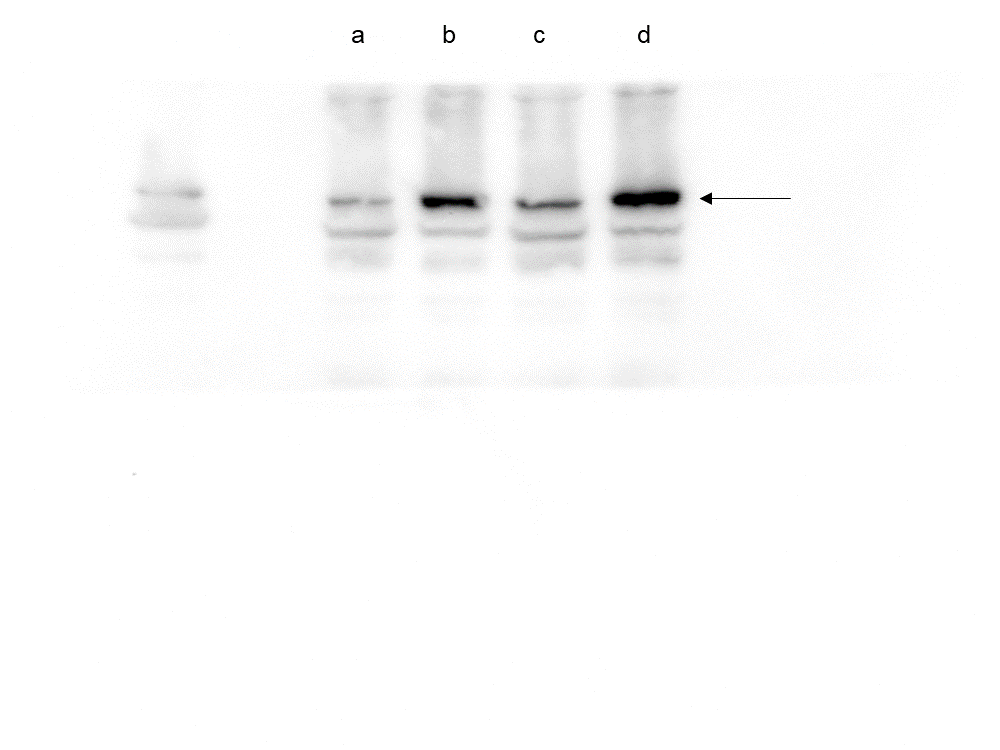


Tubulin


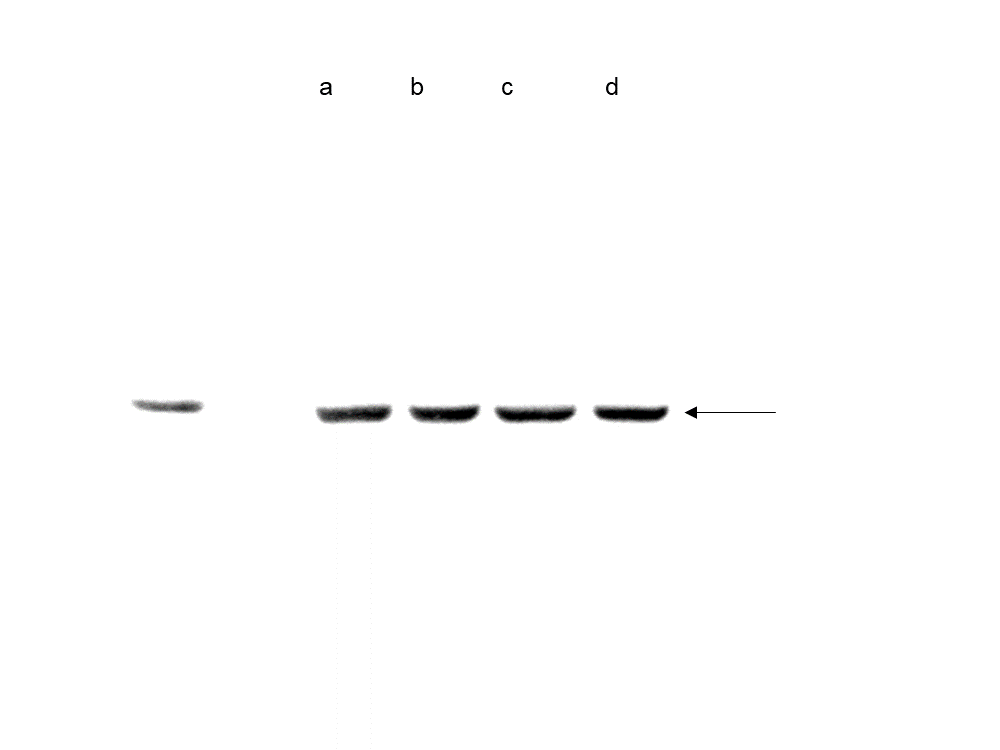


GRP78


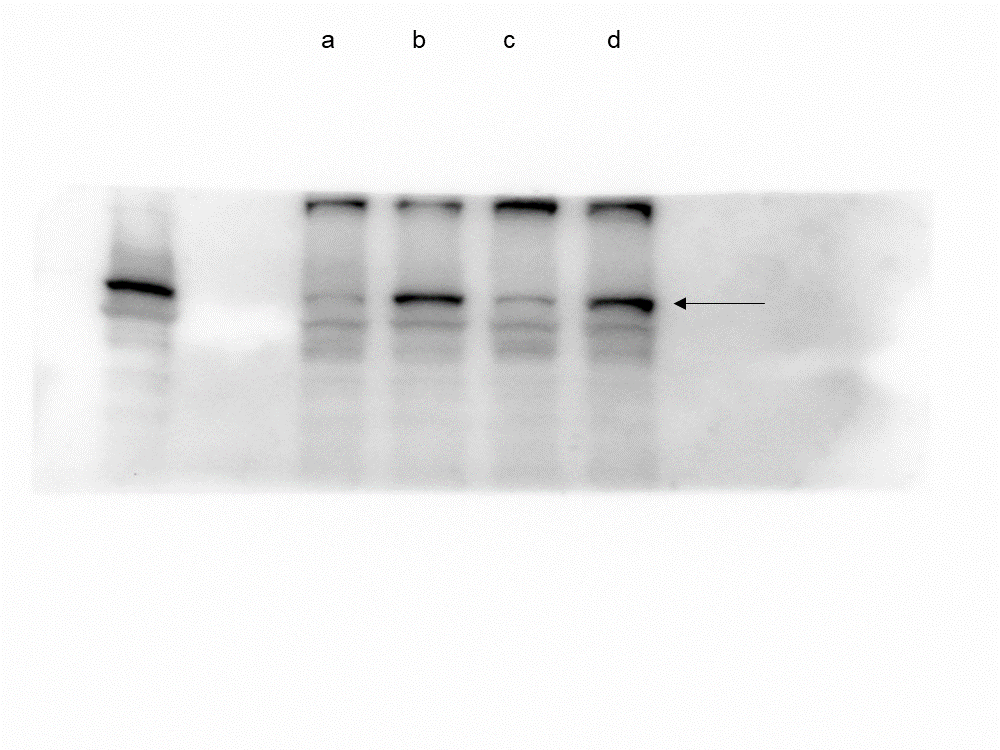


Tubulin


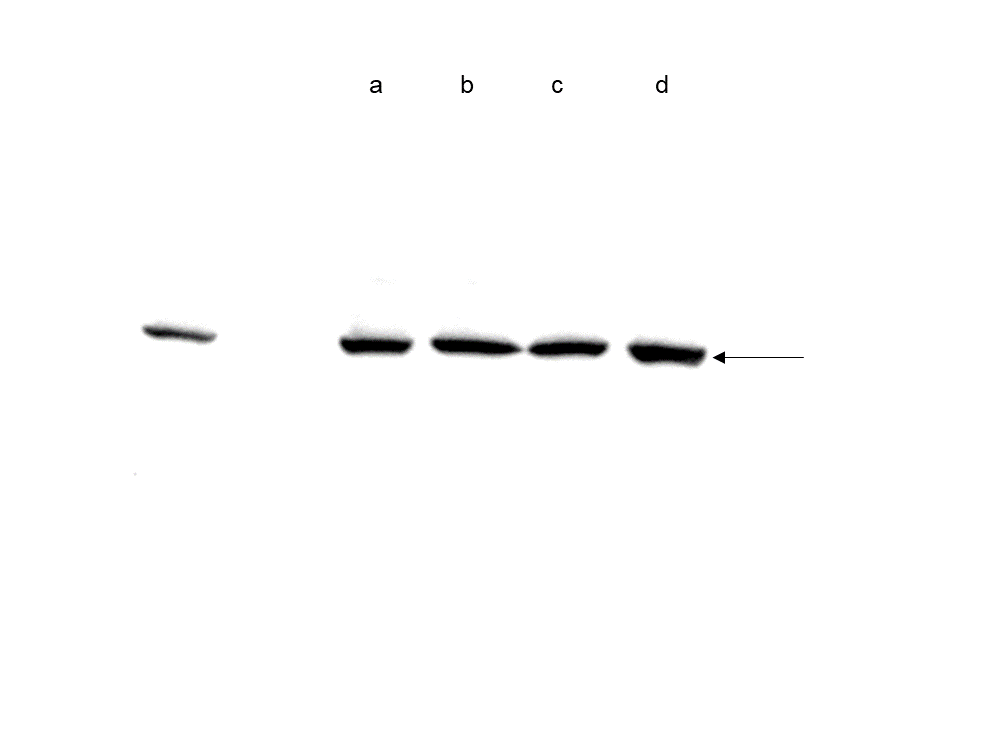


GRP78


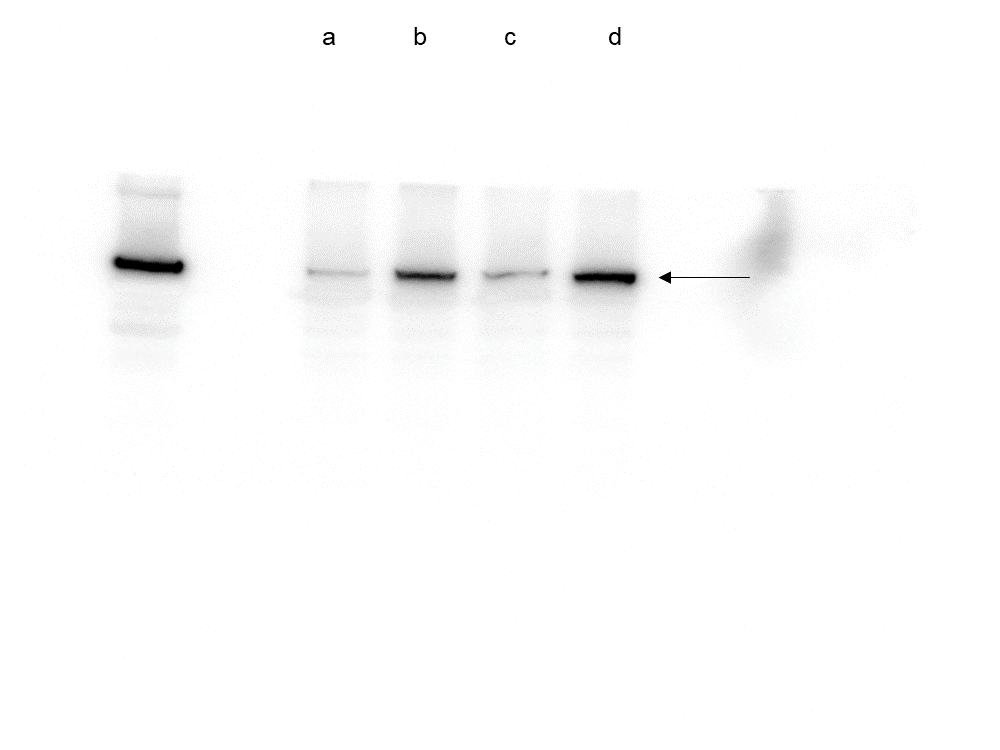


Tubulin


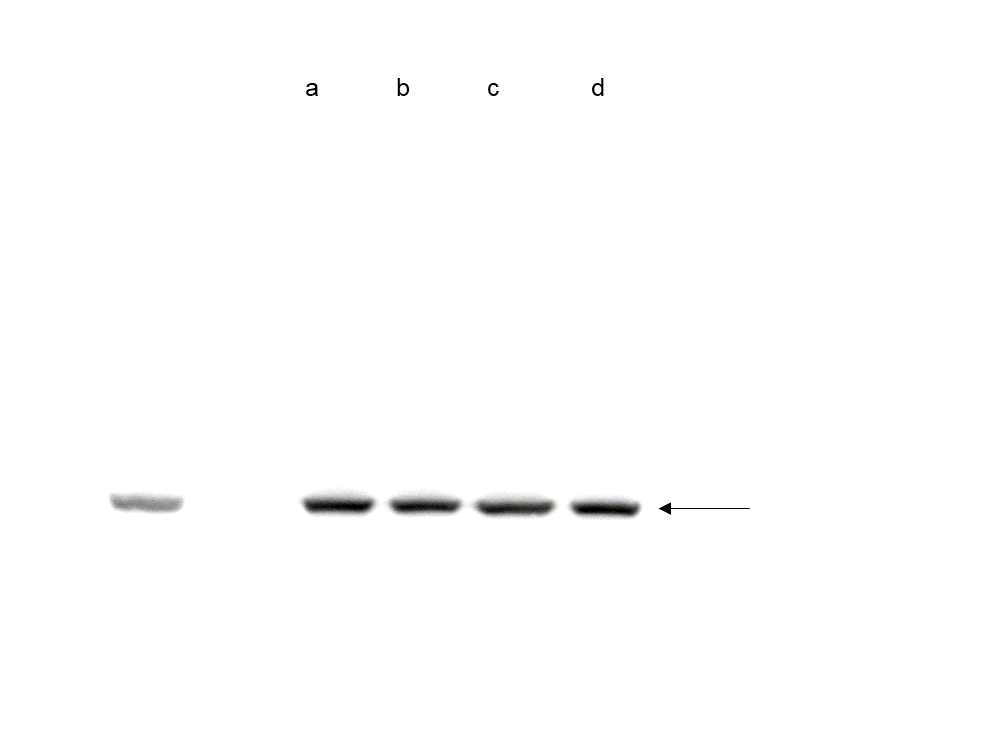


pIRE1


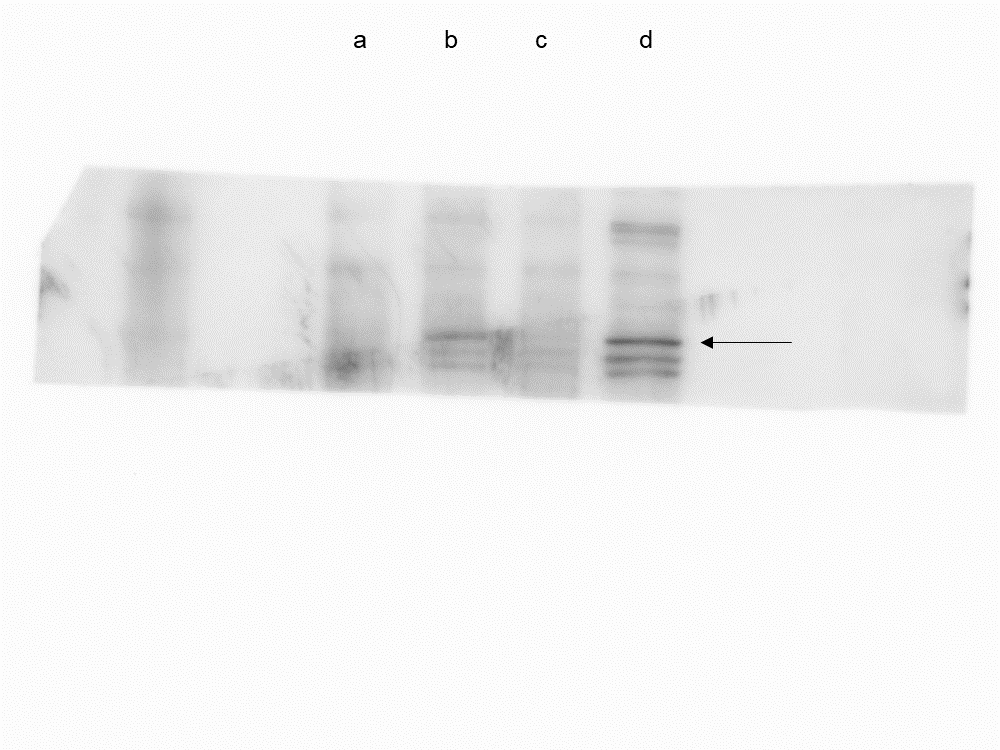


tIRE1


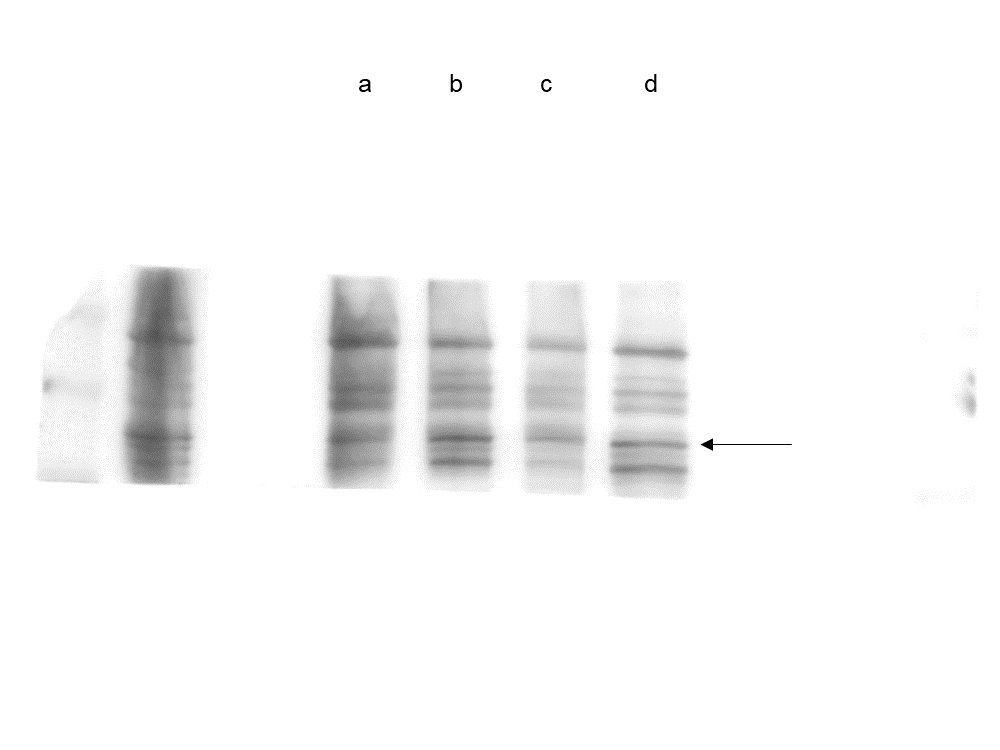


pIRE1


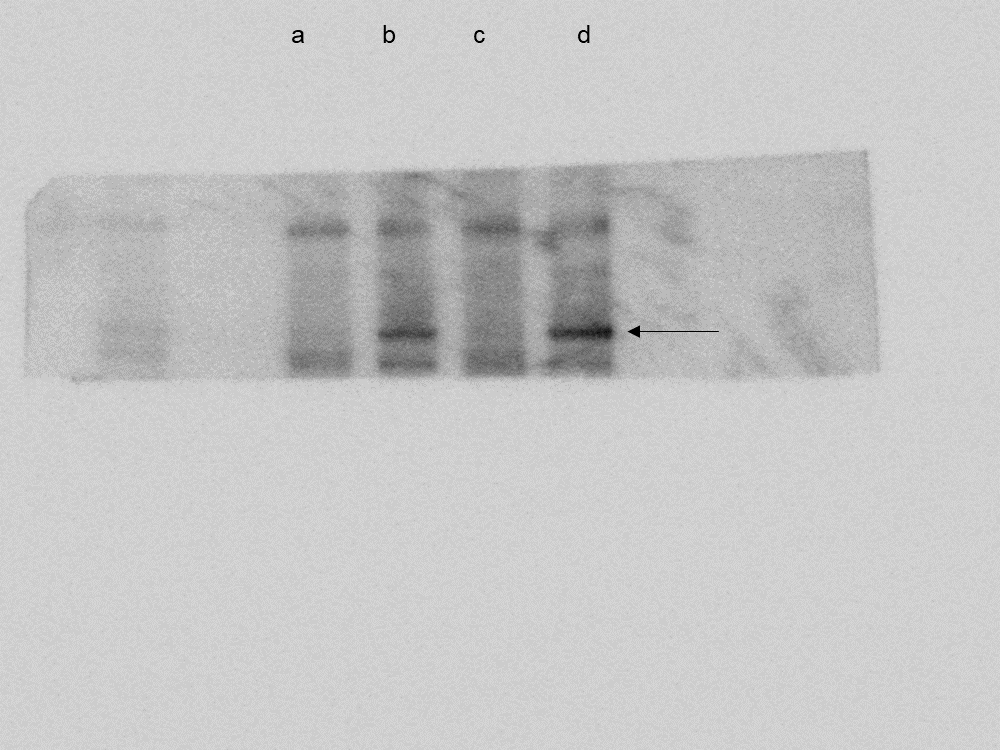


tIRE1


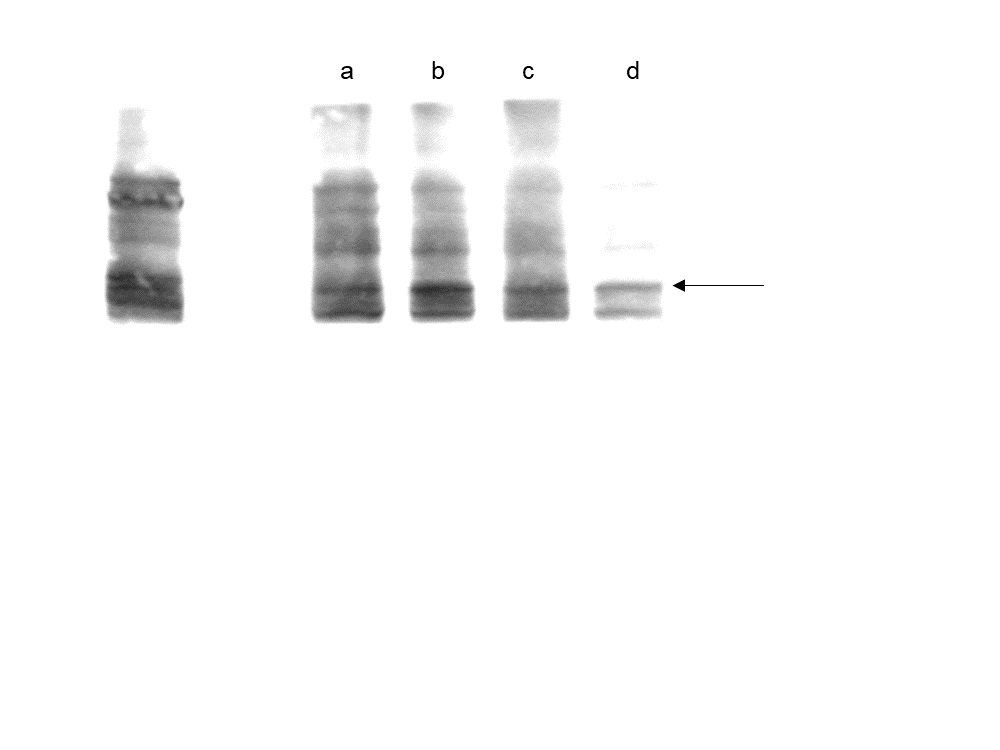


pIRE1


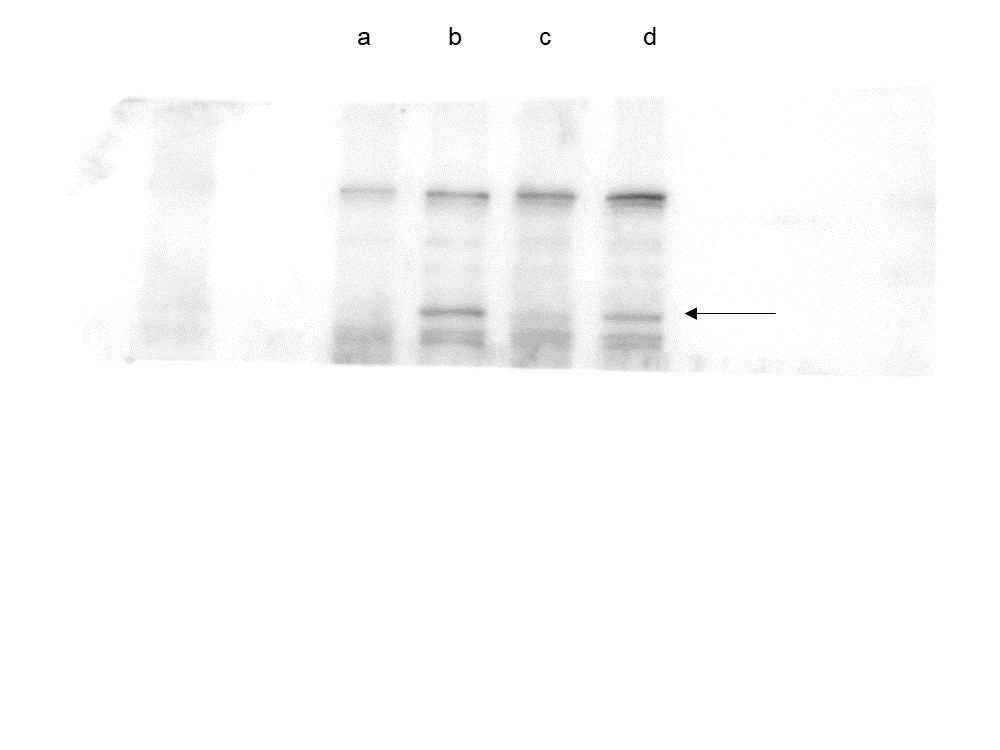


tIRE1


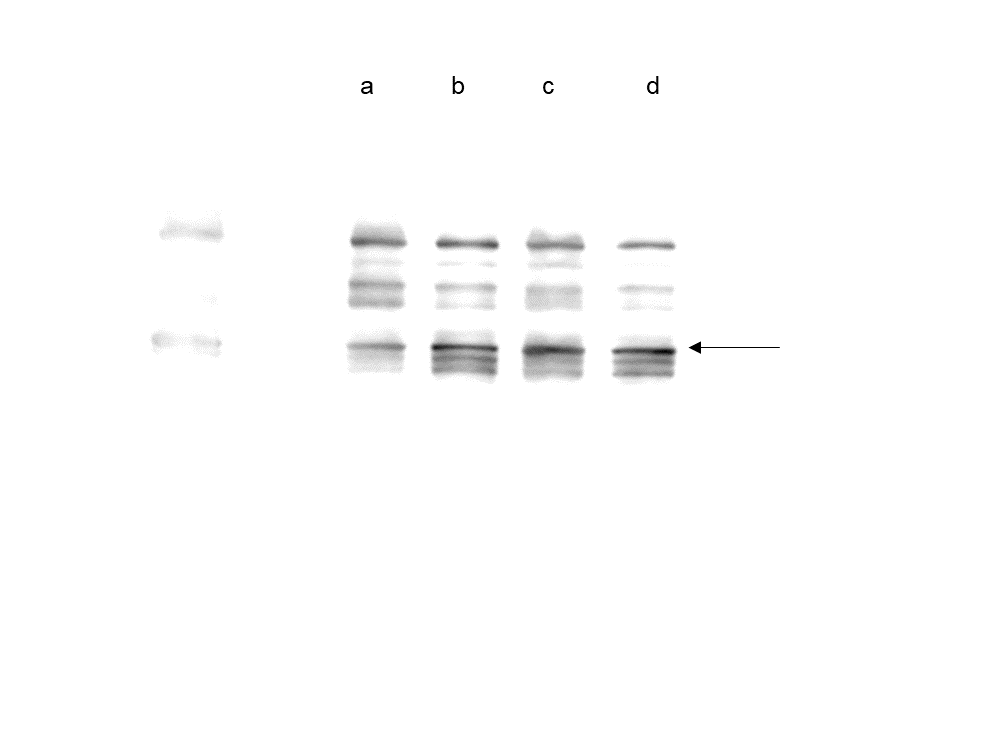


pIRE1


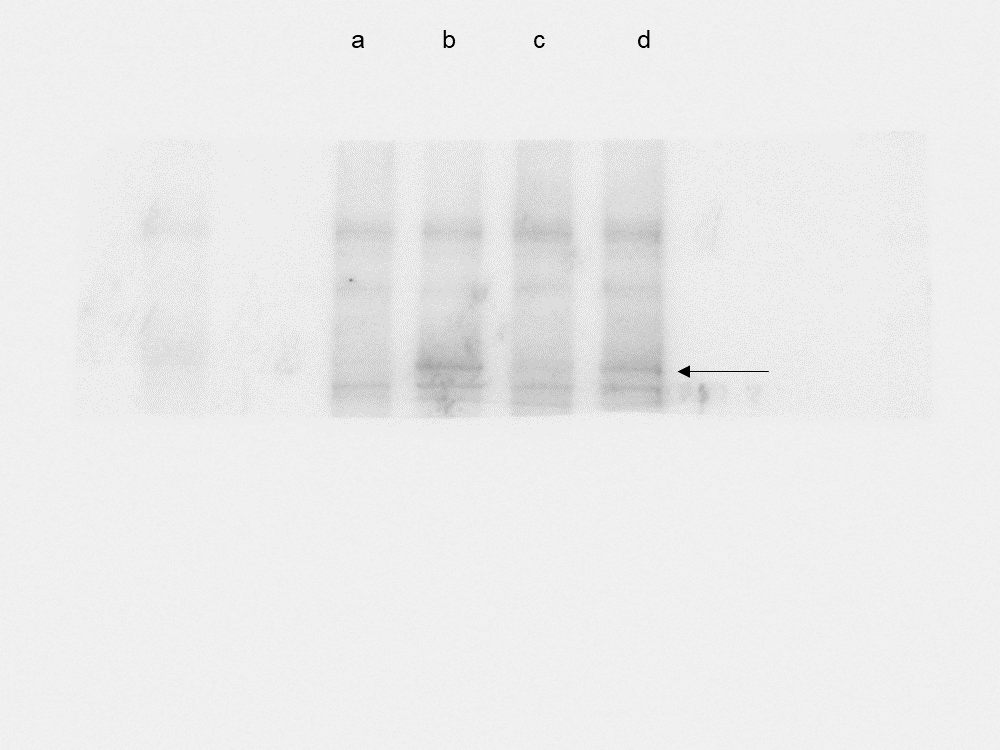


tIRE1


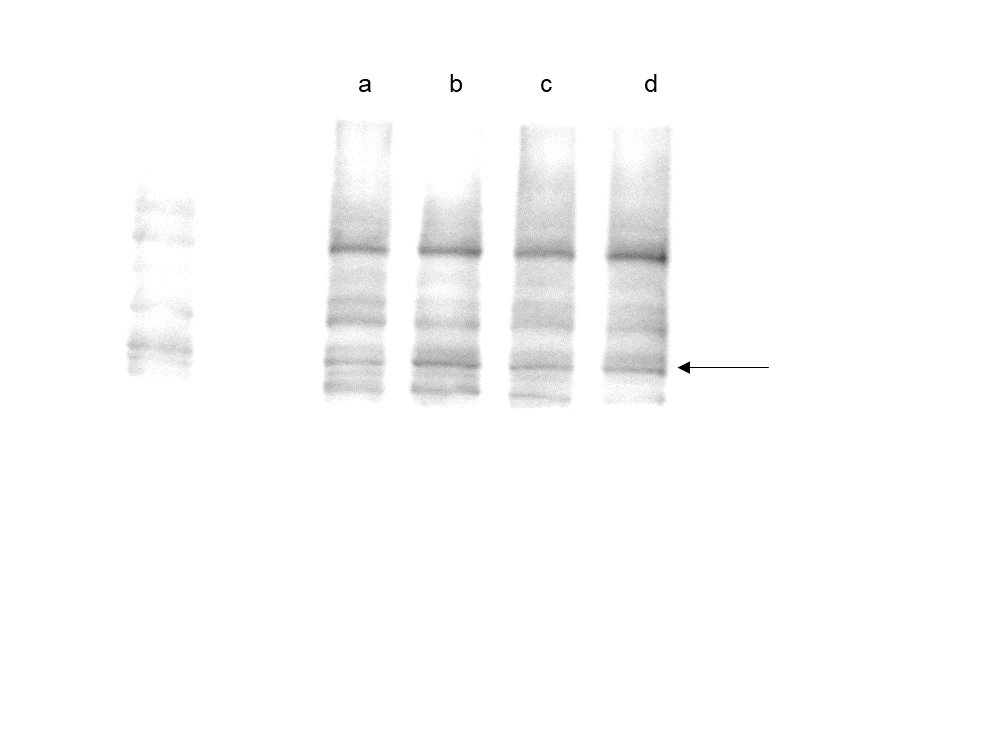


pIRE1


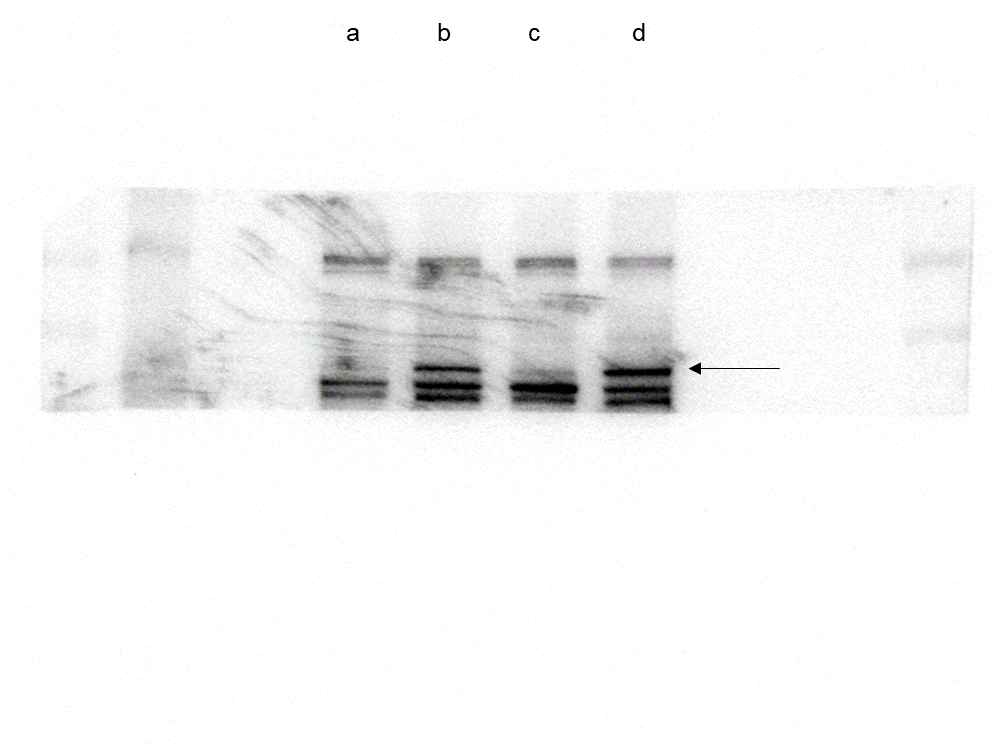


tIRE1


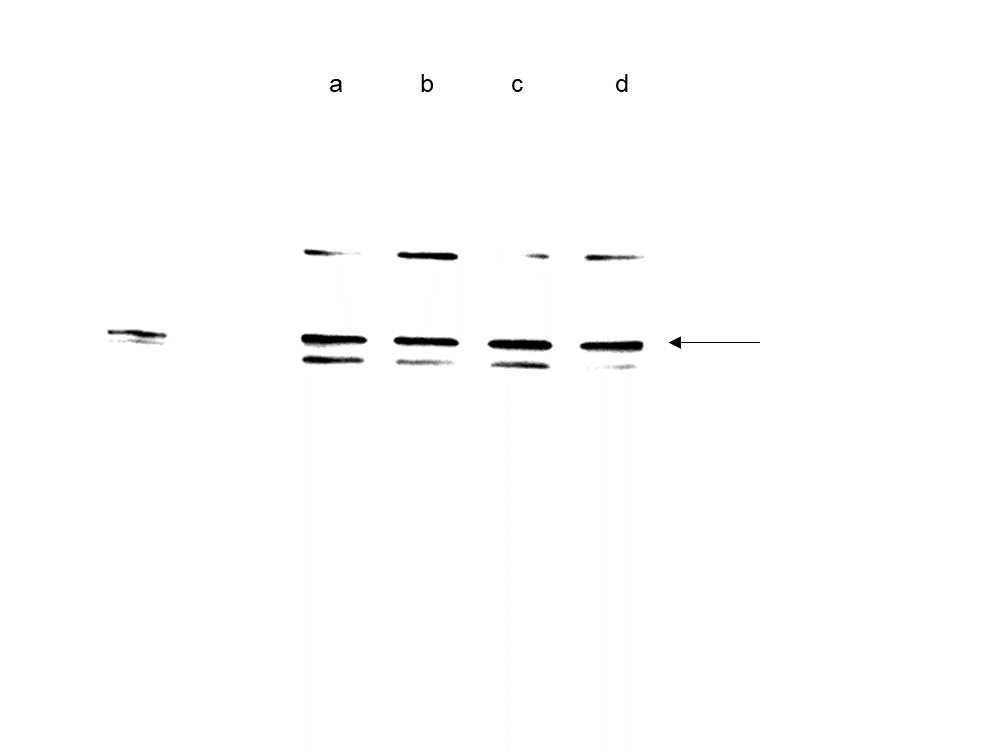


p-eIF2α


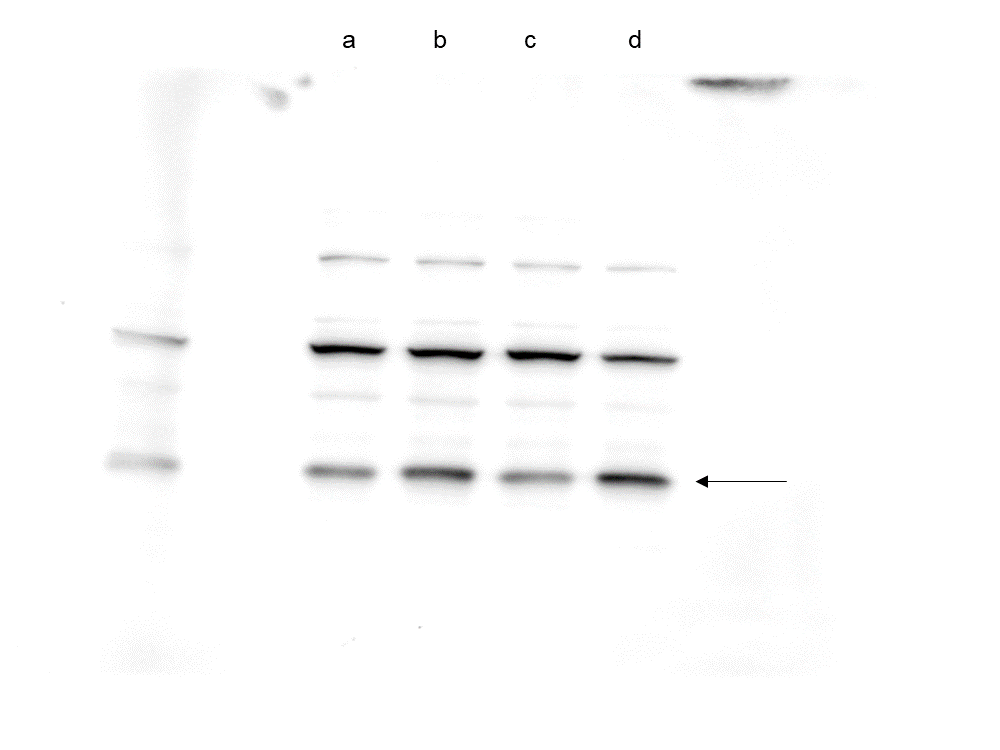


Tubulin


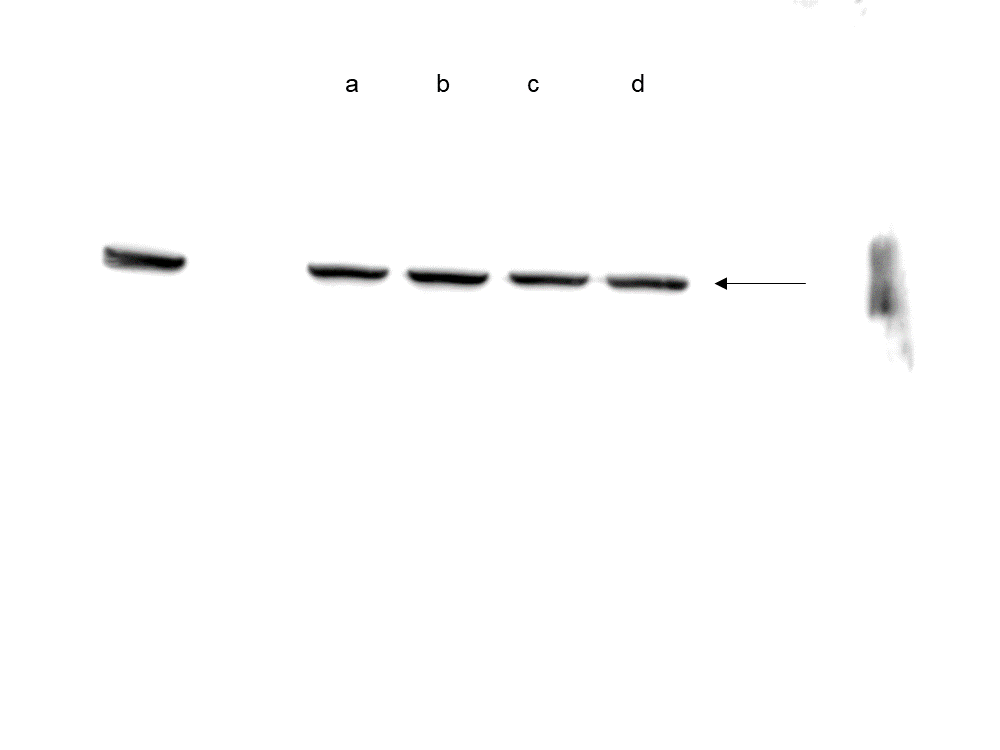


p-eIF2α


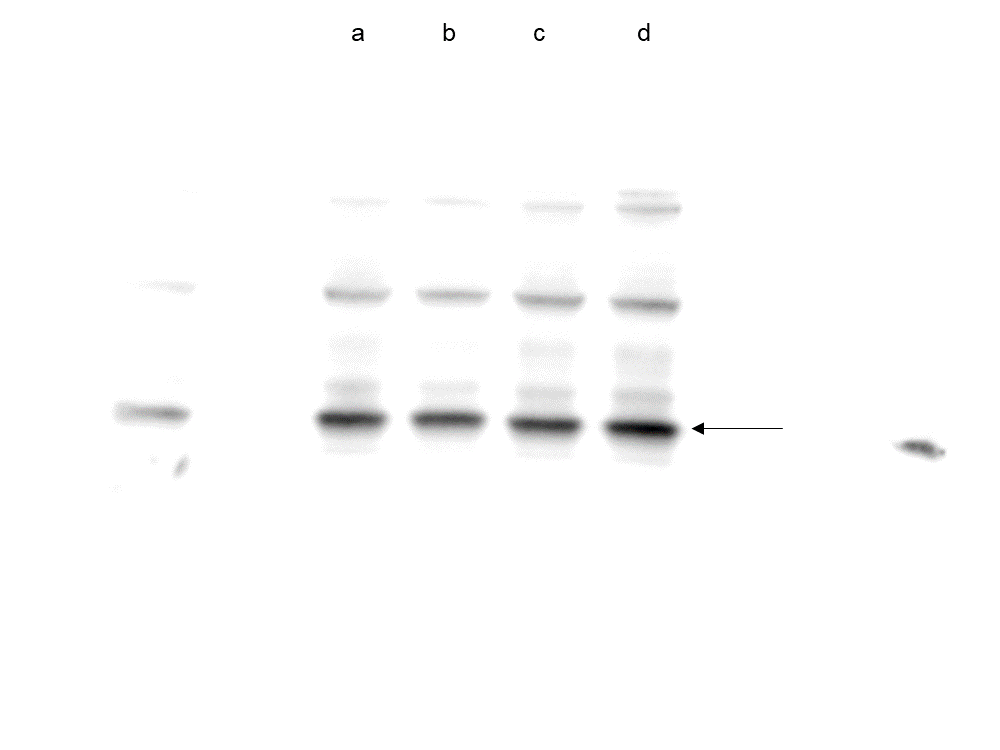


Tubulin


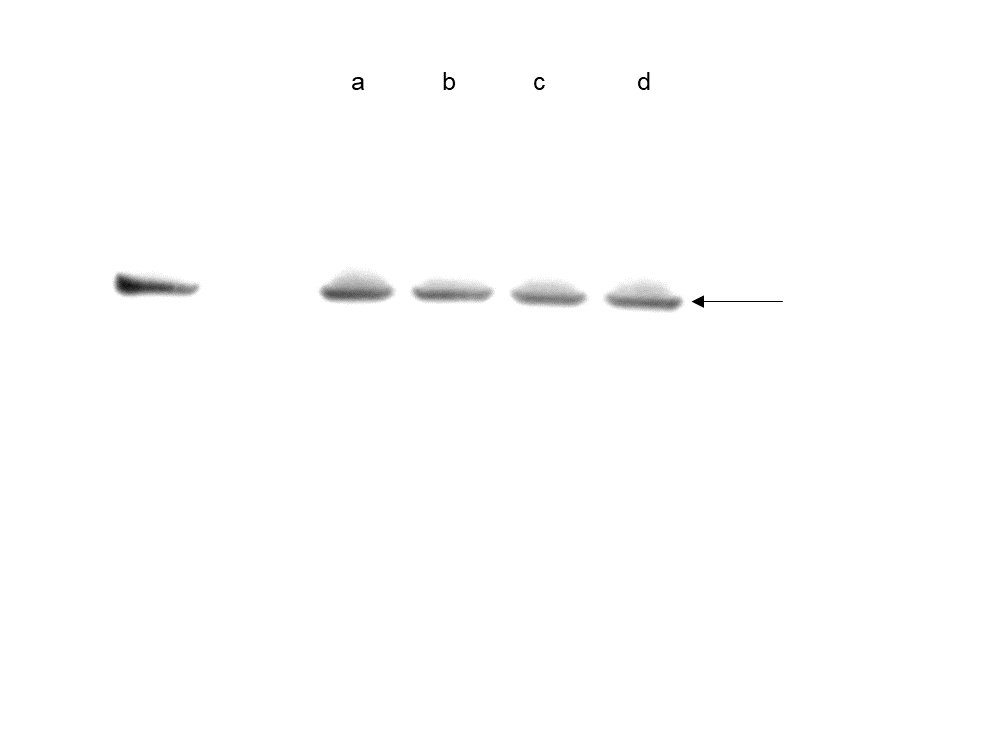


p-eIF2α


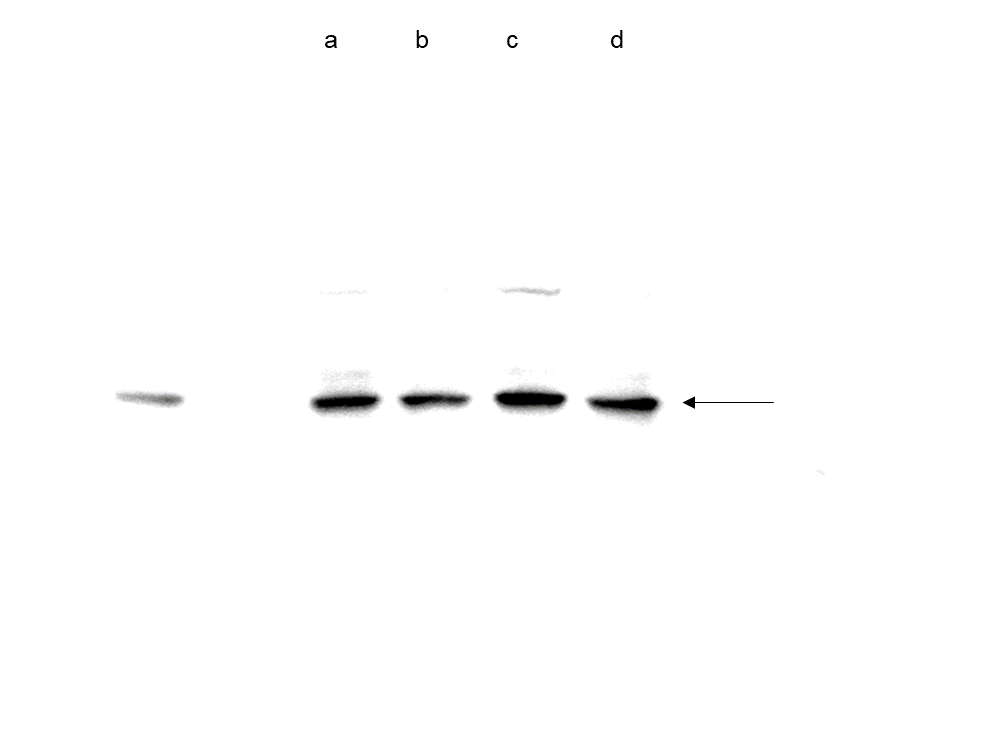


Tubulin


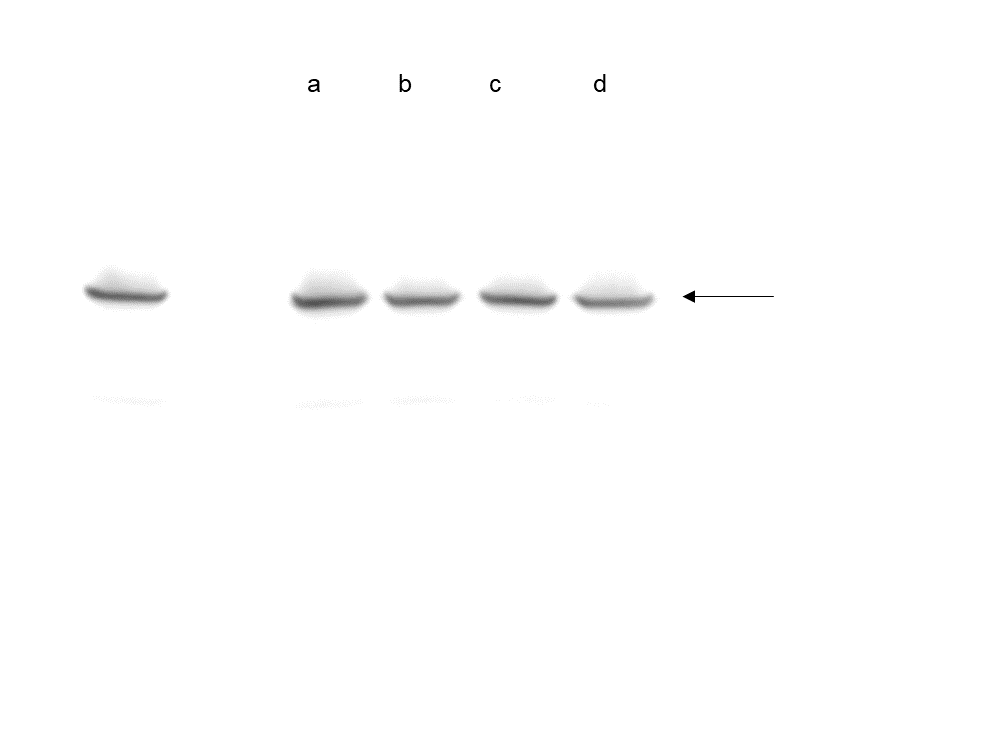


p-eIF2α


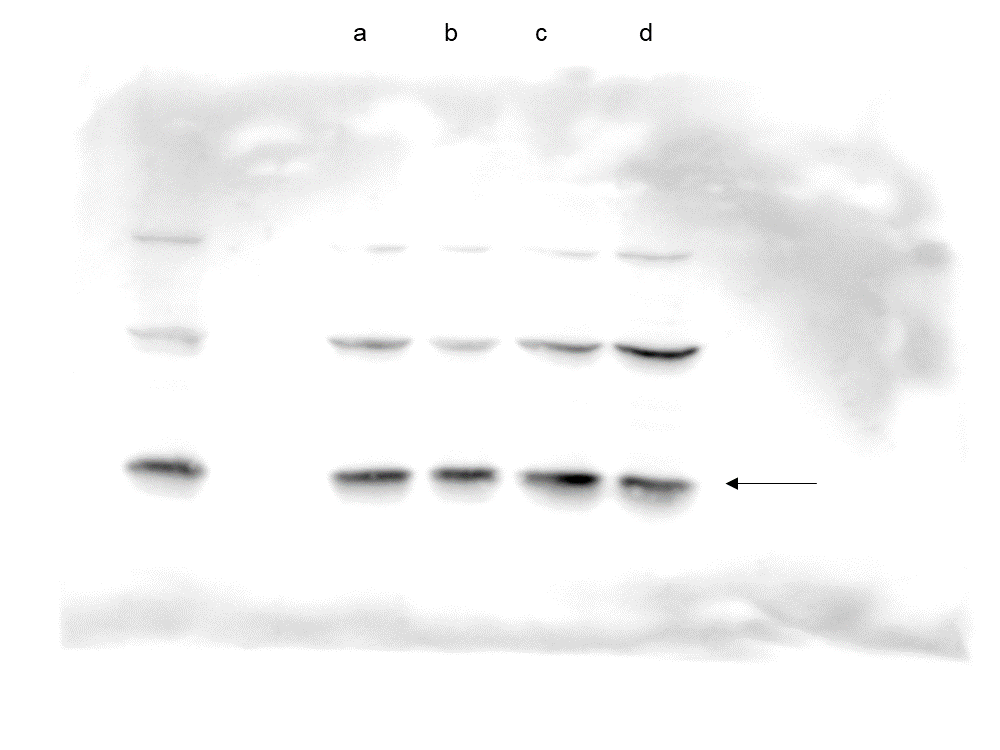


Tubulin


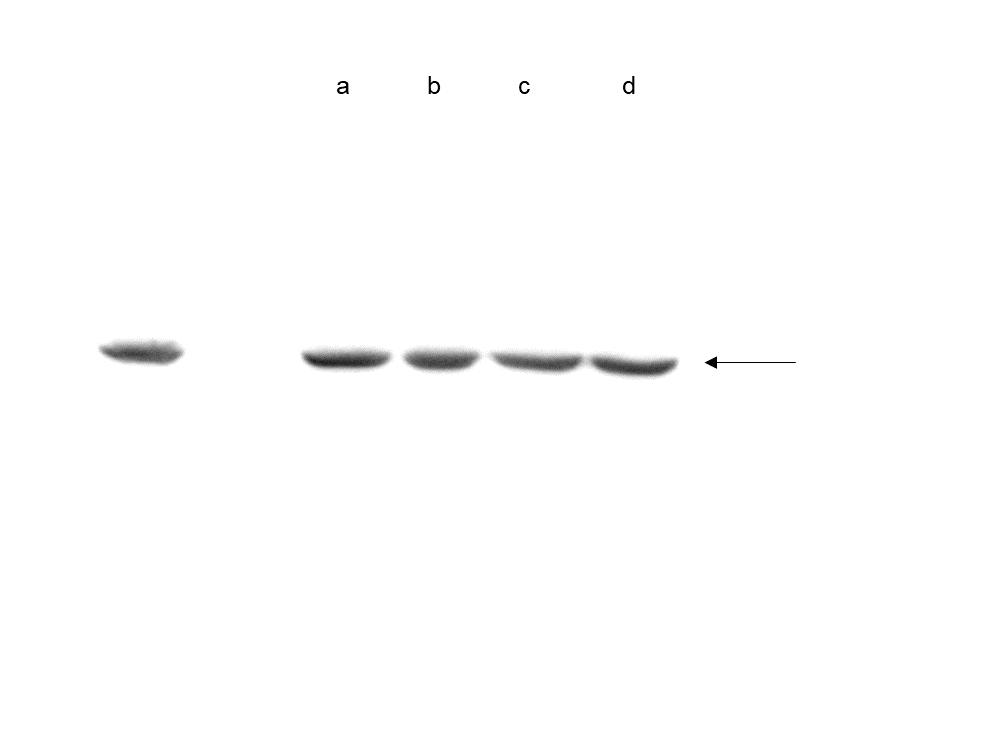


p-eIF2α


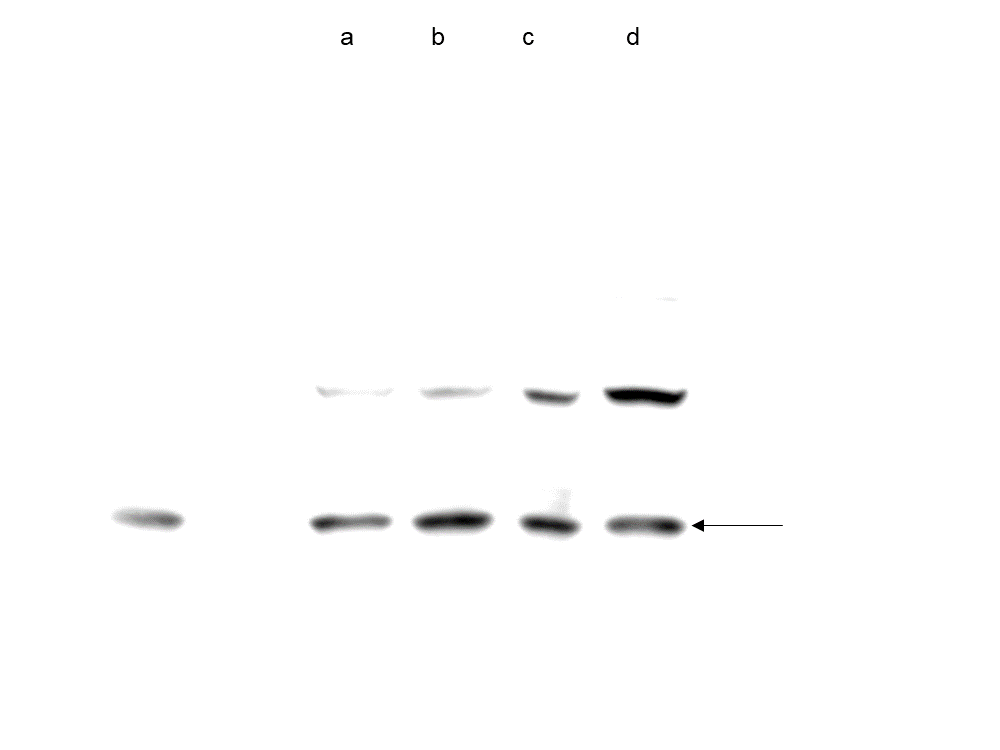


Tubulin


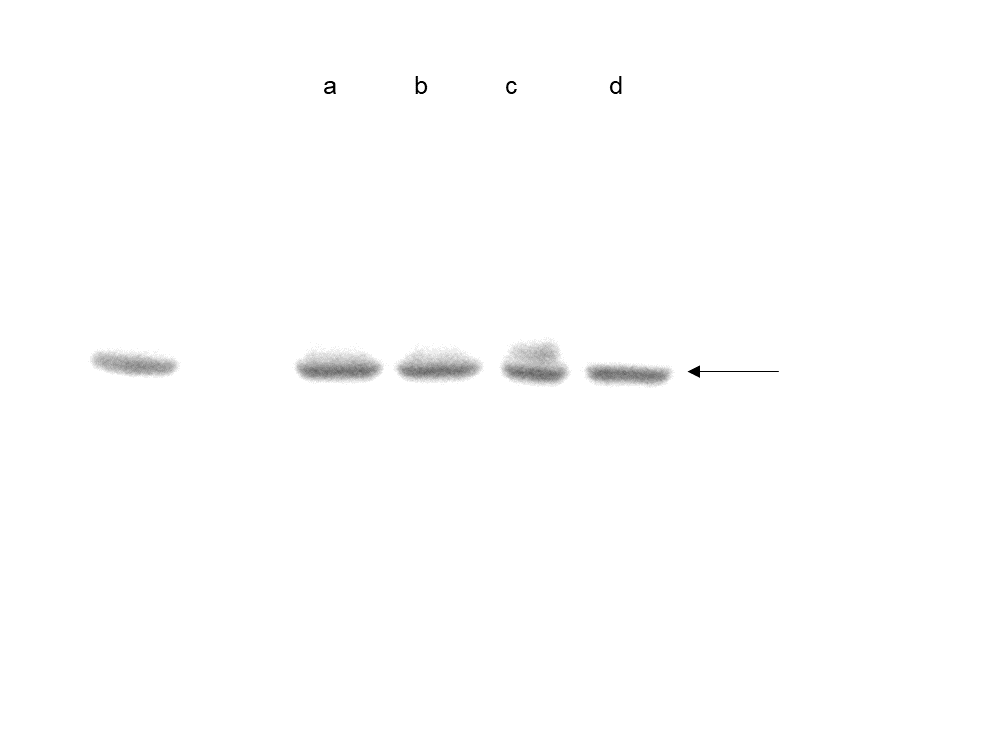


CHOP


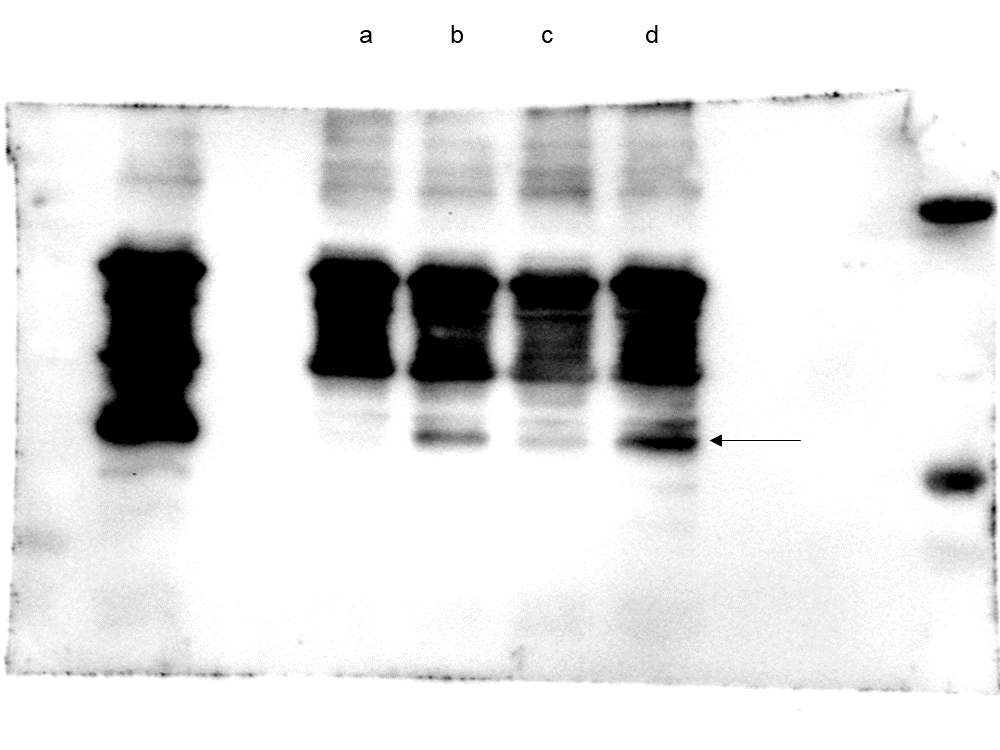


Tubulin


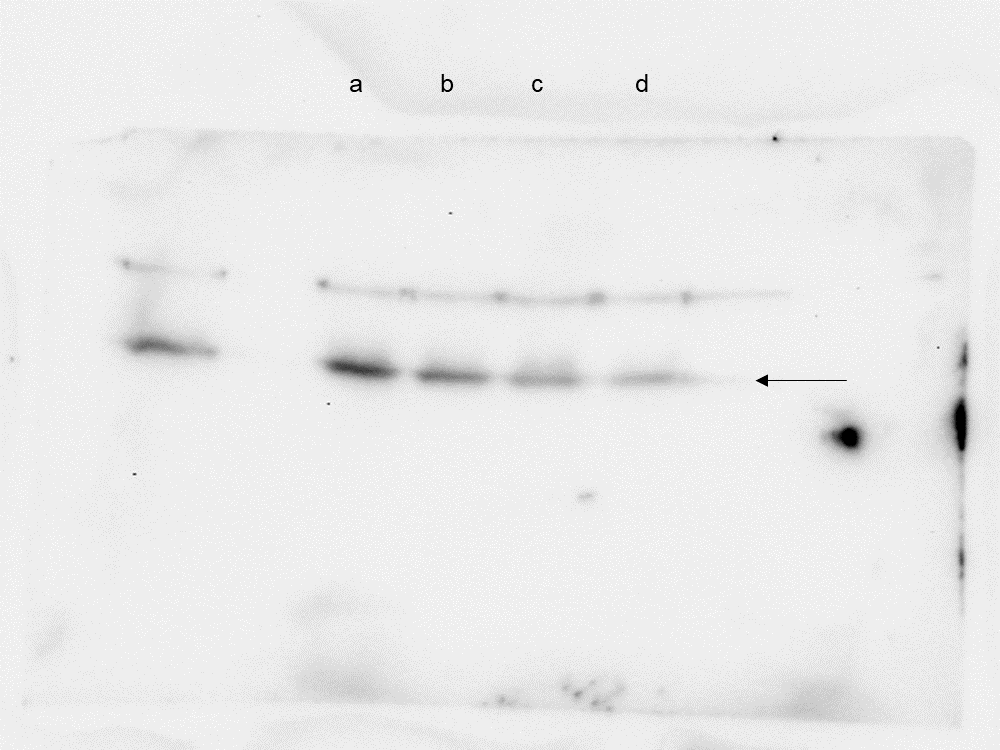


CHOP


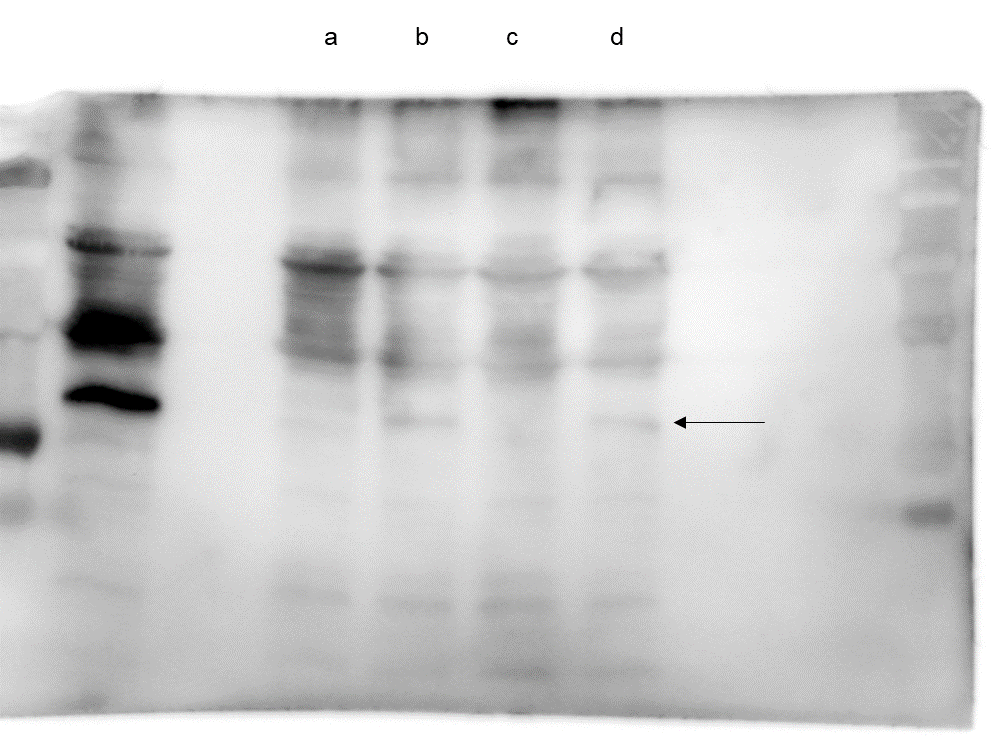


Tubulin


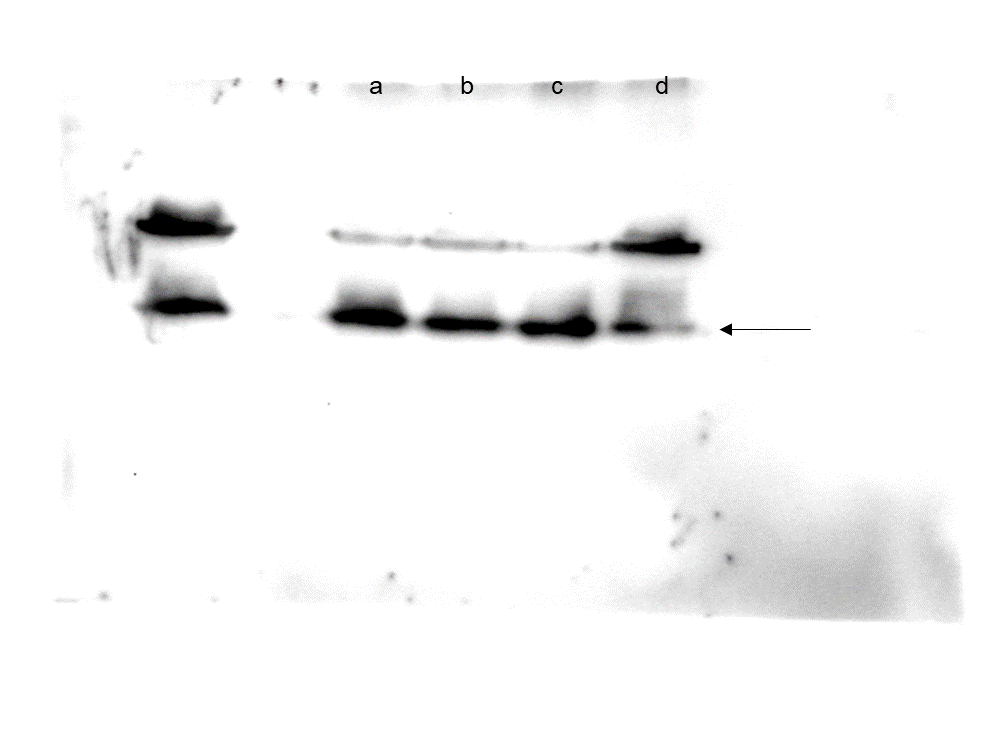


CHOP


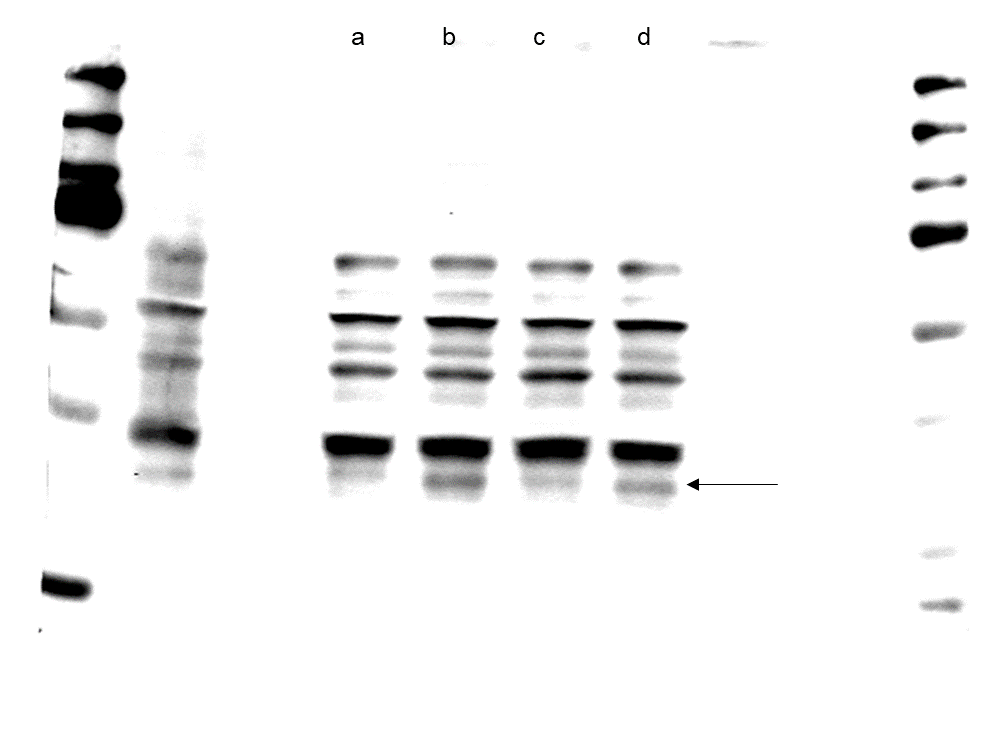


Tubulin


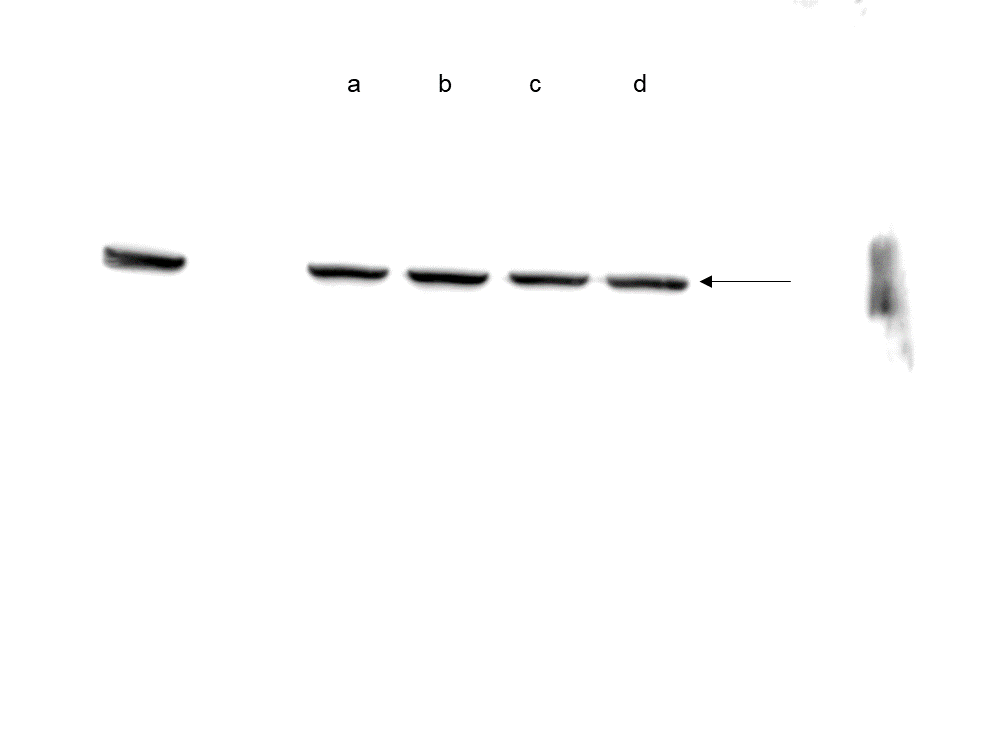


CHOP


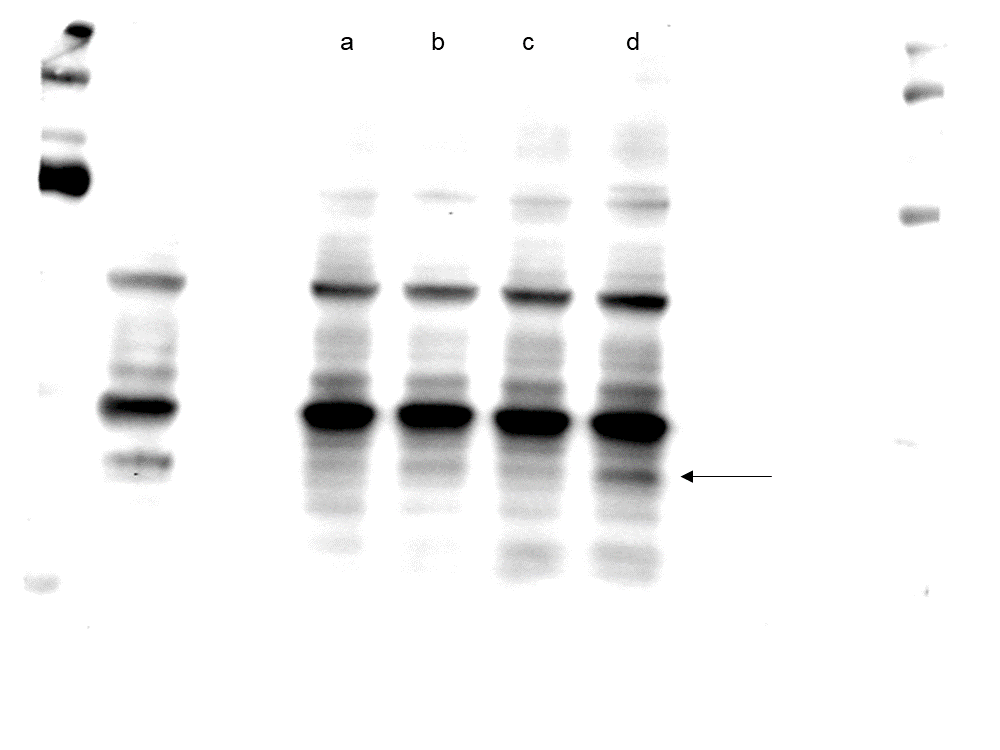


Tubulin


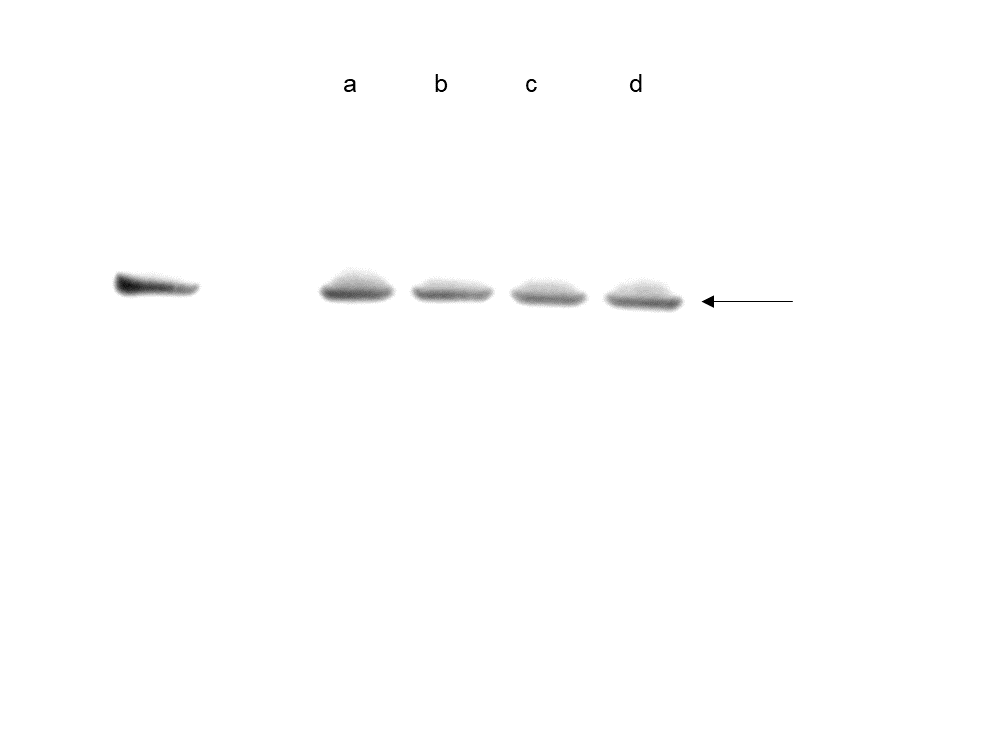


CHOP


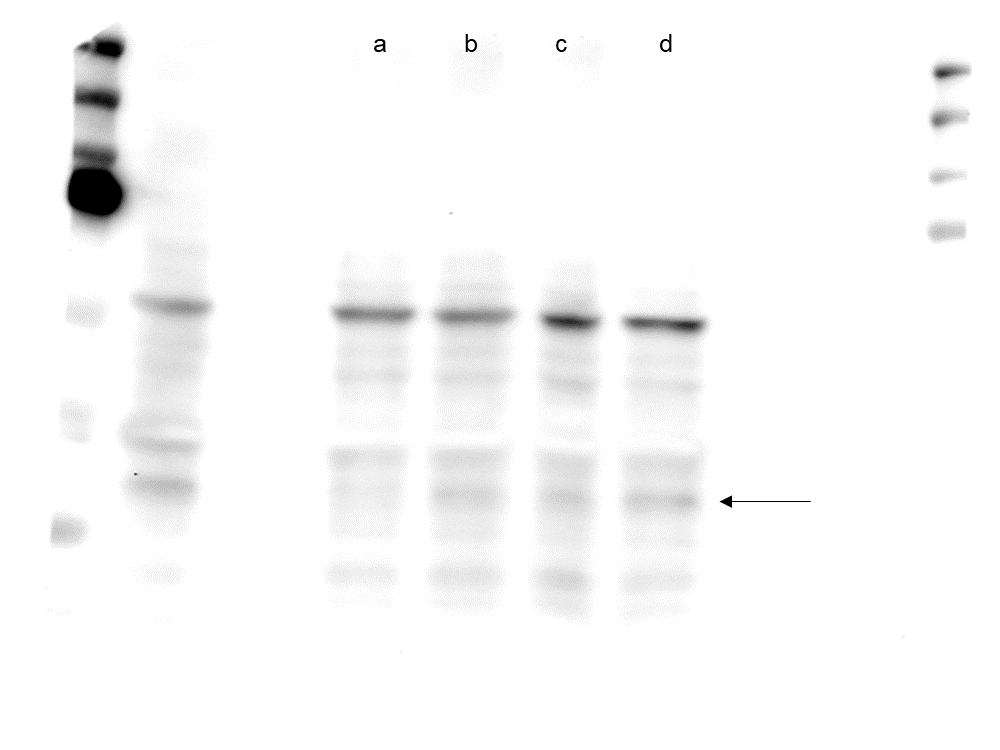


Tubulin


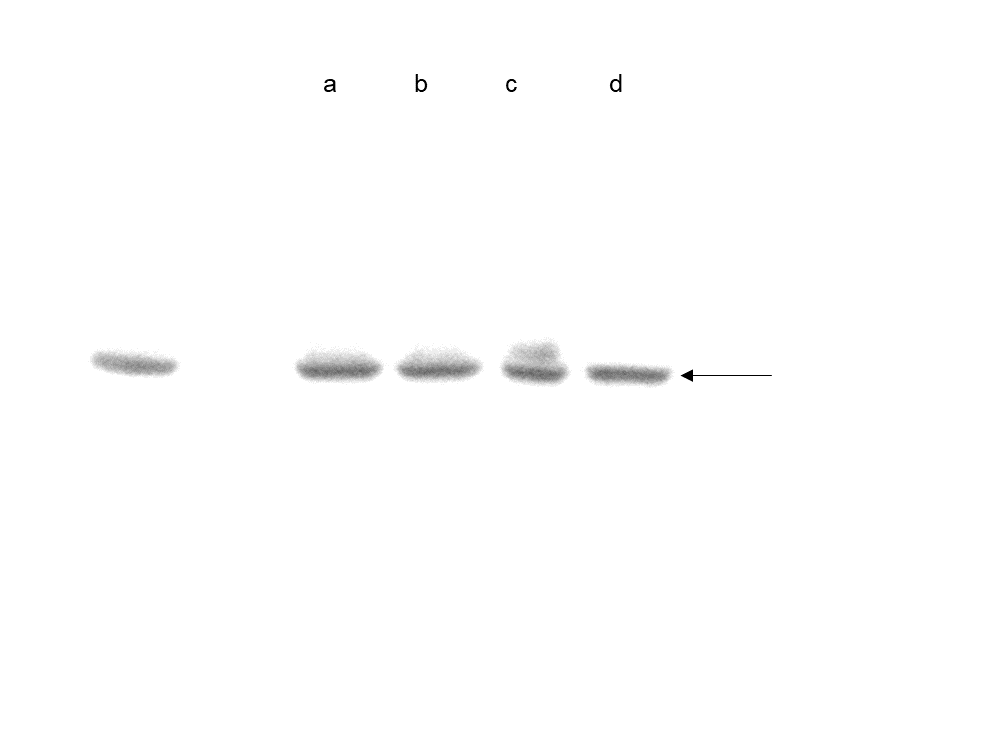


Supplemental figure 5.

All membranes for western blotting for Figure 5.

e; 20% CSE, f; 20% CSE + homocysteine 5 mM, g; 20% CSE + homocysteine 5 mM + vitamin B12 and folate 5 μM, h; 20% CSE + homocysteine 5 mM + vitamin B12 and folate 25 μM, i; 20% CSE + homocysteine 5 mM + vitamin B12 and folate 50 μM

Homocysteine


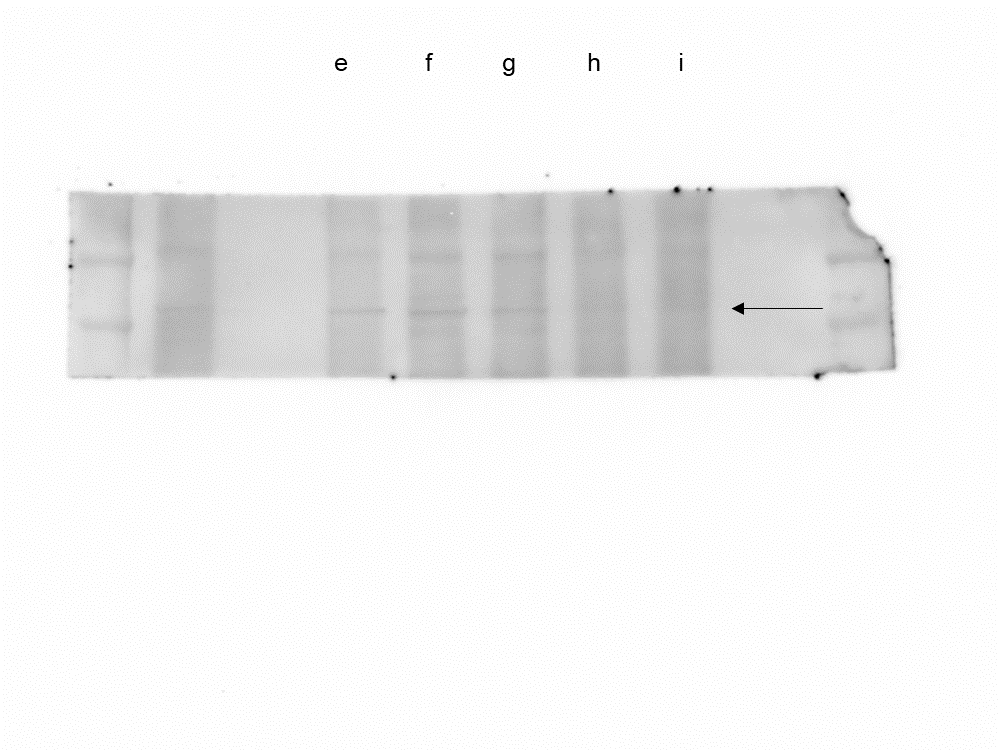


Tubulin


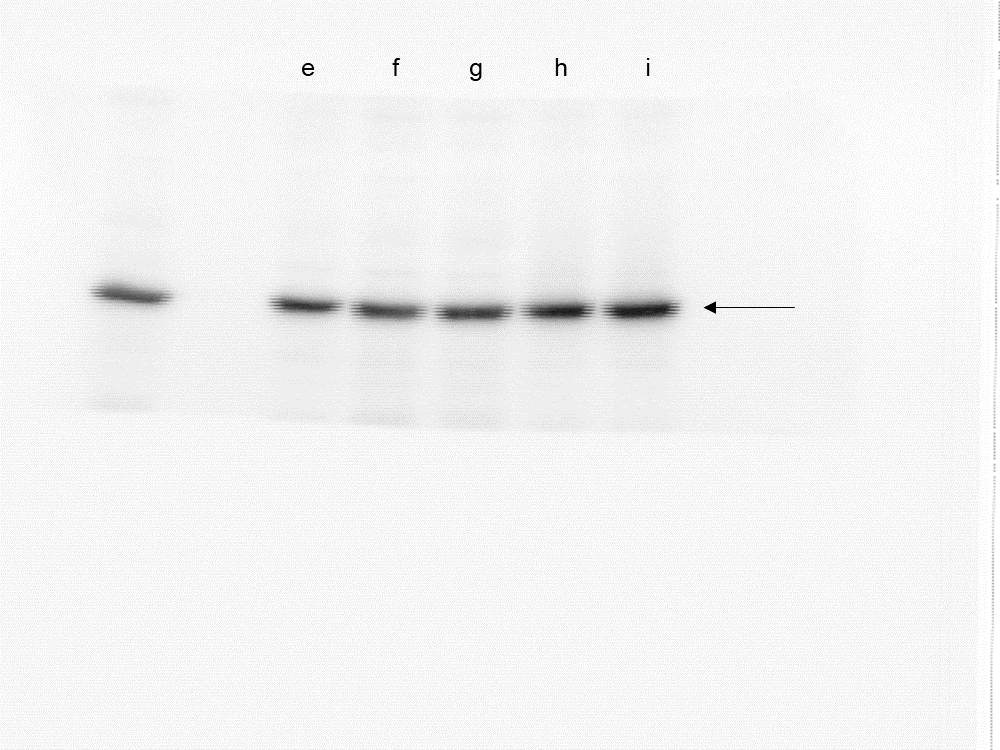


Homocysteine


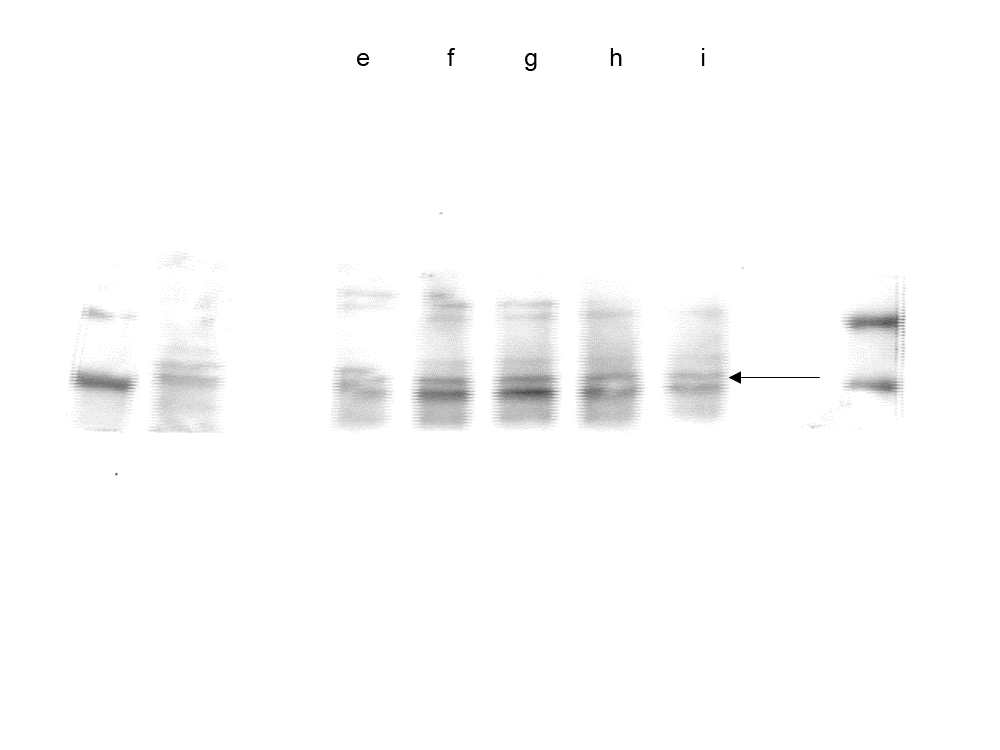


Tubulin


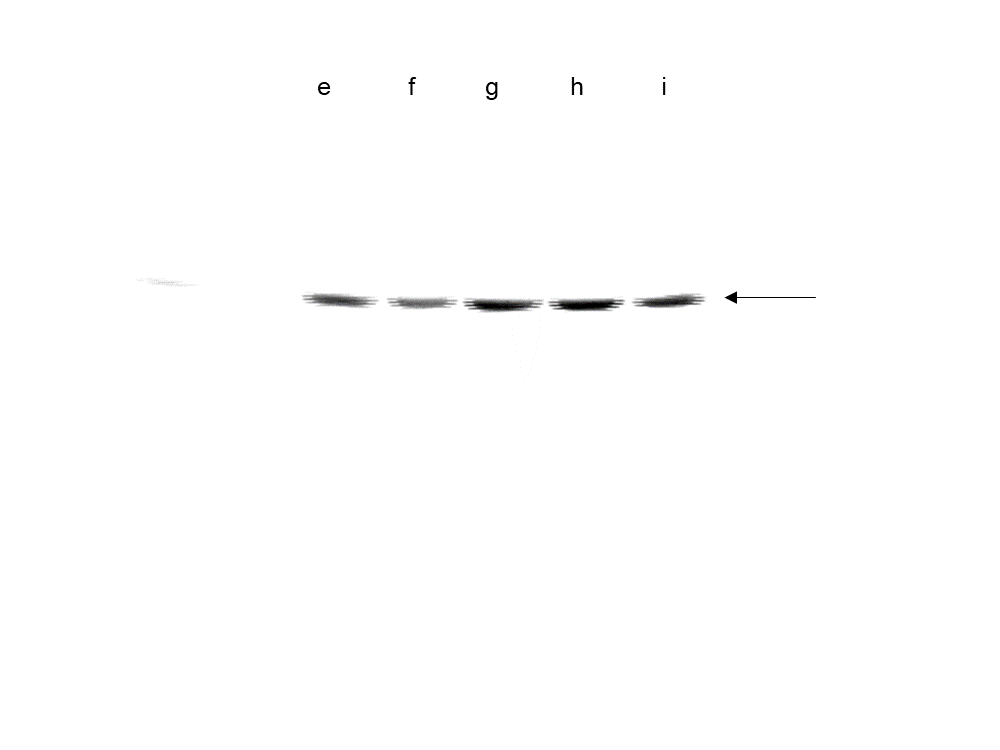


Homocysteine


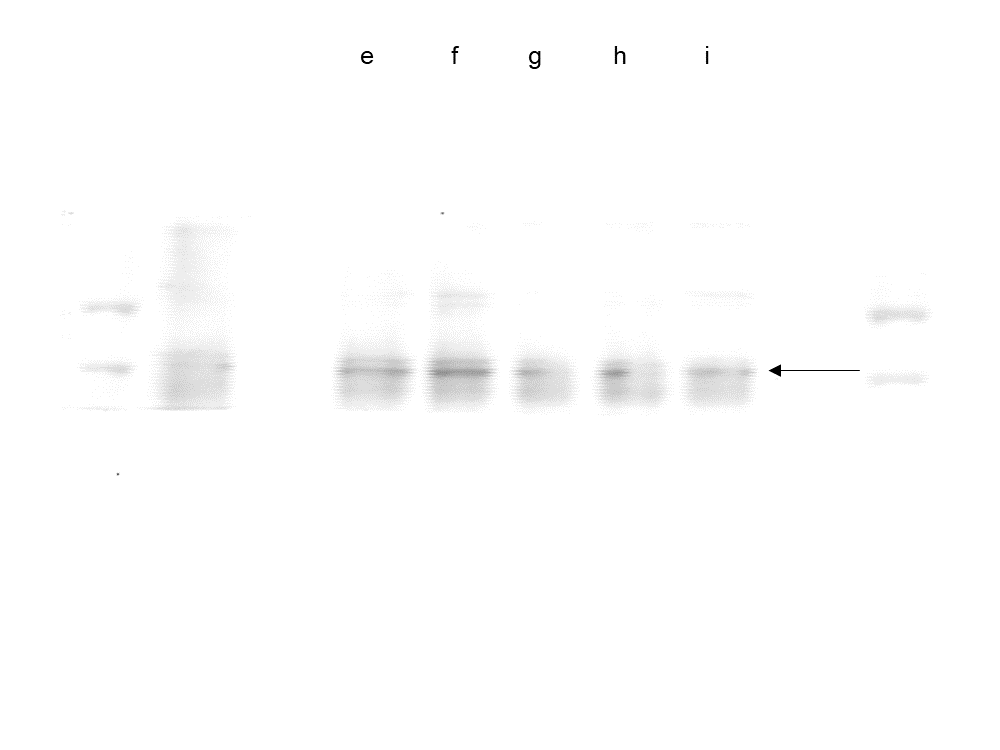


Tubulin


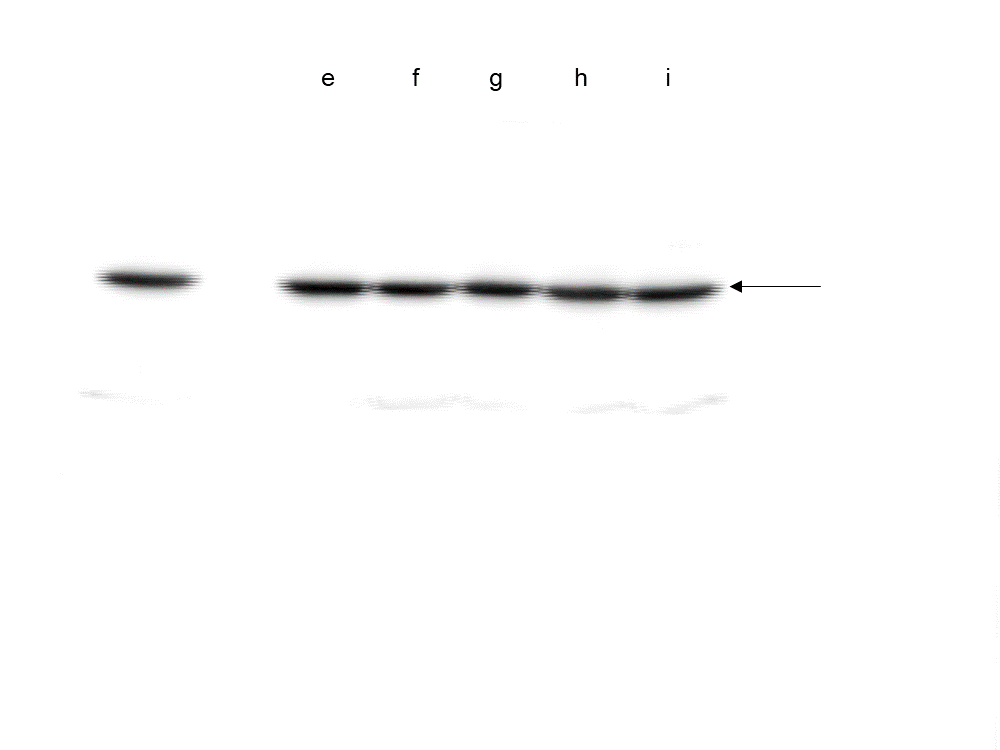


Homocysteine


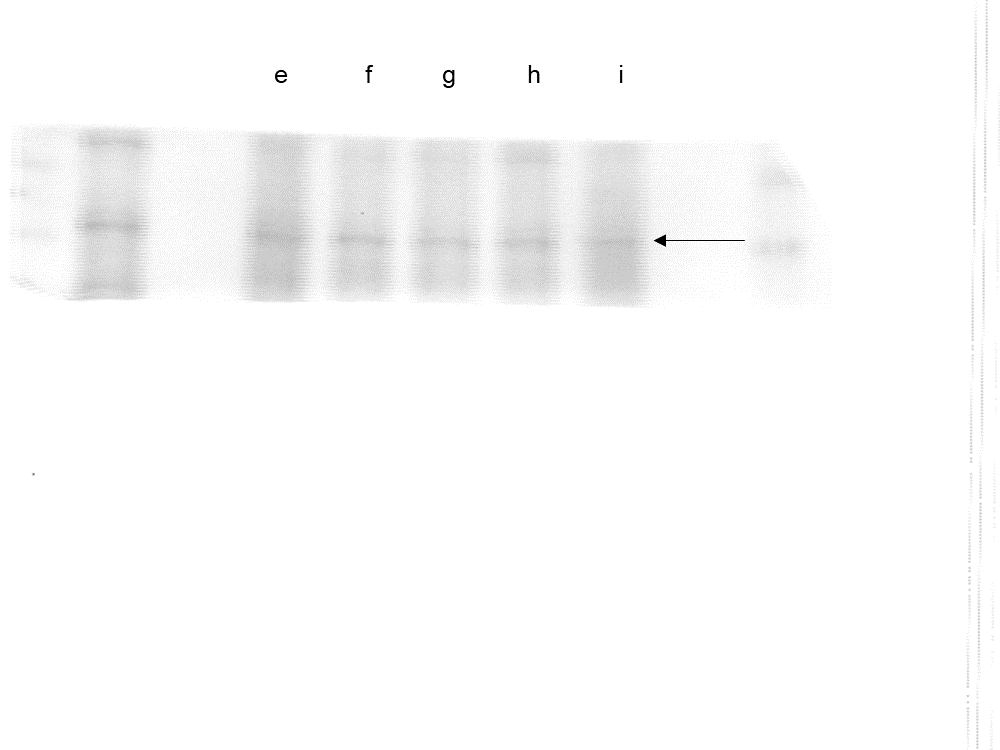


Tubulin


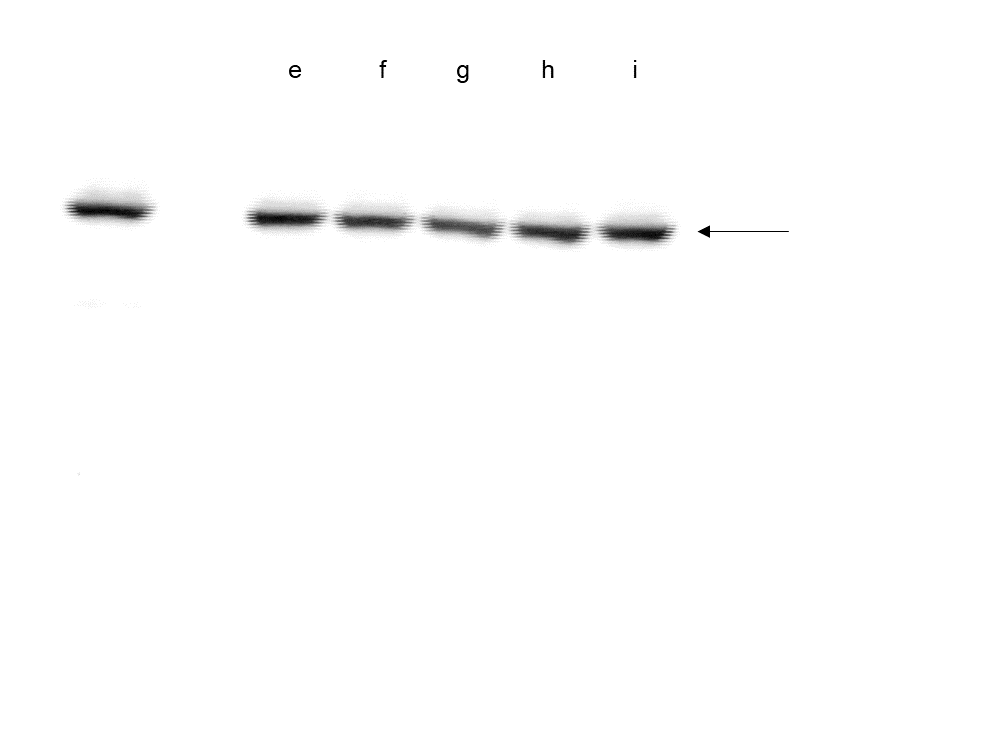


Homocysteine


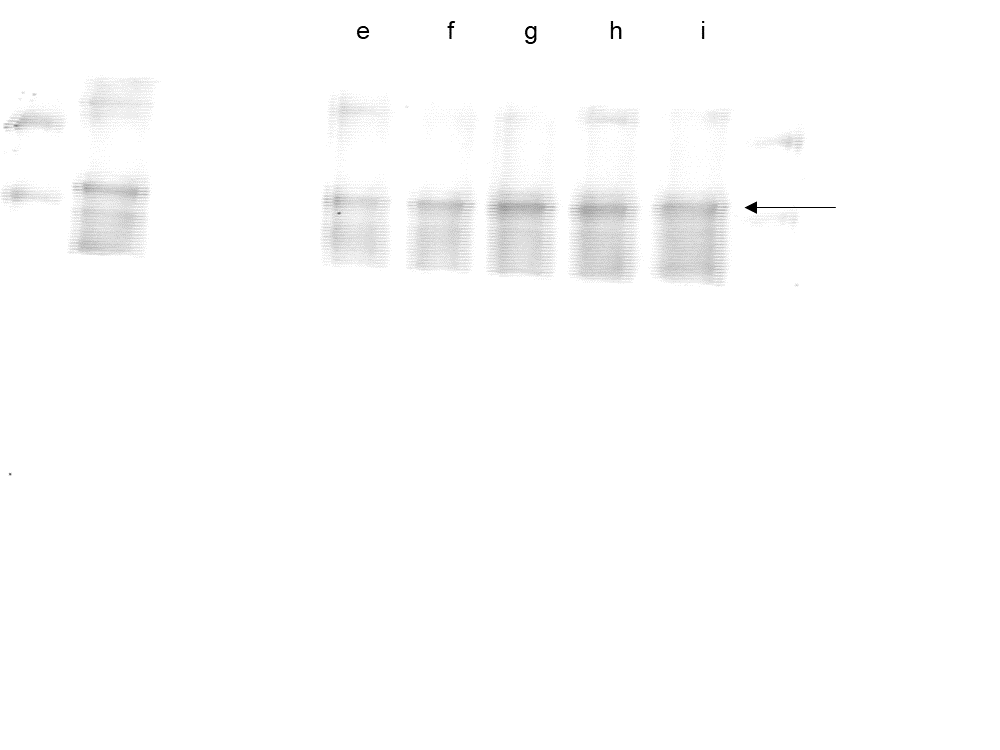


Tubulin


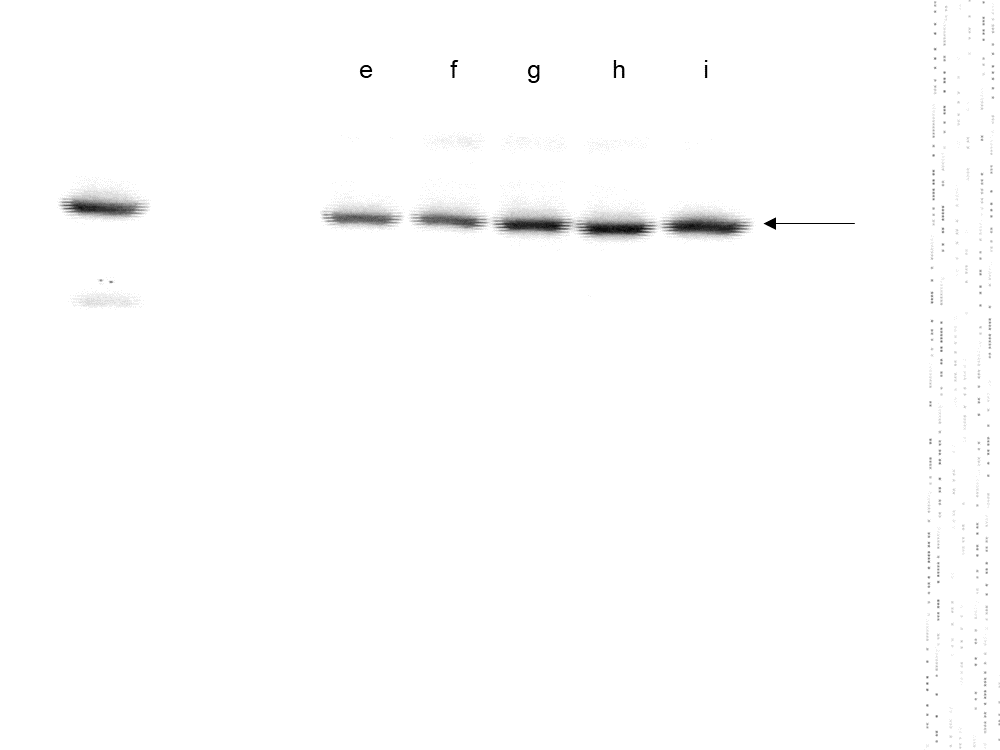


GRP78


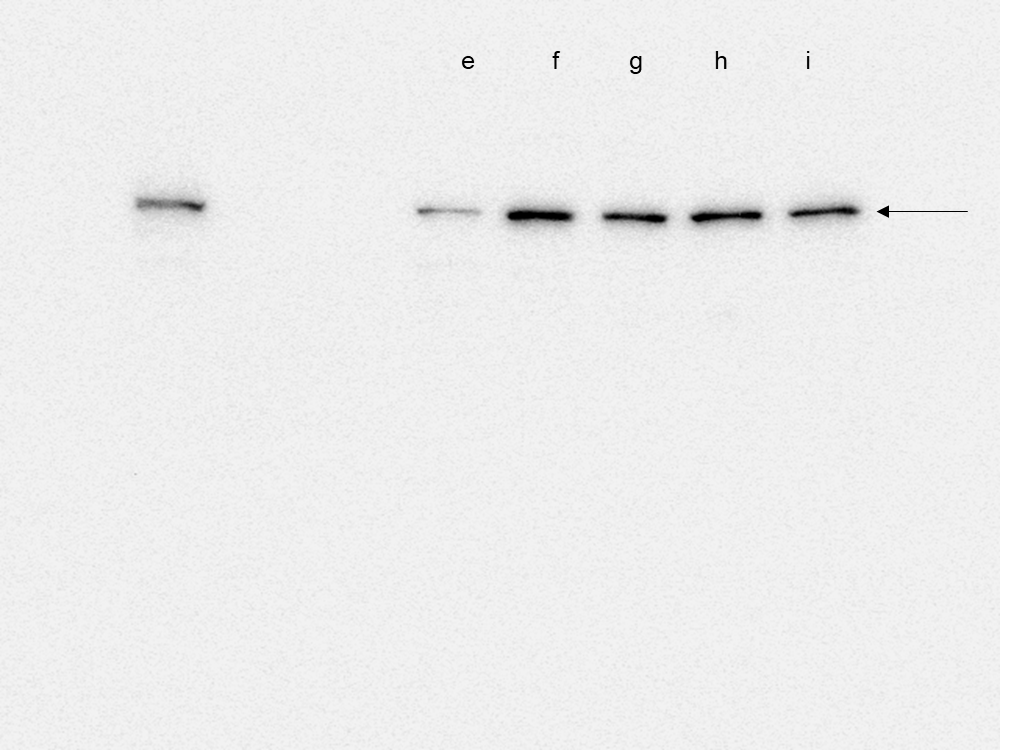


Tubulin


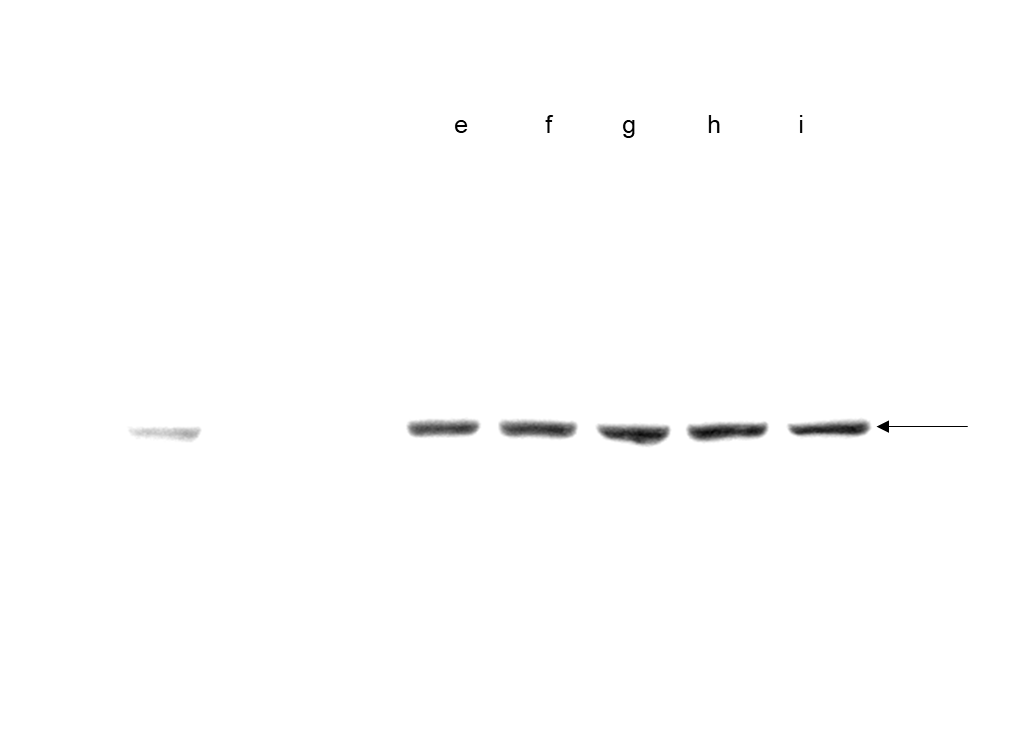


GRP78


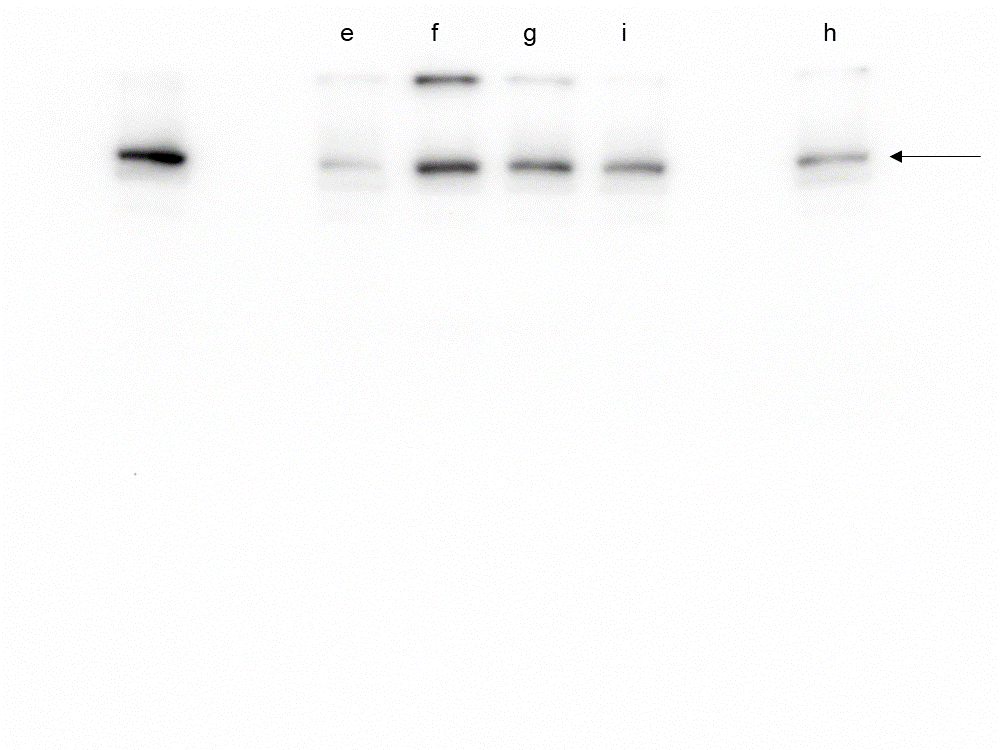


Tubulin


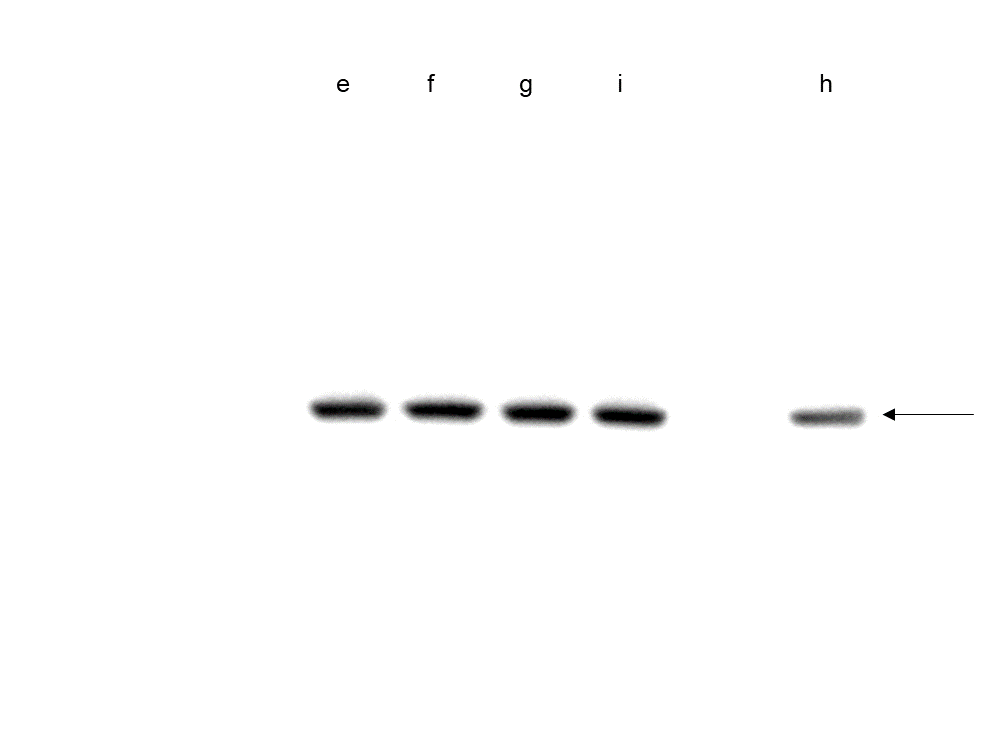


GRP78


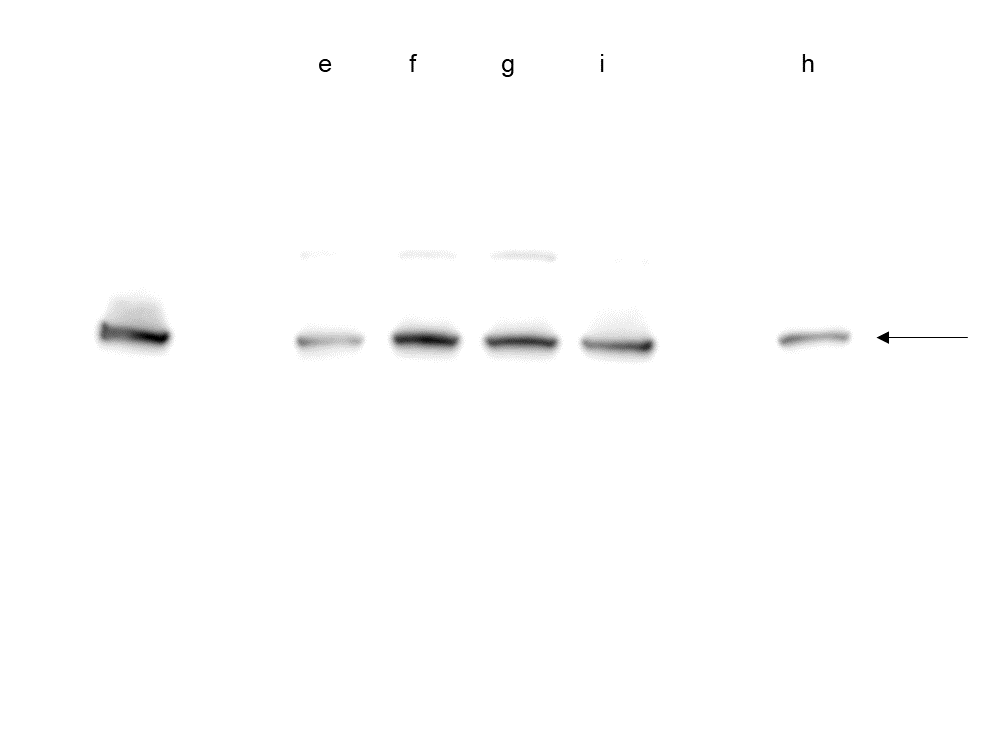


Tubulin


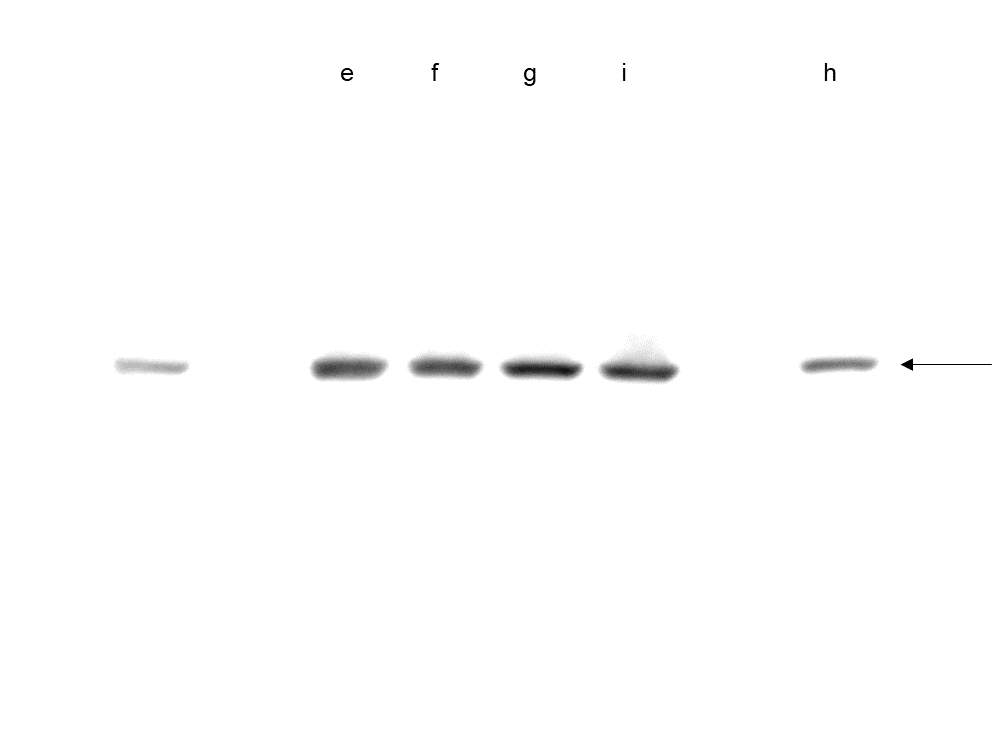


GRP78


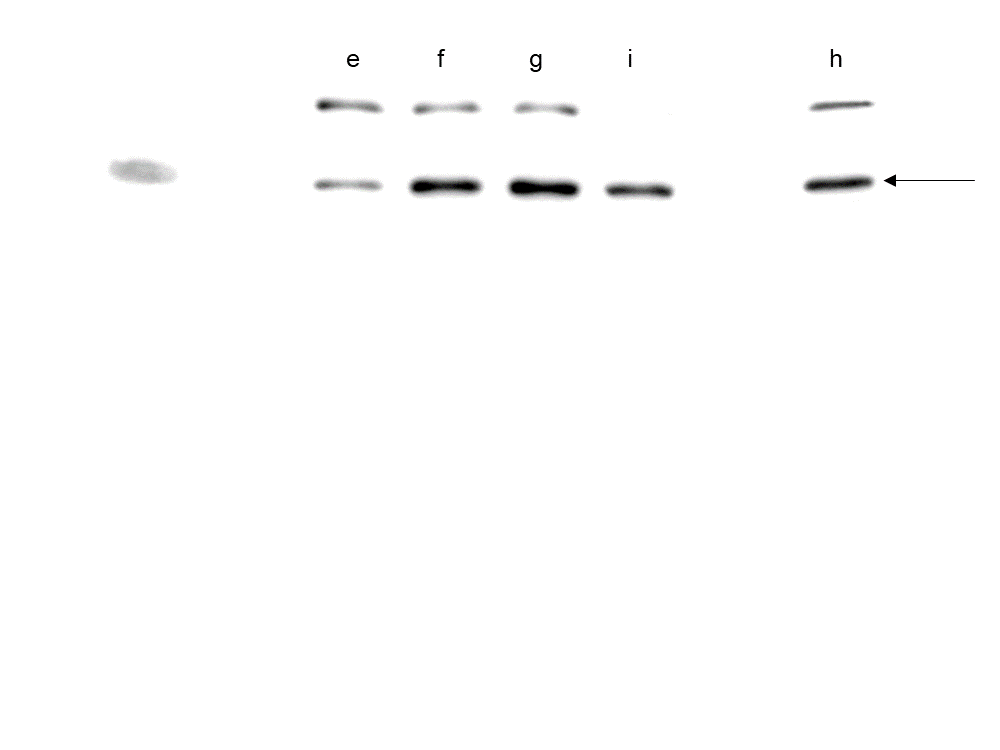


Tubulin


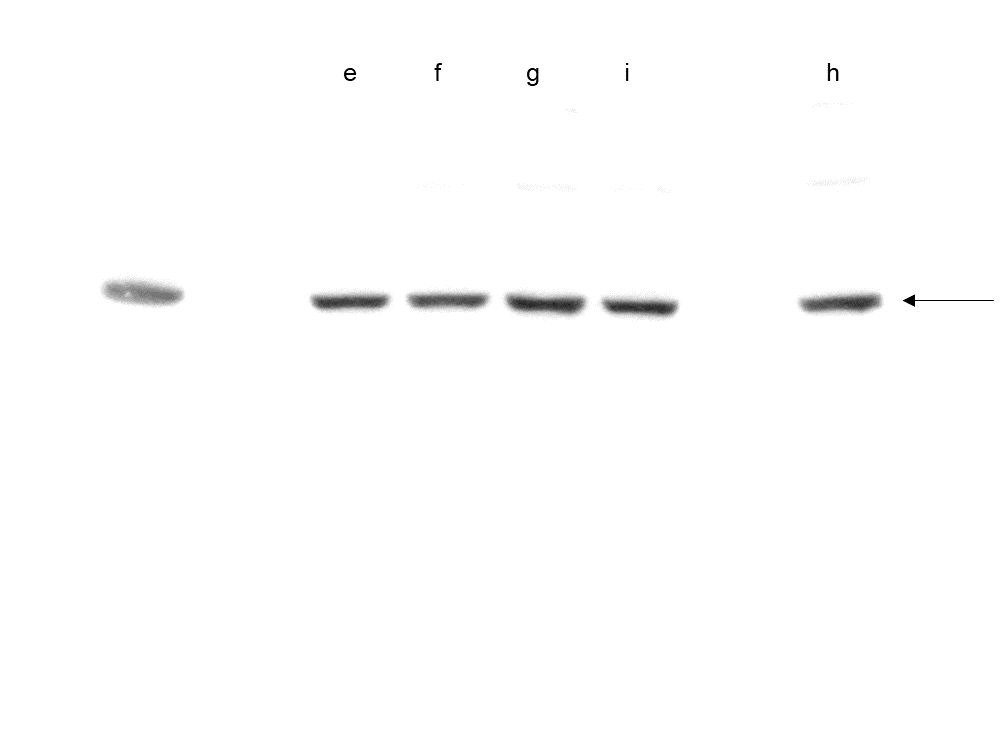


GRP78


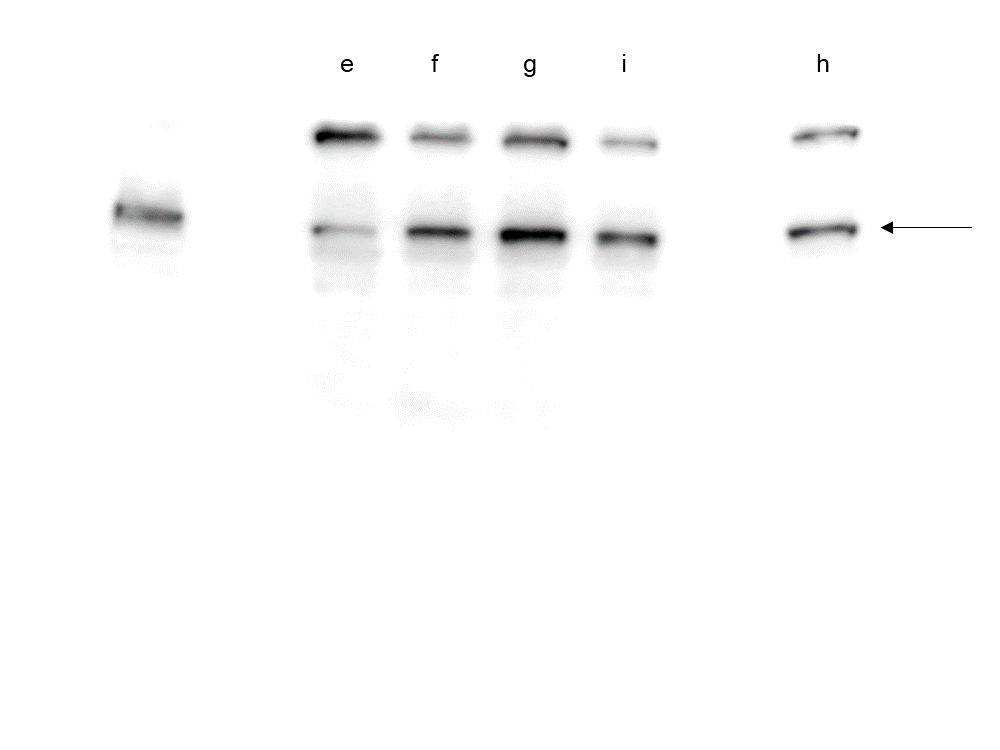


Tubulin


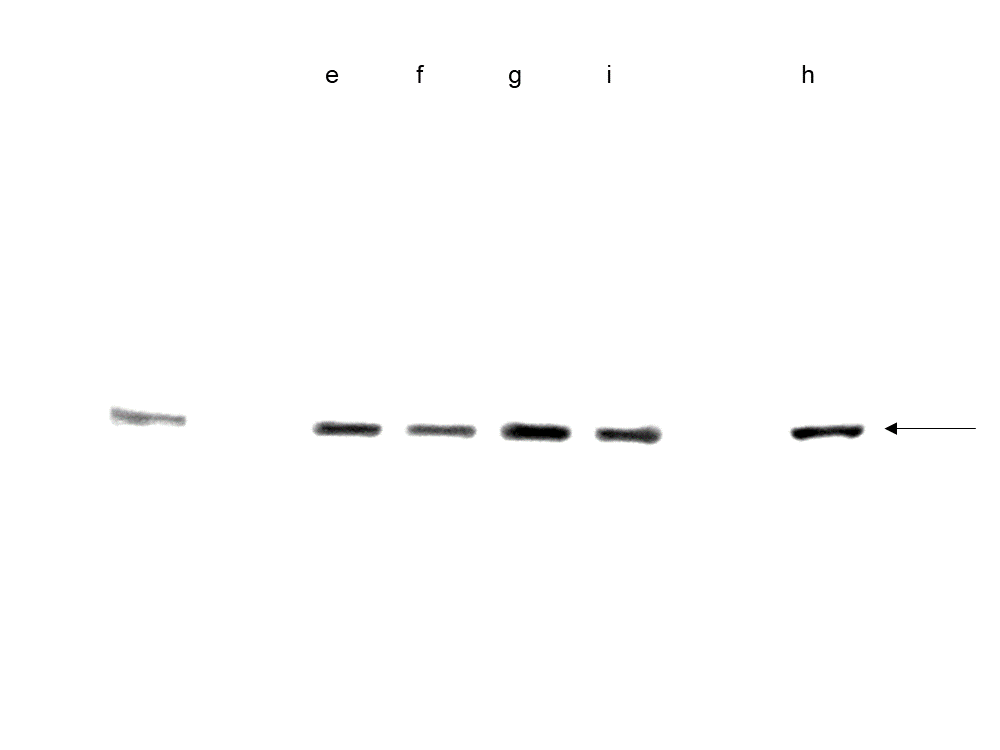


p-IRE1


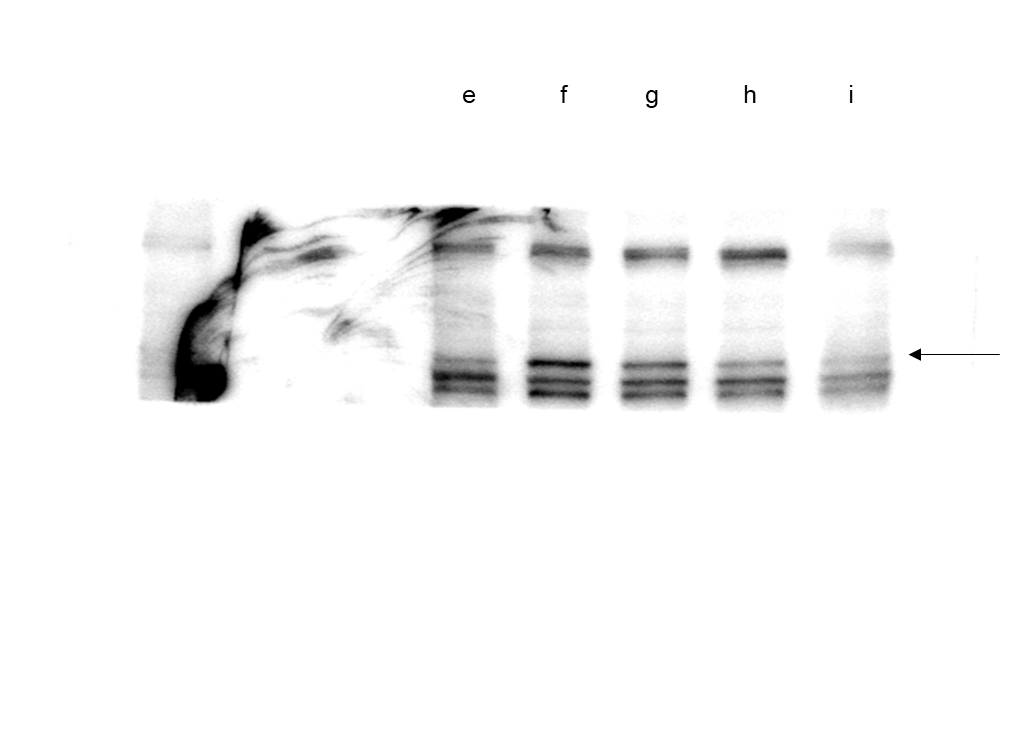


t-IRE1


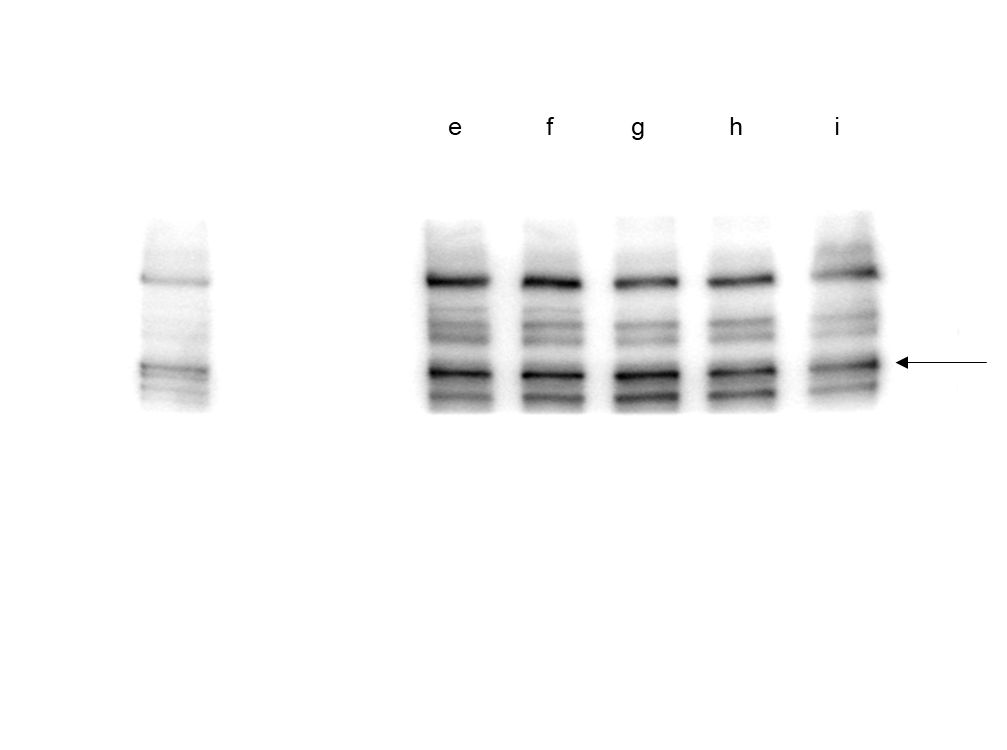


p-IRE1


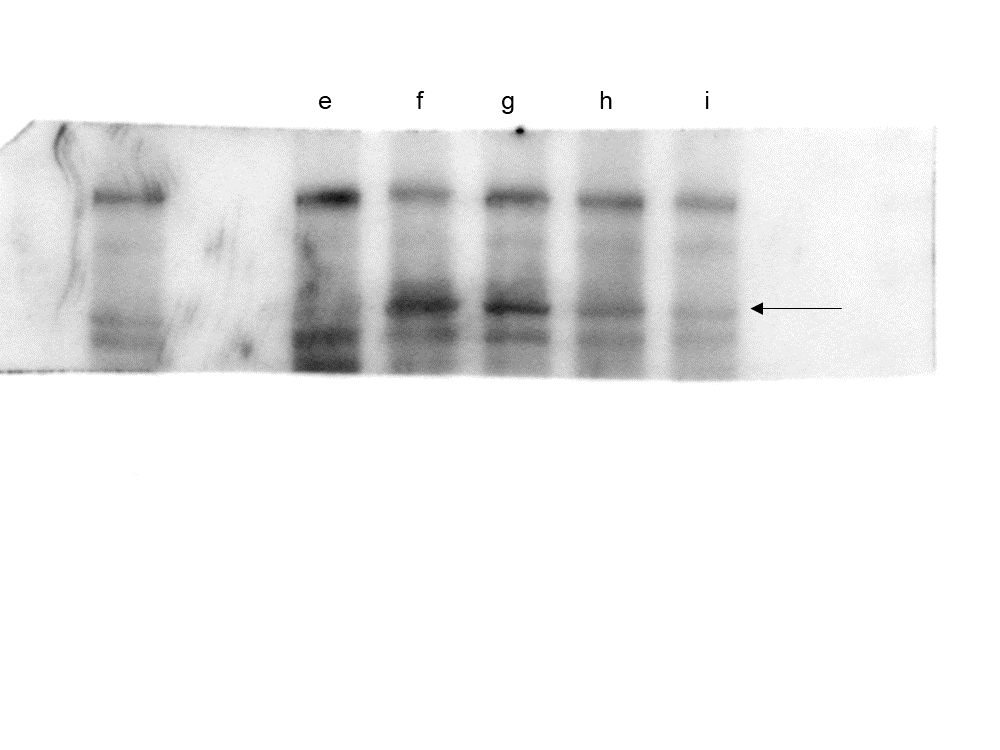


t-IRE1


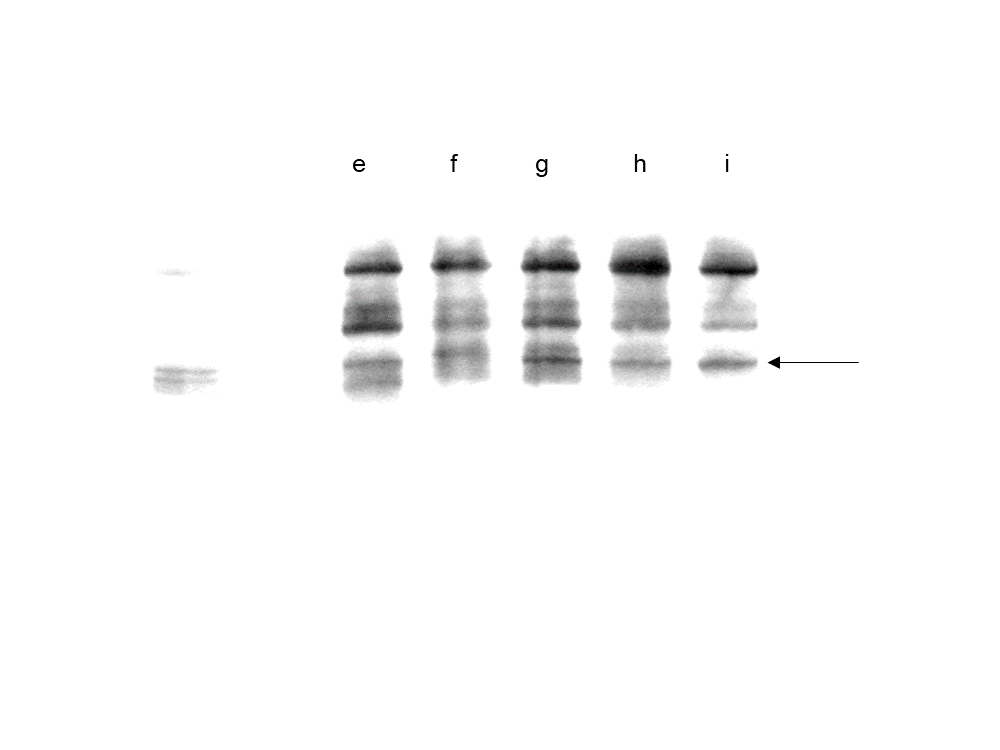


p-IRE1


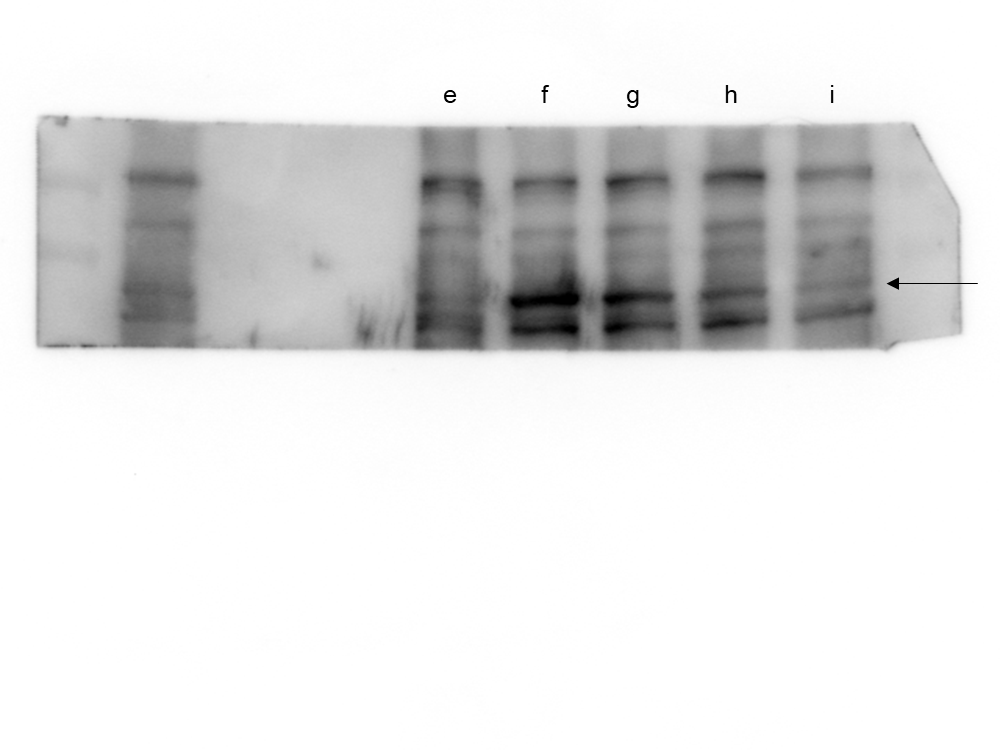


t-IRE1


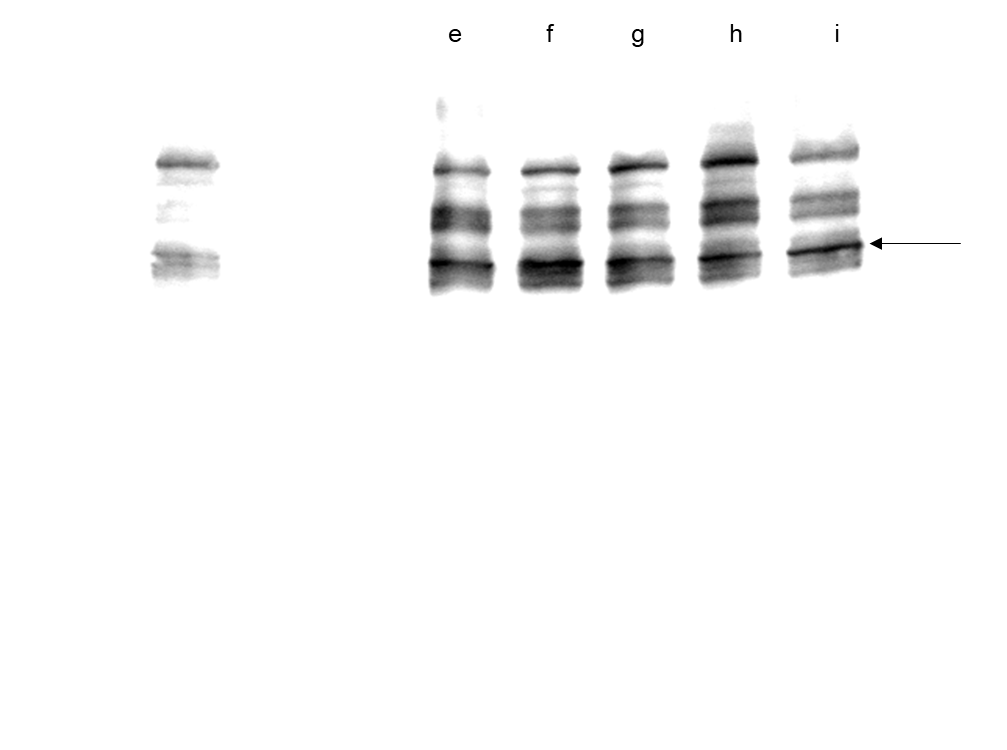


p-IRE1


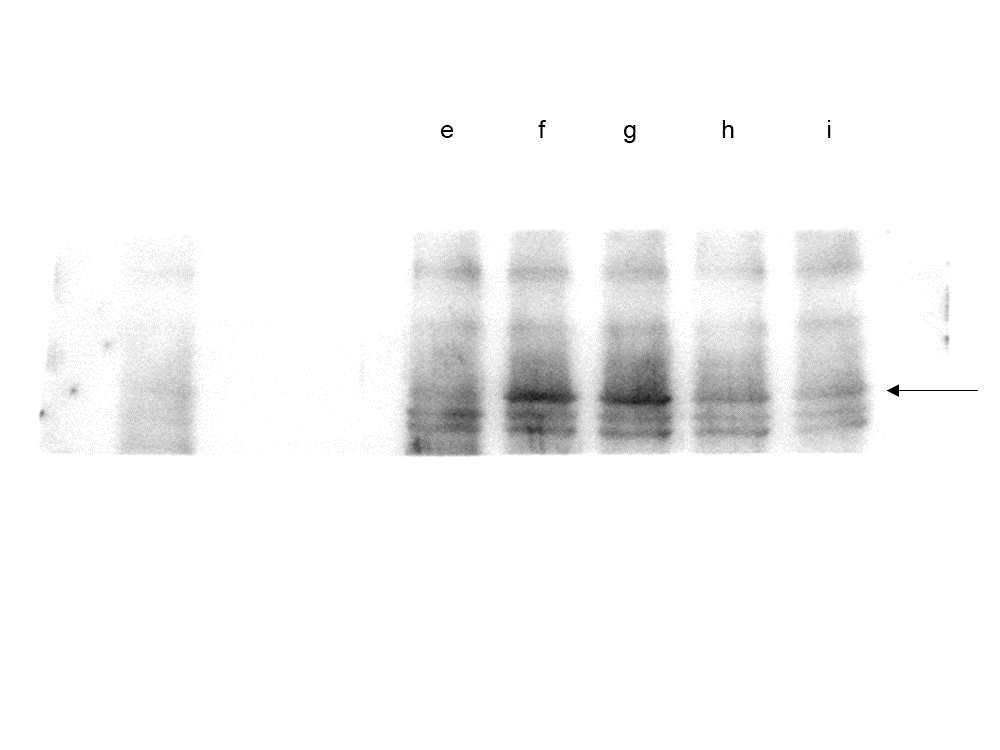


t-IRE1


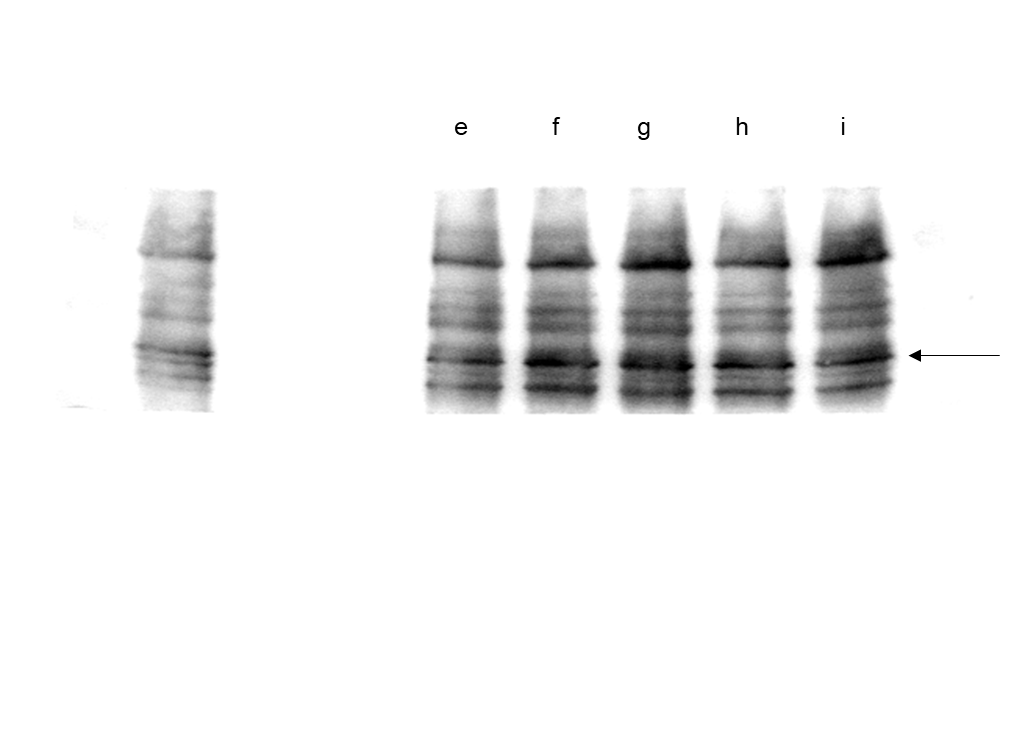


p-IRE1


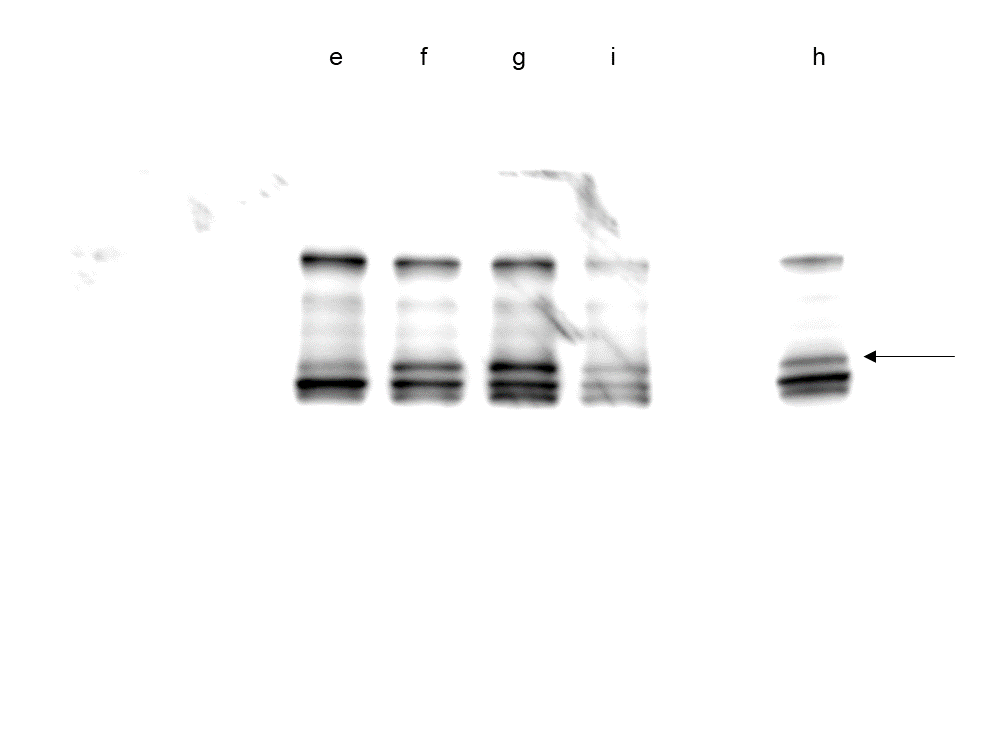


t-IRE1


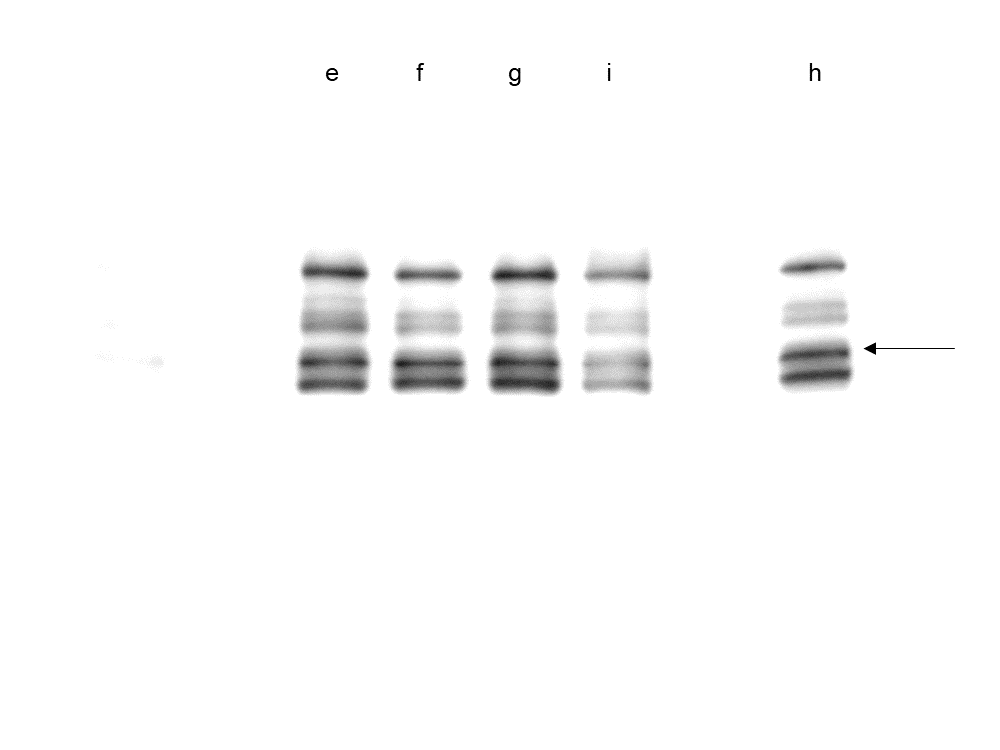


p-eIF2


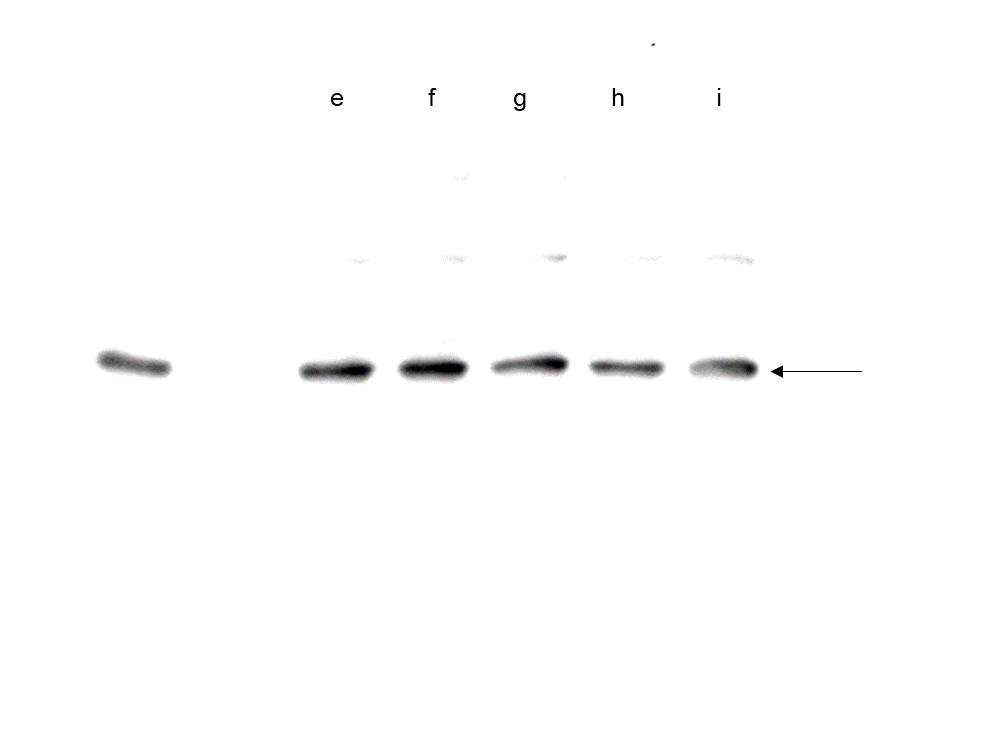


Tubulin


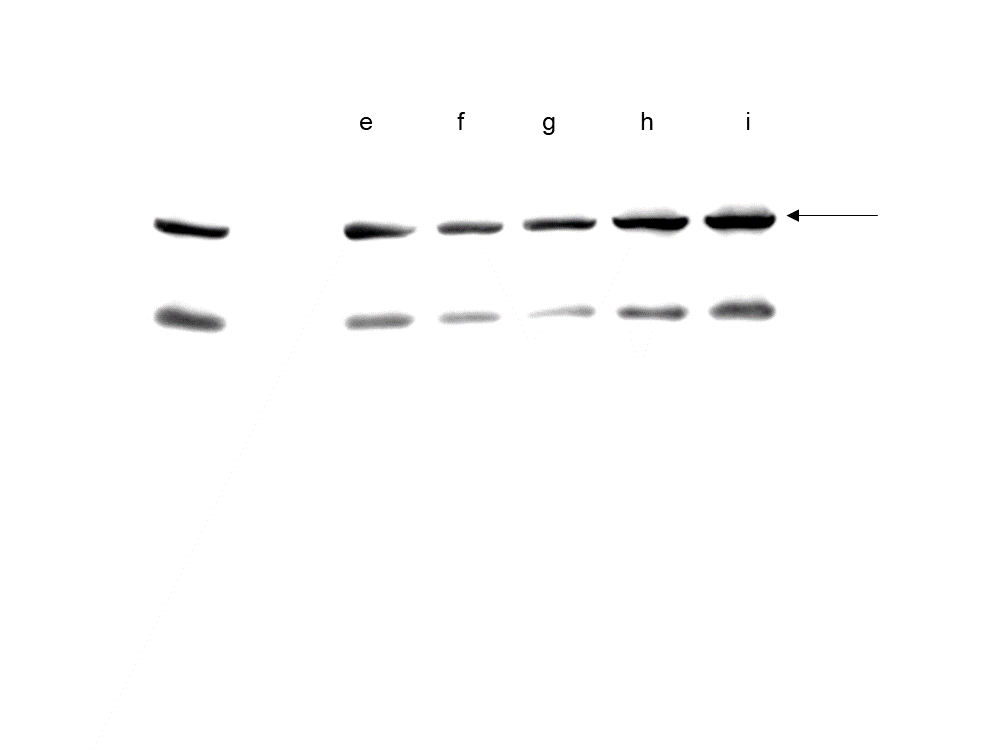


p-eIF2


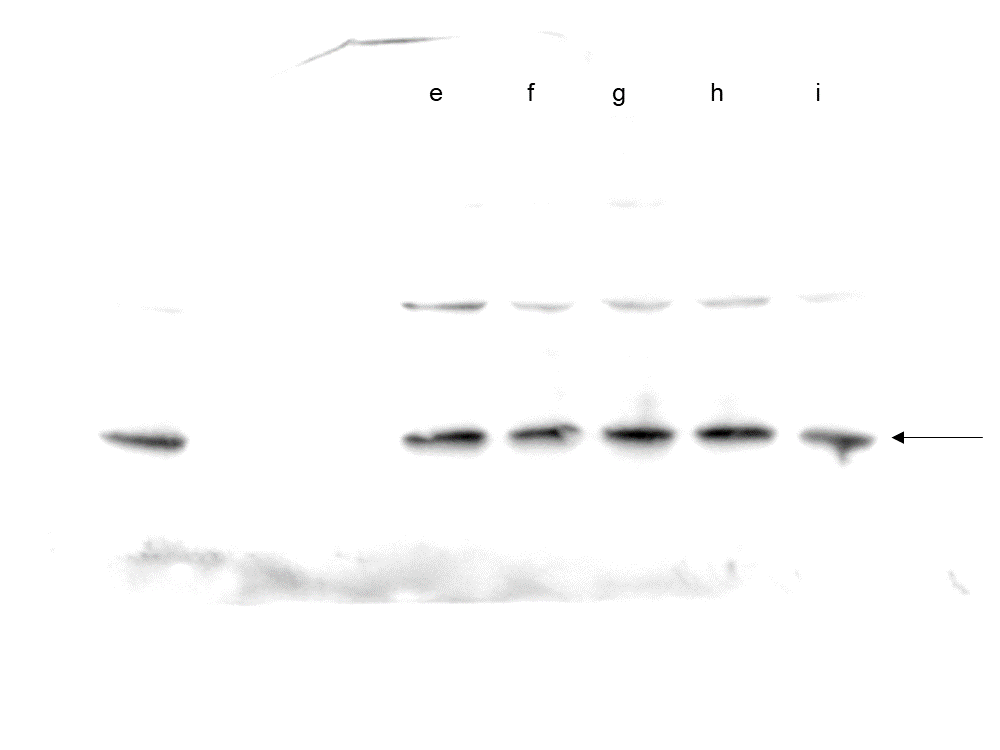


Tubulin


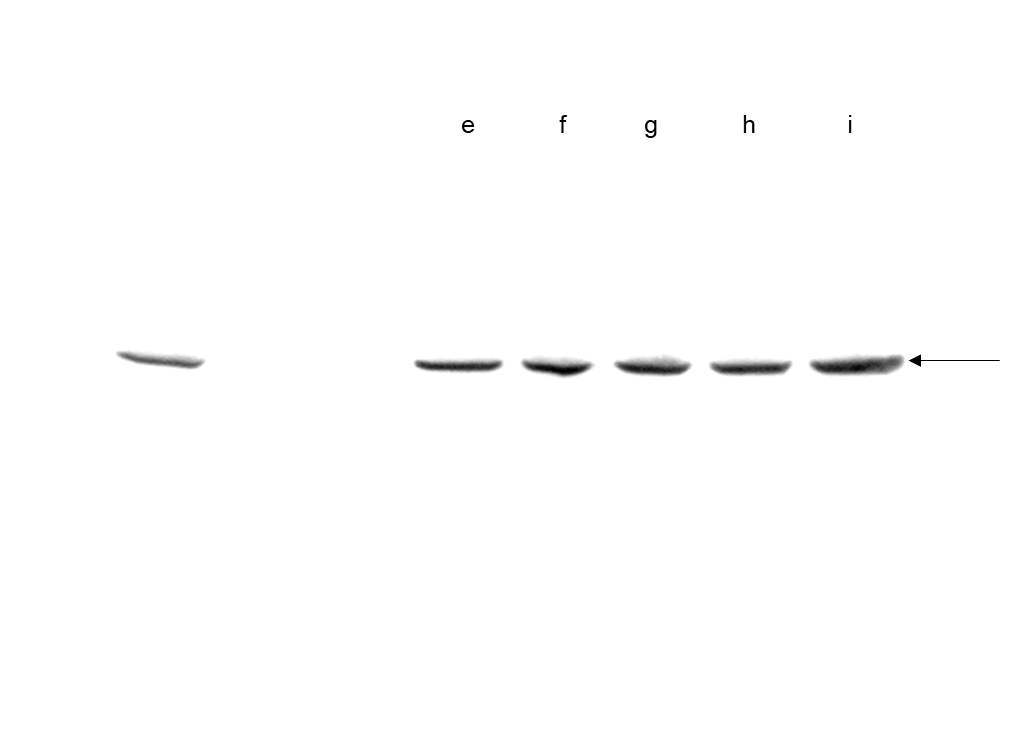


p-eIF2


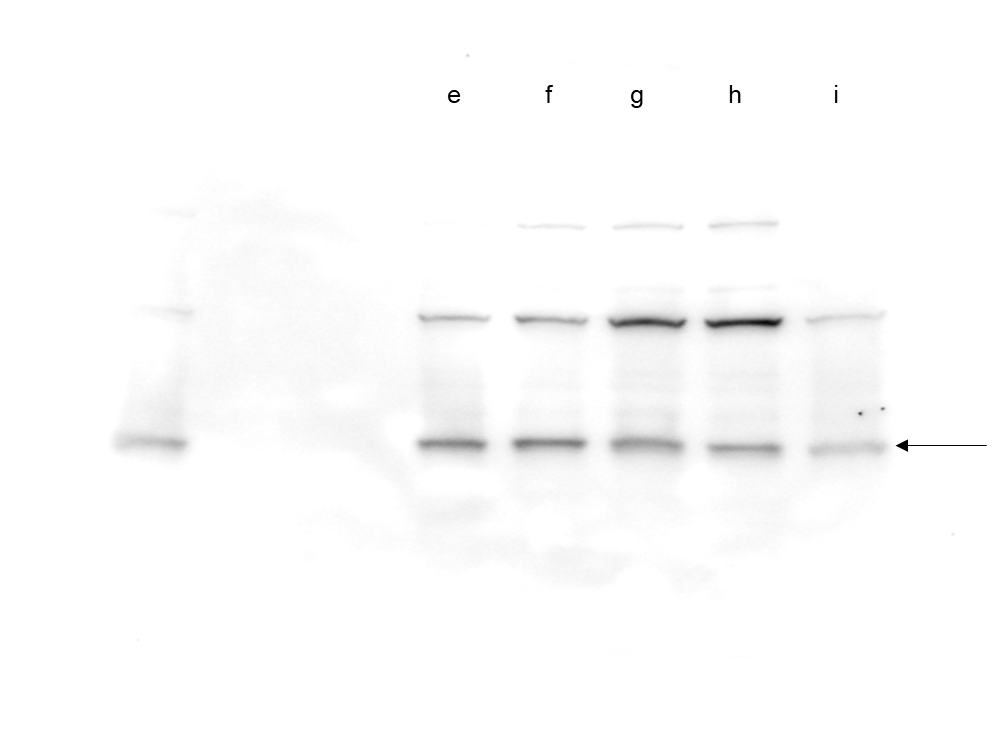


Tubulin


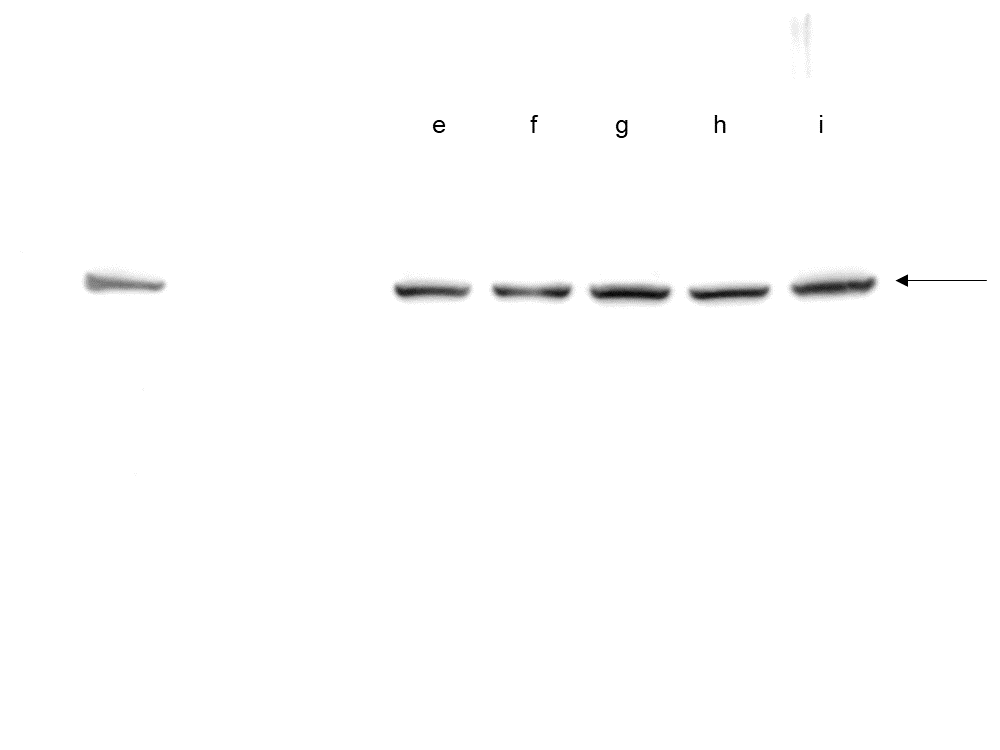


p-eIF2


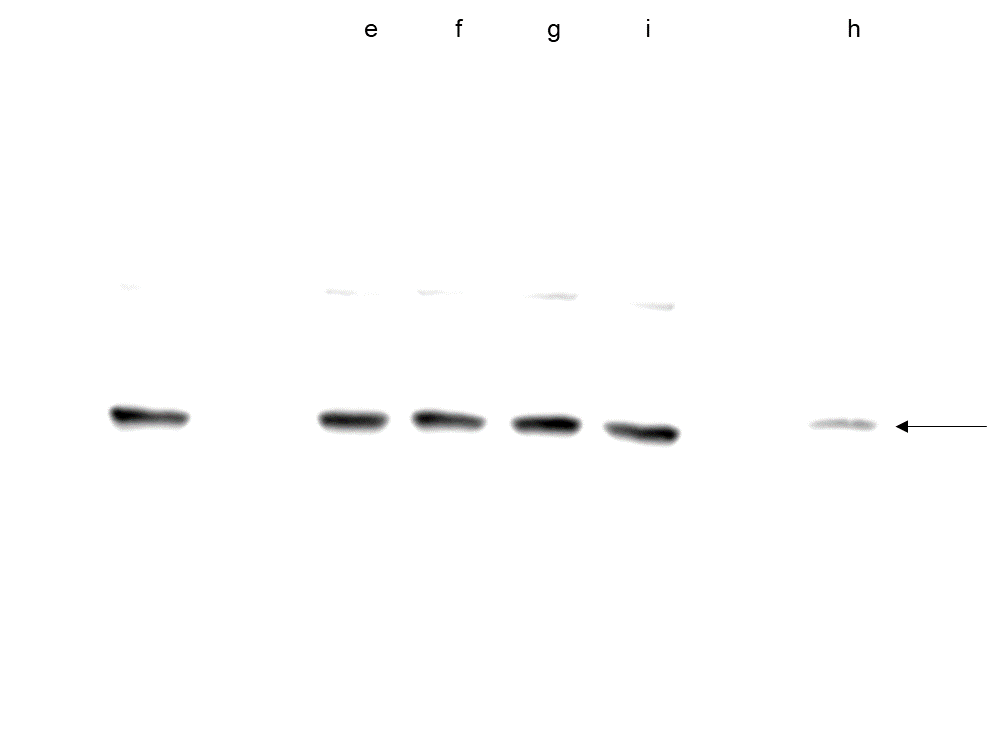


Tubulin


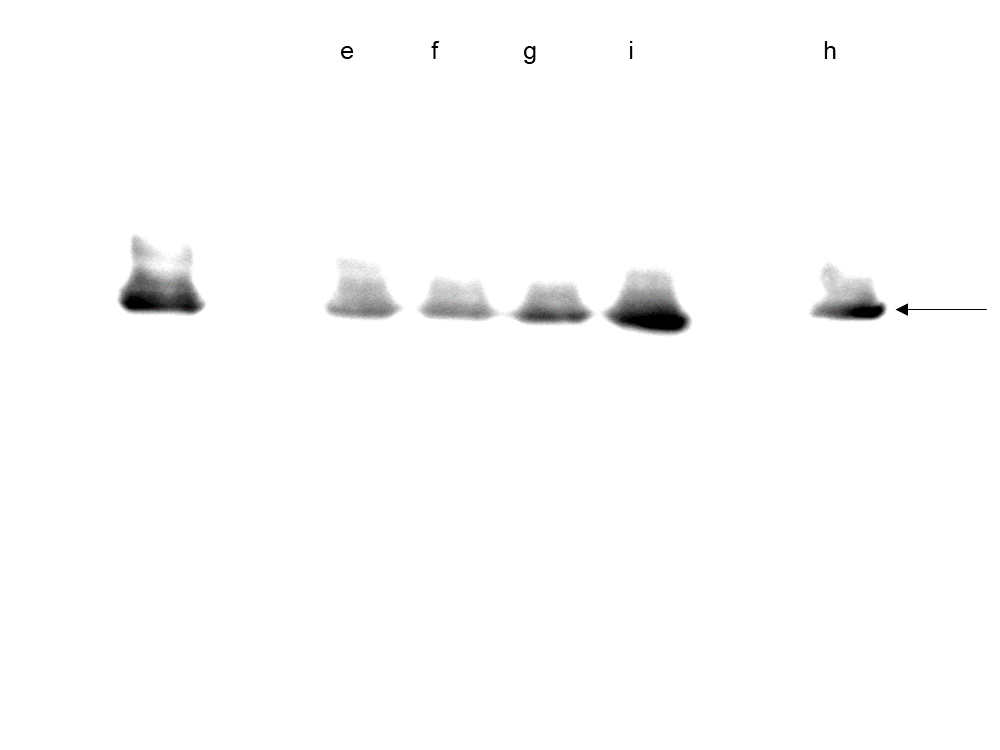


p-eIF2


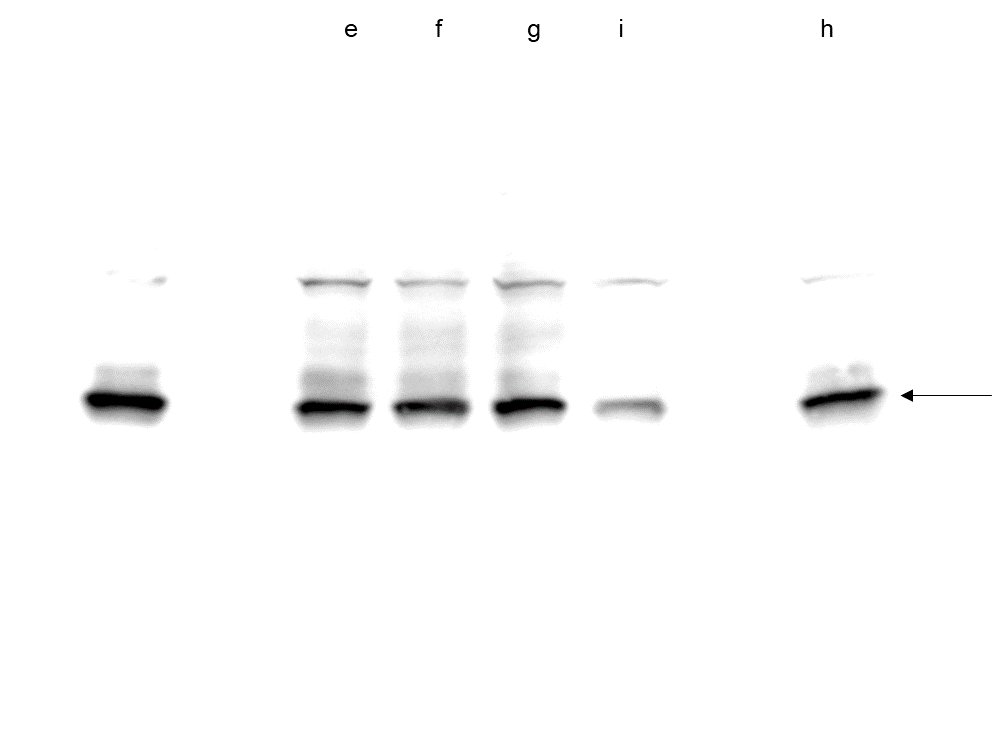


Tubulin


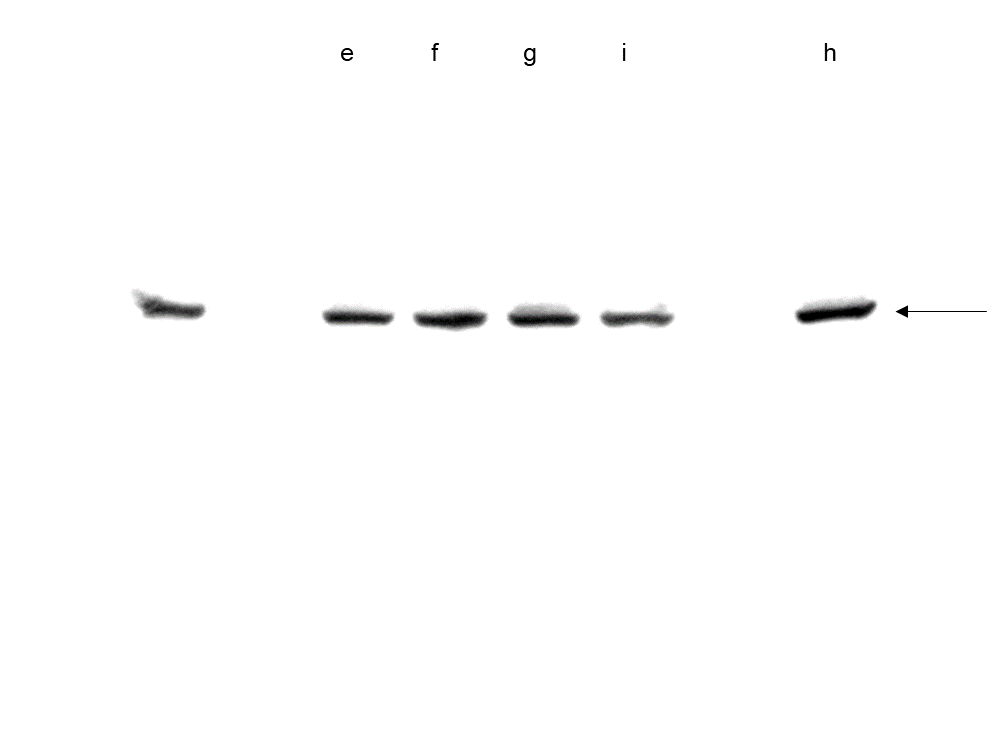


CHOP


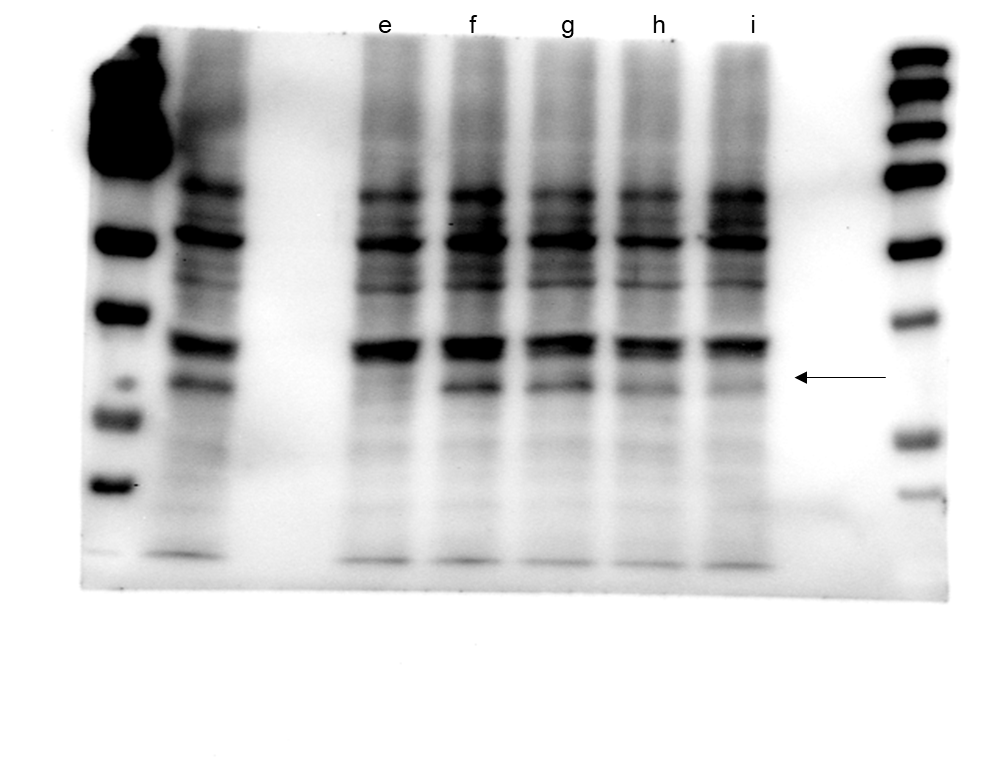


Tubulin


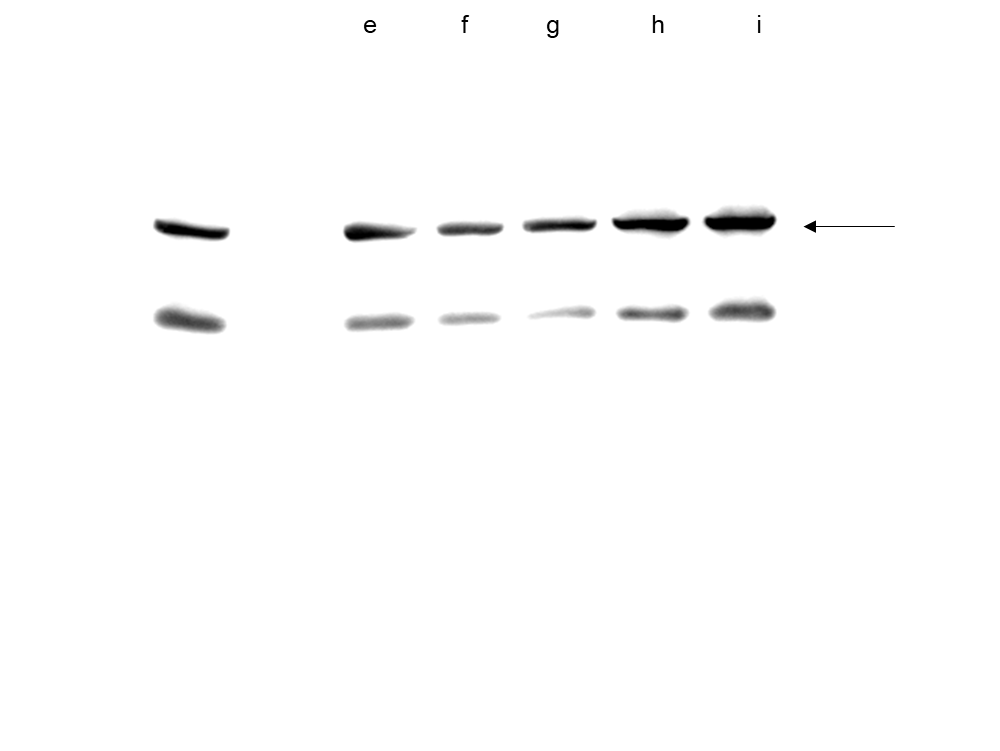


CHOP


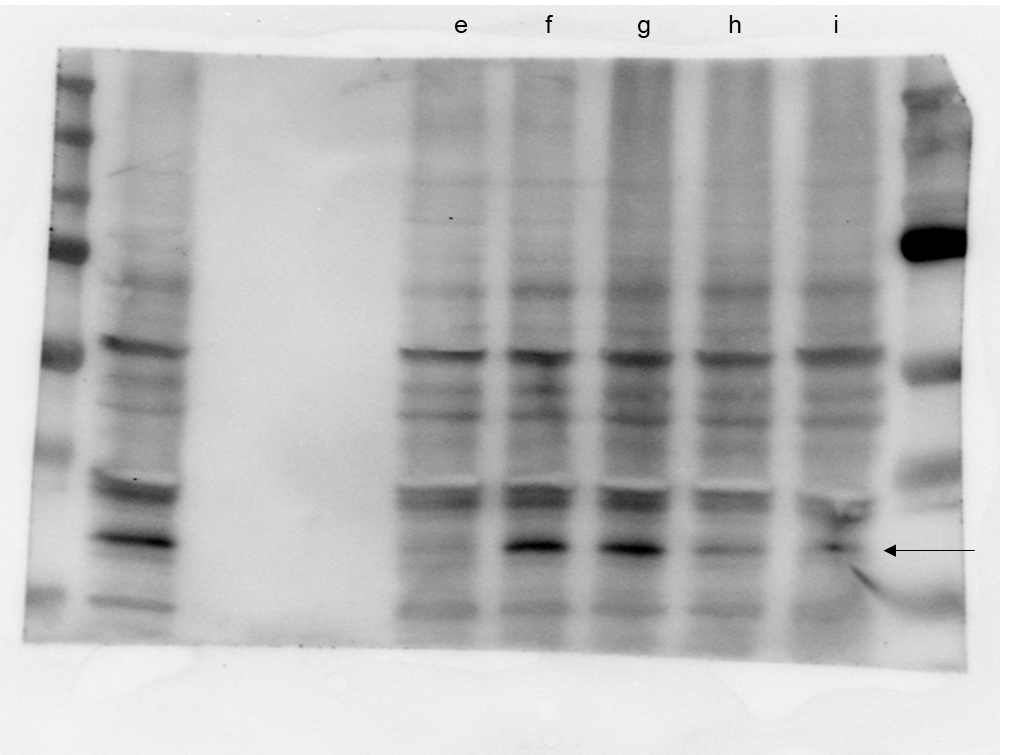


Tubulin


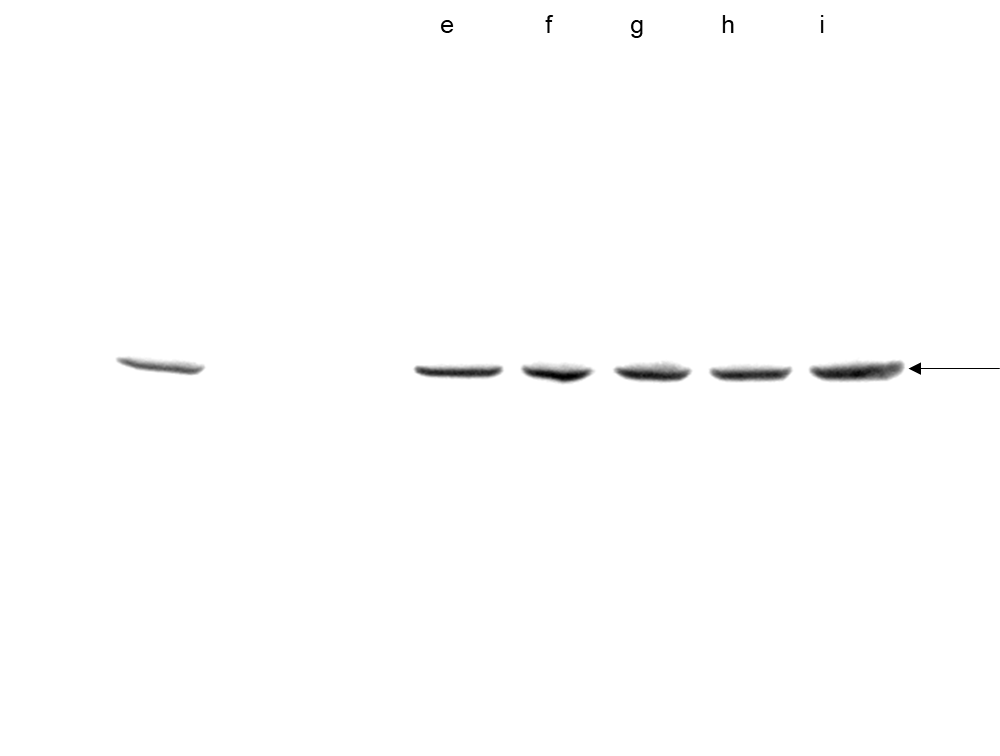


CHOP


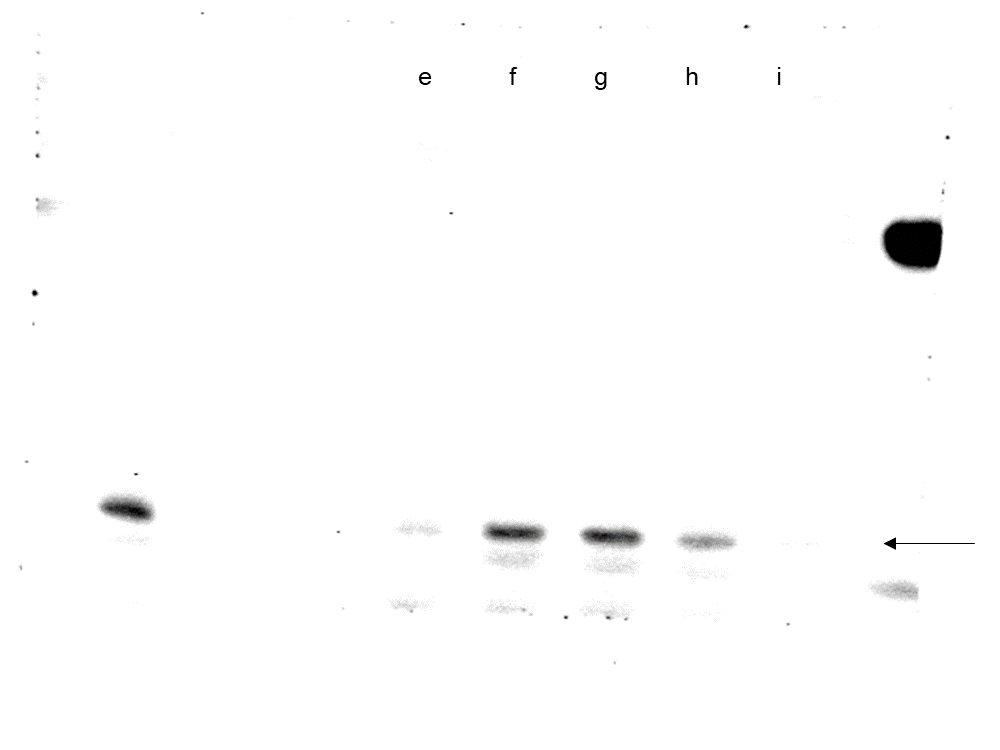


Tubulin

CHOP

Tubulin

CHOP

Tubulin
